# Coupling DNA intercalation with redox catalysis: selective killing mechanism of glioblastoma by daidzin

**DOI:** 10.1093/nar/gkag397

**Published:** 2026-04-30

**Authors:** Shreya Banerjee, Sayan Paul, Ranabir Majumder, Debolina Manna, Saugat Mondal, Dhruba Dhar, Souvik Karmakar, Jayasri das Sarma, Abhijit Das, N D Pradeep Singh, Soumen Das, Budhaditya Mukherjee, Anakuthil Anoop, Mahitosh Mandal

**Affiliations:** School of Medical Science and Technology, Indian Institute of Technology, Kharagpur 721302, India; Department of Chemistry, Indian Institute of Technology Kharagpur, Kharagpur 721302, India; School of Medical Science and Technology, Indian Institute of Technology, Kharagpur 721302, India; Department of Physiological Sciences, Oklahoma State University, Stillwater, OK 74078, United States; School of Medical Science and Technology, Indian Institute of Technology, Kharagpur 721302, India; Department of Chemistry, Indian Institute of Technology Kharagpur, Kharagpur 721302, India; School of Medical Science and Technology, Indian Institute of Technology, Kharagpur 721302, India; Department of Biological Science, Indian Institute of Science Education and Research, Kolkata, Mohanpur, 741 246 West Bengal, India; Department of Biological Science, Indian Institute of Science Education and Research, Kolkata, Mohanpur, 741 246 West Bengal, India; Department of Bioscience and Biotechnology, Indian Institute of Technology, Kharagpur 721302, India; Department of Chemistry, Indian Institute of Technology Kharagpur, Kharagpur 721302, India; School of Medical Science and Technology, Indian Institute of Technology, Kharagpur 721302, India; School of Medical Science and Technology, Indian Institute of Technology, Kharagpur 721302, India; School of Digital Sciences, Kerala University of Digital Science, Innovation, and Technology, Technopark Phase IV, Pallipuram, Thiruvananthapuram, Kerala 695317, India; School of Medical Science and Technology, Indian Institute of Technology, Kharagpur 721302, India

## Abstract

Glioblastoma (GBM) is recognized as one of the most treatment-resistant malignancies, owing to its reinforced DNA repair systems and limited drug accessibility across the blood–brain barrier. This study, identifies, daidzin (DZN), a naturally derived isoflavone, as a potent redox-active DNA intercalator that intrinsically combines physical intercalation with chemical reactivity to breach this resistance. Unlike traditional intercalators, DZN autonomously triggers destabilization of DNA helices by inducing torsional strain, thereby producing convergent strand and base lesions through photo-independent redox pathways involving deoxyribose cleavage and C8 guanine oxidation. Additionally, DZN demonstrates pronounced glioma-specific cytotoxicity by initiating ^1^O_2_-driven oxo-cation formation and concomitant H_2_O_2_ production. This redox burst results in DNA strand scission activating robust DDR signaling and oxidative base lesions, which cripple tumor survival. Enhanced membrane fluidity in glioma cells likely facilitates superior DZN permeability, intracellular accumulation, thereby allowing DZN to initiate this robust DNA damage responses, culminating in G1 arrest and apoptosis in GBM cells while sparing normal glia. *In vivo*, DZN markedly suppresses tumor growth and surpasses temozolomide efficacy, current clinical option for GBM treatment. This work, thus, establishes a previously unrecognized paradigm of DNA intercalation-driven redox chemistry, presenting DZN as a promising therapeutic capable of exploiting the genomic frailties to overcome therapeutic resistance in glioma.

## Graphical Abstract

**Graphical Abstract:**
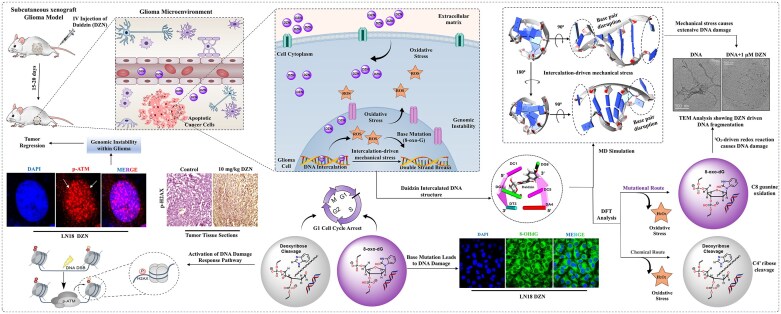

## Introduction

Glioblastoma (GBM), the most aggressive primary brain malignancy, exhibits a hyperproliferative phenotype that imposes substantial stress on DNA replication machinery, often overwhelming repair pathways and leading to constitutive replication stress, resulting in DNA double-strand breaks (DSBs) [1]. These lesions are sensed by the MRN complex (MRE11–RAD50–NBS1) which then recruits and activates Ataxia-telangiectasia mutated (ATM) that subsequently phosphorylates several substrates, notably Histone H2A family member X (H2AX) at Serine 139, producing phosphorylated Histone H2AX (p-H2AX), a marker for DSBs [2]. ATM also phosphorylates checkpoint kinases CHK1 and CHK2, leading to cell cycle arrest, that enables DNA repair [3]. However, if the damage is irreparable, pathways leading to apoptosis are activated [4]. Moreover, glioma cells exhibit elevated basal reactive oxygen species (ROS) due to mitochondrial dysfunction [5], though this persistent oxidative stress is typically balanced by adaptive upregulation of antioxidant defenses e.g. glutathione peroxidase, superoxide dismutase, catalase etc., but it makes GBM cells particularly prone to oxidative damage from subsequent ROS stress [6]. DNA intercalators and ROS-inducing agents can exploit this imbalance, inducing oxidative DNA lesions such as 8-oxo-2′-deoxyguanosine (8-oxo-dG) [7], which interfere with base-pairing fidelity and promote genomic collapse [8].

Despite these insights, conventional therapies in glioma have failed to produce durable responses, largely due to tumor heterogeneity, blood–brain barrier (BBB), and resistance mechanisms [9]. Temozolomide (TMZ), the standard chemotherapy drug for glioma methylates DNA at the O^6^ position of guanine and this lesion mis-pairs with thymine, triggering mismatch repair (MMR)-dependent apoptosis [10]. However, in tumors with unmethylated O^6^-Methylguanine-DNA Methyltransferase (MGMT) promoters or elevated MGMT protein levels, these lesions are rapidly repaired, leading to intrinsic TMZ resistance. Attempts to inhibit MGMT or bypass its effects have seen limited success due to toxicity or compensatory DNA repair mechanisms [11]. Other potent DNA damaging agents, such as Topoisomerase II poisons like doxorubicin (DOX) and daunorubicin (DRN) are potent intercalators that induce DSBs, show efficacy in systemic malignancies but fail in GBM due to several convergent barriers e.g. poor BBB penetration, efflux by ATP-binding cassette (ABC) transporters (notably P-glycoprotein), etc. [12]. Similarly, anti-angiogenic agents such as bevacizumab transiently normalize vasculature and reduce edema but do not improve overall survival [13], likely due to adaptive hypoxia-driven resistance and invasion [14]. Targeted therapies against Epidermal Growth Factor Receptor (EGFR), Isocitrate dehydrogenase (IDH) mutations have also underperformed due to tumor heterogeneity, compensatory signaling loops, and limited drug access to the central nervous system (CNS) [15, 16]. Thus, the search for next-generation DNA intercalators that robustly induce DSBs and potentiate ROS-mediated cytotoxicity while circumventing established resistance pathways is imperative for advancing GBM therapeutics. For instance, the co-administration of DNA intercalators with inhibitors of DNA Damage Response (DDR) components, such as Ataxia Telangiectasia and Rad3-related protein (ATR) or Poly (ADP-ribose) polymerase (PARP) inhibitors, has been proposed to amplify DNA damage beyond the cellular repair capacity, forcing apoptotic commitment in tumor cells [17]. Complementary strategies that combine potent intercalators with agents targeting redox regulators may further shift the oxidative balance beyond the threshold tolerated by GBM, thereby enhancing cell death [18].

Natural flavonoids have long been investigated as bioactive scaffolds with the capacity to intercalate DNA and modulate oxidative balance, properties that align well with the vulnerabilities of glioblastoma. Within this class, the glycosylated isoflavone daidzin (DZN) [19], derived from *Pueraria lobata*, has drawn attention for its structural planarity, redox activity, and reported ability to access the central nervous system. These attributes suggest that DZN may represent a pharmacologically distinct dual-function compound with therapeutic potential against glioma. Unlike conventional anthracycline intercalators, DZN offers key advantages that enhance its therapeutic profile. Its planar isoflavone scaffold enables π–π stacking with DNA nucleobases, promoting stable intercalation within the DNA helix [20]. DZN also benefits from favorable pharmacokinetic attributes e.g. low molecular weight and moderate lipophilicity [21], allowing better central nervous system (CNS) access compared to traditional topoisomerase poisons. Preliminary studies suggest that DZN can cross the blood–brain barrier (BBB) [22] and modulate redox homeostasis, inducing ROS [23] in cancer cells that exist under heightened oxidative stress, thereby augmenting cytotoxicity.

Hence, our study investigates the DNA-damaging potential and glioma-specific cytotoxicity of DZN, as a prospective therapeutic agent for glioblastoma. Previous molecular docking and molecular dynamics (MD) simulations have established that DZN intercalates between DNA base pairs, particularly within GC-rich regions, with binding energies comparable to the reference intercalator DRN [20]. However, DNA intercalation alone is not sufficient to establish genotoxic activity, as illustrated by riboflavin, another flavonoid that intercalates into DNA but fails to induce damage in the absence of external energy excitation [24]. This limitation underscores the need to clarify whether DZN functions merely as a DNA intercalator or as a potent DNA-damaging agent.

To address this mechanistic uncertainty, we employed a multi-scale integrative approach that combines *in silico, in vitro*, and *in vivo* methodologies. At the computational level, we performed MD simulations to understand the crucial role of the intercalation of DZN in the structural perturbations of DNA strands. Furthermore, we evaluated the intrinsic reactivity of DZN toward DNA and mapped plausible reaction mechanisms under the light of density-functional theory (DFT). At the biophysical level, we used low-dose transmission electron microscopy (TEM), UV–visible absorption, and circular dichroism (CD) spectroscopy to monitor DNA conformational perturbations. In cell-based models, we interrogated the onco-selective toxicity profile of DZN, DSB formation, ROS induction, cell cycle analysis, etc. For translational assessment, we conducted efficacy studies in a Sprague–Dawley rat glioma model alongside toxicological evaluation of major organs.

This study is designed to evaluate DZN as a dual anticancer agent with DNA-intercalative and redox-responsive property that exploits glioma-specific vulnerabilities, and delineate a structure–activity landscape to guide lead optimization toward lower toxicity, higher blood–brain-barrier penetration, and greater DNA-damage potency, thereby enabling the rational development of next-generation glioma therapeutics.

## Materials and methods

### Structural and conformational analysis using X3DNA

Initially, we performed advanced analysis of the DNA–ligand complex using the 300 ns trajectories from our earlier molecular dynamics study [20]. To assess the structural perturbations induced by ligand binding, we performed a comprehensive conformational analysis using the do_x3dna tool [25, 26].

The local density and spatial reorganization of DNA upon ligand binding was analyzed through radial distribution functions (RDFs), comparing the ligand–DNA atom distances. Similarly, hydrogen bond (H-bond) count was analyzed between the ligand-DNA complexes. Local base pair parameters (shear, stretch, stagger, buckle, propeller, and opening) were obtained from the outputs of x3dna and plotted to assess how ligand binding altered the local base pair geometry. Parameters such as shift, slide, rise, tilt, roll, and twist were compared between the ligand-bound DNA systems to evaluate local stacking and helical flexibility changes. Helical descriptors including helical twist, inclination, tip, and *X/Y*-displacement were also extracted from x3dna output files and analyzed to determine global DNA structural changes. The processed output data were plotted using GraphPad prism [27] and matplotlib [28] across the systems. All DNA–ligand interaction structures and representative frames were visualized using UCSF Chimera [29].

### DFT calculations using ORCA

**Figure 1.**
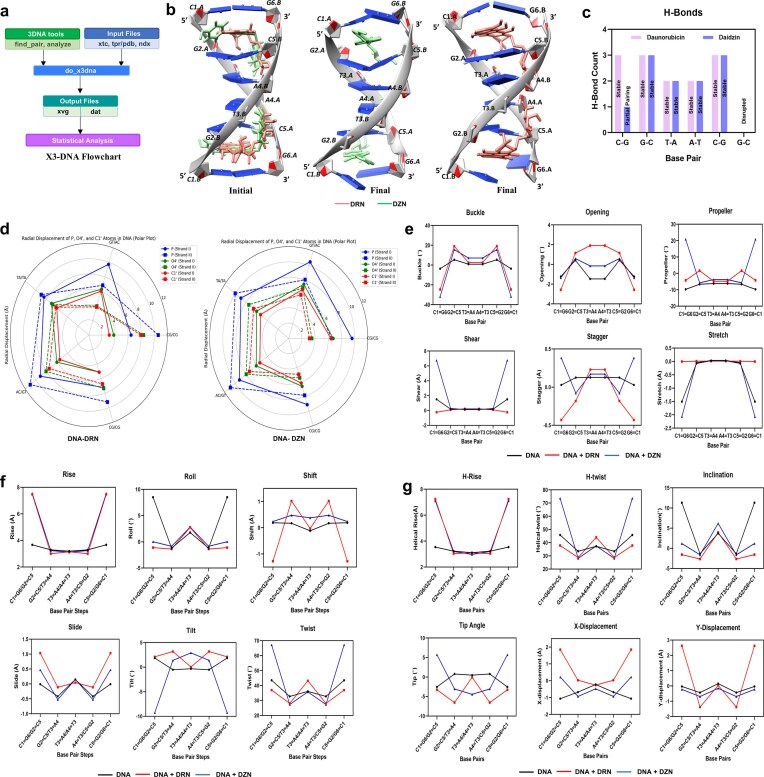
DNA structural perturbations elicited by DZN and DRN. (**a**) Workflow for extracting DNA structural descriptors from MD trajectories using do_x3dna. Input trajectories and topology files are processed with 3DNA tools to generate .dat outputs containing base-pair (buckle, shear, propeller, opening, stagger, and stretch), base-step (rise, roll, shift, slide, tilt, and twist), and helical parameters (H-rise, H-twist, inclination, tip, and *X*- and *Y*-displacements), which are subsequently subjected to statistical analysis. (**b**) Initial and final MD snapshots of the intercalative complexes are shown using UCSF Chimera. Left: starting structures with both ligands inserted (DZN, green; DRN, salmon). Middle: final snapshot of DNA–DZN (DZN, green). Right: final snapshot of DNA–DRN (DRN, salmon). (**c**) Time-averaged counts of hydrogen bonds formed between each ligand (DRN, light pink; DZN, blue) and DNA are shown in a bar plot. (**d**) Polar plots of radial displacement of P, O4′, and C1′ atoms in DNA. DRN (left) causes larger, asymmetric shifts, whereas DZN (right) induces smaller, more uniform perturbations. (**e**) Average intra–base-pair deformations for the ligand-bound duplexes relative to free DNA, showing ligand-induced changes in buckle, shear, propeller, opening, stagger, and stretch (free DNA, black; DNA–DRN, red; DNA–DZN, blue). These metrics report distortions in base-pair planarity, hydrogen-bond geometry, and in-/out-of-plane translations. (**f**) Average base-step parameters comparing DNA–DRN and DNA–DZN to free DNA (free DNA, black; DNA–DRN, red; DNA–DZN, blue), capturing intercalation-driven widening (rise), backbone-coupled bending (roll/tilt), lateral translations (shift/slide), and changes in helical winding (twist). (**g**) Average helical descriptors (H-rise, H-twist, inclination, tip, and *X*- and *Y*-displacements) for free DNA (black), DNA–DRN (red), and DNA–DZN (blue), summarizing global axis repositioning, helix winding/unwinding, and translational offsets of the helical axis within the DNA base-pair.

All quantum-chemical calculations were performed with ORCA 5.0.3 [30, 31]. Initial structures of the intercalated DNA–daidzin (DNA–DZN) complex were extracted from the 300-ns MD trajectory (previous work [20]) in which DZN is intercalated at the GC step C1–G6/G2–C5 and forms a hydrogen bond to G6 (Fig. 1). Geometry optimizations and vibrational frequency analyses were carried out at PBE-D4/def2-SVP [32, 33] level of theory with resolution-of-identity (RI-J) approximation [34] and def2/J auxiliary basis set [35] for speed and accuracy; stationary points were verified by frequency analysis (no imaginary frequency; NImag = 0) at the respective potential energy surfaces of the minima [36, 37]. Improved single-point energies at the optimized geometries were computed in solution phase using TPSS-D4 [38, 39]/def2-TZVPP^36,38^ with def2-TZVPP/C auxiliary basis set and RI-J approximation for speed and accuracy. SMD (water, ε = 80.4) solvation method [40] was considered in this purpose. The complex was treated as a singlet tetra-anion, **¹ [DNA–DZN]^4−^**. Multiwfn [41] was used to carry out the density of states (DOS) and noncovalent interaction (NCI) analyses. The cartesian coordinates of the optimized structures are appended in Supplementary Table S10. Detailed computational procedure is discussed in the supporting information (see Computational Details in supplementary section).

### Chemicals and reagents

DZN (PHR2623), TMZ (PHR1437), DOX (D1515), and calf thymus DNA (CTDNA) (D1501) were procured from Sigma–Aldrich. The primary antibodies were obtained from Abclonal and Cell Signaling Technology, USA: β-actin (Catalog #3700T CST), p-ATM (Catalog #4526T CST), H2AX (Catalog #7631T CST), p-H2AX (Catalog #9718T CST), CHK2 (Catalog # 2662T CST), p-CHK2 (Catalog # 2179T CST), Bak (Catalog # 3814S CST), Bax (Catalog #A0207 Abclonal), XIAP (Catalog # 2042T CST), and Bcl-xL (Catalog # 2764T CST). Secondary antibodies used for immunoblotting and immunohistochemistry were anti-mouse IgG (CST, 7076P2) and anti-rabbit IgG (CST, 7074P2). Fluorescent tagged primary antibody against 8-OHdG (E-8) FITC (Catalog # sc-393871 FITC) was purchased from Santa Cruz Biotechnology. One-step TUNEL *in situ* apoptosis kit (Catalog No: E-CK-A320) was purchased from ElabScience. Fluorescent tagged secondary antibody (Alexa Fluor™ 647 anti-mouse IgG) was purchased from Thermo Fisher Scientific. All other chemicals and reagents, including thiazolyl blue tetrazolium bromide (M5655) and bovine serum albumin (BSA) (MB083), Calcein AM (Sigma, 17783), propidium iodide (PI; Sigma, P4170), H2DCFDA (D399), and Hoechst 33342 (Invitrogen, H3570) were obtained from Sigma–Aldrich, Thermo Fisher Scientific, and Himedia. ECIS-8W1E DD was purchased from Applied BioPhysics, USA.

### UV–Vis spectroscopy

UV–Vis absorption spectra were recorded to investigate the interaction of DZN and DOX with CTDNA. CTDNA (50 µg ml^⁻1^) was prepared in 1× Phosphate-Buffered Saline (PBS) (pH 7.4). Stock solutions of DZN and DOX (100 µM) were freshly prepared in the same buffer. Spectral measurements were performed on a double-beam UV–visible spectrophotometer equipped with a temperature-controlled cell holder. Titrations were carried out by incrementally adding DZN (0–200 µl) to a fixed DNA solution (total volume adjusted to 1 ml). The same titration series was repeated with DOX as a positive control for DNA fragmentation and spectral perturbation. For each addition, spectra of the corresponding free compound (without DNA) were recorded under identical conditions to enable background subtraction. Each measurement was performed in triplicate using independently prepared samples. Buffer spectra were subtracted from all data prior to analysis. The resulting spectra were evaluated for changes in DNA absorbance at 200–260 nm, the appearance of shifts indicative of base stacking perturbation or DNA fragmentation. DOX-treated DNA served as a positive reference for this experiment.

### CD spectroscopy

Circular dichroism (CD) spectra were obtained to probe conformational alterations in B-form CTDNA upon interaction with DZN. Samples contained CTDNA (50 µg ml^⁻1^) in 1× PBS (pH 7.4) and were analysed alone or following addition of DZN (final concentrations: 5 and 10 nM). DOX (10 nM) was used as a positive control for B-DNA perturbation. Spectra were recorded in the range of 190–350 nm using 1 mm pathlength quartz cuvettes. Buffer and compound-alone spectra were subtracted to remove background or induced CD contributions. The CD spectrum of native CTDNA exhibited a characteristic positive band near 275 nm and a negative band near 245 nm, corresponding to the right-handed B-DNA conformation. Following incubation with DZN or DOX (1–2 min at room temperature), variations in ellipticity and wavelength shifts at these bands were monitored to assess conformational transitions.

### Transmission electron microscopy analysis

Low-dose transmission electron microscopy (TEM) was employed to visualize DNA morphology and assess structural integrity following treatment with DZN or DOX. Samples were prepared in 1× PBS (pH 7.4) containing CTDNA (50 µg ml^⁻¹^) alone, CTDNA + 1 µM DOX (positive control for fragmentation), CTDNA + DZN at 0.05, 0.1, or 1 µM. Mixtures were incubated for 20–30 min at room temperature prior to grid preparation. A 3–4 µl aliquot of sample was applied to each grid for 1 min and blotted gently. Grids were air-dried in a dust-free environment. TEM imaging was performed on a cryo-TEM under low-dose conditions. Several micrographs were collected per grid across three independent grids per sample condition. Image analysis was performed using Fiji/ImageJ software.

### TUNEL assay

Glioma cell lines (LN18 and LN229) were initially seeded onto coverslips, and upon cell attachment, the culture was supplemented with complete Dulbecco’s Modified Eagle Medium (DMEM). Upon reaching 60% confluency, the cells underwent treatment with DZN, with one set serving as a control. Subsequently, the cells were incubated for 24, 48, and 72 h. Next, we initiated our TUNEL assay employing the one-step TUNEL *in situ* apoptosis kit (Catalog No: E-CK-A320) as per the manufacturer’s instructions. The process began with the preparation of cell slides, involving a single wash with PBS and careful removal of moisture using filter paper. The slides were then immersed in a fixative buffer at room temperature for 15–20 min. Post-fixation, the slides underwent washing with PBS, followed by incubation in a self-prepared permeabilization buffer at 37°C for 10 min. Subsequently, the samples were treated with TdT equilibration buffer and incubated at 37°C for 10–30 min. Labeling working solution (TdT equilibration buffer, TdT enzyme, and FITC-dUTP) was applied, and the slides were incubated at 37°C for 60 min in a shaded, humidified chamber. Finally, the slides were treated with a 4′,6-diamidino-2-phenylindole (DAPI) working solution at room temperature for 5 min in shaded light, washed with PBS, and analyzed under a fluorescence microscope using an appropriate filter.

### Cell culture

The noncancerous cell lines (SVGP12), human glioma cancer cell lines (LN18, LN229, and U87MG), and rat glioma cell line (C6) were acquired from the National Centre for Cell Science (Pune, India). The growing medium was prepared using Sigma–Aldrich’s 1% penicillin–streptomycin, Gibco’s 10% fetal bovine serum (FBS), and Eagle’s Minimum Essential Medium (EMEM: Sigma–Aldrich) specifically for SVGP12, Dulbecco’s Modified Eagle’s Medium (DMEM, Gibco). The cells were cultured in T-25 flasks at 37°C in a humidified CO_2_ (5%) incubator until they were about 80% confluent. Primary astrocyte cultures were prepared from neonatal C57BL/6 mouse pups (postnatal days 0–1) using a modified version of protocols routinely followed in the laboratory and previously described in the literature [42–45]. All animal procedures were approved by the Institutional Animal Ethics Committee (IAEC, Protocol No: IISERK/IAEC/S-UPM-AP/2026/SL6). Briefly, neonatal pups were decapitated, and whole brains were carefully removed under sterile conditions. After removing the skull, the meninges were carefully separated. The tissues were then transferred to cold Hank’s Balanced Salt Solution (HBSS, Gibco) and mechanically minced using a 5 ml serological pipette. Tissue dissociation was enzymatically performed in HBSS containing trypsin (Sigma–Aldrich) (10 mg/ml stock; 0.25 ml per brain) and DNase I (Sigma–Aldrich) (0.1 mg/ml stock, 0.25 ml per brain) to minimize cell clumping caused by released DNA. The digestion was performed for 30 min at 37°C in a gently rocking water bath. Following enzymatic digestion, the tissue was gently triturated using a 5 ml serological pipette in the presence of FBS (Gibco) (0.25 ml per brain) to inactivate trypsin and support cell viability. The resulting cell suspension was centrifuged at 600 × *g* for 10 min, and the pellet was resuspended in HBSS. To remove undigested fragments and debris, the suspension was passed through a 70 μm nylon mesh filter and centrifuged again under the same conditions. The final cell pellet was resuspended in complete growth medium consisting of DMEM (Gibco) supplemented with 10% FBS, 1% nonessential amino acids, 0.1% L-glutamine (Gibco), and 1% penicillin–streptomycin (Gibco). Cells were seeded into culture flasks and maintained at 37°C in a humidified incubator with 5% CO_2_. After 24 h, non-adherent cells were removed by replacing the media. Mixed glial cultures were then maintained with media changes every 2–3 days until they reached confluency, typically within 9–10 days. To enrich for astrocytes, confluent mixed glial cultures were maintained without media changes for an additional 7–10 days to facilitate differential adhesion. Subsequently, flasks were placed on an orbital shaker at 180 rpm for 45 min at 37°C. This step selectively detached loosely adherent microglia, while the more adherent astrocytes remained as a monolayer. The media containing detached microglia was discarded, and the adherent cells were retained as enriched primary astrocyte cultures.

### MTT assay

The MTT assay was performed following a previously established protocol with minor modifications [46, 47, 48]. The cytotoxic effects of DZN and the standard anti-glioma drug TMZ were evaluated using the MTT assay in glioma cell lines (LN18, LN229, and U87MG) and noncancerous glial cells (SVGP12) and primary astrocytes in a dose-dependent manner. Cells were seeded in 96-well plates at a density of 1 × 10^5^ cells/ml (100 μl per well) in their respective complete growth media (DMEM for LN18, LN229, U87MG, and EMEM for SVGP12; astrocyte-specific media for primary astrocytes) and incubated overnight at 37°C in a humidified atmosphere with 5% CO_2_ to allow cell attachment. Primary astrocytes were cultured for an additional period (up to 7 days) with media replacement every 3 days prior to treatment to ensure proper stabilization and growth.

Following incubation, cells were treated with increasing concentrations of DZN or TMZ (vehicle: PBS) prepared in the respective culture media and incubated for 48 h under standard conditions. After treatment, the culture medium was replaced with MTT solution (Sigma–Aldrich) at a final concentration of 0.5–1 mg/ml, depending on cell type, and incubated for 4–6 h at 37°C to allow formation of formazan crystals. Subsequently, the MTT-containing medium was carefully removed, and the insoluble formazan crystals were dissolved in dimethyl sulfoxide (DMSO, 100 μl per well). Absorbance was measured at 590 nm using a microplate reader (Bio-Rad iMark^™^, BioTek Epoch Microplate Spectrophotometer). Each condition was performed in multiple technical replicates, and all experiments were independently repeated at least three times.

### Cell cycle analysis by flow cytometry (FACS)

To assess the effect of DZN on cell cycle progression, flow cytometric analysis was performed on LN229 glioma cells treated with DZN. Cells were seeded in six-well plates at a density of 2 × 10^5^ cells per well and allowed to adhere overnight. The experimental groups included untreated control, and cells treated with DZN at IC_50_ concentrations for 24 and 48 h. At each time point, both adherent and floating cells were collected, washed twice with cold PBS, and fixed in 70% ethanol at −20°C overnight. Following fixation, cells were centrifuged, washed with PBS, and incubated with a staining solution containing 50 μg/ml PI and 100 μg/ml RNase A for 30 min in the dark at room temperature. Samples were acquired using a flow cytometer (e.g. BD FACS Calibur, BD Biosciences, Franklin Lakes, NJ), and data were analyzed using FlowJo software to quantify the distribution of cells across G0/G1, S, and G2/M phases, as well as the sub-G1 population indicative of apoptosis.

### Scratch assay

To evaluate the impact of DZN on glioma cell migration, a wound healing (scratch) assay was conducted in LN18 and LN229 cell lines. Cells were seeded in six-well plates and cultured until a confluent monolayer was formed. A uniform scratch was created across the monolayer using a sterile 200 μl pipette tip, followed by gentle washing with PBS to remove detached cells. Cells were then treated with DZN at IC_50_ concentrations, and the media was replaced with serum-free DMEM to minimize proliferation-associated interference. Images were captured at 0 h (immediately after scratching) and at 24, 48, and 72 h post-treatment using a phase-contrast microscope at fixed positions. The wound closure area was quantified using ImageJ software, and migration rates were expressed as the percentage of wound closure relative to the initial scratch width. Untreated controls were included for comparison at each time point.

### Live-dead assay

Cells were cultivated in 35-mm petri dishes and subjected to treatment with DZN at IC_50_ dose for varying durations: 0, 24, and 48 h. Following the completion of the respective incubation periods, each 35-mm Petri dish received an addition of 1 μM Calcein AM (Sigma, 17783) and 2 μg/ml PI (Sigma, P4170). This mixture was then incubated for 30 min within a humidified environment containing 5% CO_2_ and maintained at 37°C. Subsequently, the cells were observed using a fluorescence microscope (Nikon Eclipse TS2, Japan).

### Intracellular ROS measurement by DCFDA assay

To examine whether drug treatment induced oxidative stress in different cell lines, intracellular ROS levels were measured using the DCFDA assay following previously published protocols with minor modifications [46]. Intracellular ROS levels were measured using the DCFDA (2′,7′-dichlorofluorescin diacetate) assay in glioma cell lines (LN18 and LN229), noncancerous glial cell line (SVGP12) and primary astrocytes. Cells were seeded on sterile glass coverslips in 24-well plates (for LN18, LN229, and SVGP12; 1 × 10^5^ cells per well) or in appropriate culture plates for primary astrocytes and allowed to reach ∼80%–90% confluency under standard conditions (37°C, 5% CO_2_). For glioma cells, treatment was performed using DZN at the respective IC_50_ concentrations for 48 h, with untreated cells serving as controls. LN18 and LN229 cells were treated with 75 µM DZN and 100 µM DZN, respectively, for 48 h. SVGP12 cells were treated with 200 µM DZN for 48 h. Primary astrocytes were treated with DZN (100 and 200 µM) for 48 h, alongside untreated controls. *tert*-Butyl hydroperoxide (TBHP; Sigma–Aldrich)–treated cells were included as a positive control to validate ROS detection. Following treatment, cells were washed with 1× PBS and incubated with 10 µM DCFDA (Sigma–Aldrich) in serum-free medium for 30 min at 37°C in the dark. Excess dye was removed by washing with PBS. For glioma cells, samples were fixed with 4% paraformaldehyde for 15 min prior to mounting, whereas primary astrocytes were imaged live immediately after staining. Fluorescence images were acquired using an epifluorescence microscope (Nikon Eclipse TS2) equipped with a FITC filter set (excitation/emission ∼495/529 nm). Intracellular ROS levels were assessed based on green fluorescence intensity and compared across control and treated groups.

### Electrical bioimpedance spectroscopy

The noncancerous cell line (SVGP12) and human glioma cancer cell lines (LN18 and LN229) cultured in T25 flasks till 80% confluence were trypsinized using 0.25% trypsin, followed by suspending the obtained cell pellet in 1× PBS at a concentration of 10^6^ cells/ml. 400 µl of the corresponding cell suspensions were subjected to impedimetric measurements using an ECIS-8W1E DD (Applied BioPhysics, USA) cell culture well platform. For measuring the baseline, impedimetric responses were acquired with 400 µl of PBS. The ECIS-8W1E DD culture well platform has eight distinct mini-culture wells, each featuring a unique circular working electrode and a shared counter-electrode constructed from a thin gold film, as previously detailed [49]. The impedance data from the ECIS device were recorded using an Agilent precision impedance analyzer 4294-A, operating in the frequency range of 40 Hz-100 MHz, by applying a 15-mV AC excitation voltage.

### Western blot analysis

Protein expression analysis through western blotting was performed on glioma cells exposed to various treatment conditions for 48 h, following a previously reported protocol [50]. LN18 and LN229 cells were grown in 100 ml plates and treated with DZN for 48 h. Next, the cells were washed with PBS and lysed using RIPA buffer (Invitrogen Corporation, USA) supplemented with a 1× protease inhibitor cocktail (Roche, Basel, Switzerland) and 1× phenylmethylsulfonyl fluoride (PMSF). Equal amounts of protein (40 µg) from each sample were separated by SDS–polyacrylamide gel electrophoresis (SDS–PAGE) and transferred onto nitrocellulose membranes. Membranes were blocked with 3% BSA in PBS at room temperature for 2 h, followed by overnight incubation at 4°C with primary antibodies diluted 1:1000 in blocking buffer. The primary antibodies used were specific for β-actin (Catalog #3700T CST), p-ATM (Catalog #4526T CST), H2AX (Catalog #7631T CST), p-H2AX (Catalog #9718T CST), CHK2 (Catalog # 2662T CST), p-CHK2 (Catalog # 2179T CST), Bak (Catalog # 3814S CST), Bax (Catalog #A0207 Abclonal), XIAP (Catalog # 2042T CST), and Bcl-xL (Catalog # 2764T CST). After incubation, membranes were washed five times with PBST (PBS containing 0.1% Tween-20) for 10 min each and subsequently incubated with appropriate horseradish peroxidase (HRP)-conjugated secondary antibodies for 1 h at room temperature. Protein bands were visualized using a ImageQuantTM LAS 500 (GE Healthcare), and densitometric analysis was performed using Image Lab software (Bio-Rad).

### Immunofluorescence

Glioma cell lines (LN18 and LN229) seeded on coverslips were treated with IC_50_ dose of DZN. The cells were fixed with 4% paraformaldehyde and treated with 0.1% Triton-X-100. After the blocking step with 3% BSA, cells were incubated with primary antibodies 8-OHdG Antibody (E-8) FITC (Catalog # sc-393871 FITC) and p-ATM (1: 1000 in PBS) for 3 h at room temperature. Subsequently, cells were washed with PBS and incubated with the secondary antibodies (Alexa Fluor^™^ 647 anti-mouse IgG) for 2 h at room temperature. Finally, Cells were counterstained with DAPI and mounted over glass slides. Cells were imaged by a super-resolution confocal microscope (Olympus FV-3000).

### Hoechst staining assay

The Hoechst assay was conducted according to manufacturer’s protocol. Briefly, following treatment with DZN in a time dependent manner, LN18 and LN229 cells were incubated with Hoechst 33342 (Invitrogen, H3570) for 5 min in a 37°C humidified incubator with 5% CO_2_. After incubation, the media was removed, cells were washed with PBS and visualized using inverted fluorescence microscope (Nikon Eclipse TS2, Japan).

### Subcutaneous glioma model

We used female Sprague Dawley rats (80–100 g; 6–8 weeks) for the experiment. The animals were maintained, and the experiment was carried out with the approval of the Institutional Animal Ethical Committee . The animals were kept for one week in the quarantine facility of the animal house for acclimatization purposes. The subcutaneous glioma model was developed following the protocol reported earlier [51]. After tumor development, the animals were divided into different groups (8 animals in each group). Animals were injected with TMZ and/or DZN intravenously every alternate day for 14 days. Animals were then sacrificed to collect the tumors, and the final tumor volume was measured. The animal experiment was approved by the Animal Ethics Committee (IE-02/MM-SMST/1.22).

### Software and statistical analysis

Data analysis was performed using Image Lab^™^, ImageJ, FlowJo, EIS Spectrum Analyser, matplotlib, Jmol, ChemDraw, Origin and GraphPad Prism software. All experiments were conducted in triplicate unless otherwise stated, and data are expressed as the mean ± standard deviation (SD). Statistical analyses were carried out using the Student’s *t*-test, with *P* < 0.05 considered statistically significant. Statistical significance is indicated as follows: *P* < 0.05 (*), *P* < 0.01 (**), *P* < 0.001 (***), and *P* < 0.0001 (****). DFT calculations were performed using ORCA 5.0.3 software and subsequent analysis (DOS and NCI) were carried out using Multiwfn software. Jmol and ChemDraw are used to illustrate the DFT optimized structures and mechanistic scheme. Gibbs free energy values of the species are calculated by adding the respective solvent phase corrected electronic energy (at 0 K) with the gas-phase correction term (at 310 K or 37°C) (Supplementary Table S7) obtained from the optimization and frequency calculation. In line with the approach of Westwood *et al*. [52], reaction equilibrium constants (*K*_eq_) were evaluated.

## Results

### Differential DNA intercalation dynamics of daidzin versus daunorubicin: comprehensive structural perturbation analysis unveils enhanced buckling, torsional strain, and helical realignment compared to daunorubicin

To evaluate the differential impact of two DNA-binding small molecules DRN, a well-characterized anthracycline intercalator, and DZN, a novel isoflavone under investigation, we employed a comprehensive analysis workflow combining molecular dynamics (MD) simulations with 3DNA-based helical and base pair parameter evaluations (Fig. 1a) [20]. While both ligands are hypothesized to interact through intercalation, their structural dynamics, base pair disruption patterns, and helical geometry consequences diverge substantially, offering mechanistic insight into their distinct DNA-binding behaviors.

### Intercalative binding modes of DRN and DZN reveal distinct helical responses

Final MD snapshots (Fig. 1b) show distinct ligand-induced structural effects on the DNA helix. DRN intercalates deeply between adjacent base pairs, consistent with its known mechanism of planar stacking. This insertion leads to localized helical unwinding and groove expansion, as evidenced by asymmetric radial displacement of backbone atoms (Fig. 1d). In contrast, DZN adopts a more flexible and shallower intercalative pose, maintaining overall helical integrity while inducing subtle, progressive distortions across multiple base-pair steps.

Hydrogen bond analysis (Fig. 1c) further supports these observations, revealing significant base pair disruption, complete loss of canonical hydrogen bonds at the intercalation site, with DZN producing particularly severe disruption at the GC region.

### Hydrogen bonding and radial displacement analyses reinforce these distinct binding modes

For DRN, loss of canonical base-pair hydrogen bonds (Fig. 1c) coincides with large, asymmetric radial displacements of backbone and sugar atoms (Fig. 1d and Supplementary Table S1), confirming that deep intercalation produces local groove widening and helical bending. By contrast, DZN induces smaller, more distributed displacements across the duplex but destabilizes the hydrogen bond interaction between the bases at multiple locations. Collectively, the findings indicate that DRN rigidly locks the DNA helix, while DZN allows more flexible geometric adjustments but causes greater hydrogen bond disruption than DRN upon duplex binding.

### Radial displacement profiling captures ligand-specific helical unwinding and groove expansion

To quantify structural perturbations induced by ligand binding, radial displacement plots (Fig. 1d and Supplementary Table S1) were generated by averaging positional deviations of key backbone (OP1, OP2) and sugar (C1′) atoms across DNA base pair steps. DRN induces pronounced displacement at specific dinucleotide steps, particularly CG/CG (step 1), GT/AC (step 2), and TA/TA (step 3), consistent with deep intercalation that disrupts the local helical architecture. In contrast, DZN shows elevated radial displacement at the same steps, suggesting a more distributed and elastic deformation along the helix.

### Intra-base pair parameters reveal localized geometrical disruption

To assess how ligand binding perturbs local base pair geometry, we analyzed key intra-base pair parameters—buckle, shear, propeller, opening, stagger, and stretch, averaged over time for each base pair (Fig. 1e and Supplementary Table S2). These parameters reflect base pair planarity, base stacking, and local base separation, respectively, offering insight into conformational strain and helical destabilization induced by ligand binding.

DRN-bound DNA displays a concerted softening of base-pair planarity with partial bending as represented by the significant deviation of average base pair parameters. Average buckle angle shifts from 0.88° to –0.98° (Δ = –1.86°), driven by significant terminal bends (±24.6° at C1 = G6/G6 = C1), indicating that terminal bending contributes to the overall curvature near the intercalation site. Shear decreases from 0.63 to 0.03 Å (Δ = –0.59 Å), consistent with improved in-plane base alignment driven by stacking around the inserted DRN. Propeller angle relaxes substantially from –7.51° to –2.44° (Δ = +5.07°), a hallmark of intercalation that flattens the base pair to accommodate the planar aglycone. Opening angle shifts from negative to slightly positive (–0.76° to +0.16°, Δ = +0.92°) with interior pairs showing +1.15° to +1.91°, consistent with wedge-like separation of the two bases along the H-bond axis. Stagger becomes slightly negative on average (0.09 to –0.13 Å), reflecting out-of-plane displacement associated with local unwinding. Stretch moves toward zero (0.52 Å to +0.01 Å, Δ = +0.52 Å), indicating lengthening along the H-bond vector and partial weakening of base pairing.

DRN imposes classic intercalative geometry with reduced (less negative) propeller, increased opening, and small negative stagger, with pronounced terminal buckling, collectively consistent with base pair flattening and partial strand opening around the binding site.

In contrast, DZN elicits a more heterogeneous and locally severe distortion, dominated by terminal perturbations. Buckle decreases strongly on average (0.88° to –3.00°, Δ = –3.88°) with very large terminal bends (–31.8° at C1 = G6/G6 = C1), exceeding DRN. Shear increases substantially (0.63 to 2.39 Å; Δ = +1.76 Å), driven by pronounced terminal displacements (6.75 Å at C1 = G6/G6 = C1), indicating substantial in-plane sliding at the DNA ends rather than uniform shear across the duplex. Propeller twist crosses from negative to positive overall (–7.51° to +3.78°, Δ = +11.29°). Termini exhibits strong positive propeller (+20.8°), indicating loss of planarity/over-twist; however interior pairs remain moderately negative (≈ –3.8° to –5.7°). Opening shifts slightly toward zero (–0.76° to –0.30°; Δ = +0.45°); interior openings are small (–0.15° to +0.60°) in contrast to DRN. Stagger slightly increases (0.09 to 0.16 Å), reflecting asymmetric out-of-plane shifts and stretch becomes more negative (–0.52 to –0.69 Å, Δ = –0.18 Å), consistent with compression along the H-bond axis rather than separation.

DZN induces sharper local curvature and shear, especially at terminal pairs with a flip to positive propeller twist, modest opening, and increased compressive stretch. This pattern suggests lateral base-pair sliding and helix curvature with partial base-pair opening at the intercalation site.

### Base step parameters highlight helical deformation and mechanical plasticity

To further delineate the helical response to ligand intercalation, we examined base step parameters (rise, roll, shift, slide, tilt, and twist) across the DNA duplex over time (Fig. 1f and Supplementary Table S3). These parameters describe inter-base pair orientation and are highly sensitive to stacking disruptions, bending, and torsional stress induced by ligand binding. In the unliganded duplex, base‐step metrics are characteristic of B-DNA (mean rise 3.42 Å, twist 37.74°, modest positive roll 3.29°, and near-zero shift/slide/tilt), with the terminal d(CG)/d(GC) steps showing higher twist and roll (43.56° and 8.52°).

DRN imposes the canonical intercalative signature, the intercalation step widens markedly (terminal rise 3.67 to 7.48 Å; mean rise 3.42 to 4.82 Å), the helix undergoes net unwinding (mean twist 37.74 to 34.64°; –6.6° at the termini and –4.7° at the adjacent step), and lateral translations reorient the base pairs with positive slide and negative shift at the intercalation site (mean slide −0.14 to +0.38 Å; mean shift +0.12 to −0.32 Å). Roll flattens toward zero (3.29 to −0.43°) and tilt rises modestly (0.48° to 2.13°), consistent with base-pair flattening and wedge-like separation around the inserted chromophore.

DZN also widens the intercalation step to a similar extent (terminal rise 3.67 to 7.54 Å; mean rise 4.93 Å) but produces a distinct geometry, the duplex appears overwound on average (mean twist 37.74° to 44.96°), an effect dominated by a dramatic terminal increase to 67.18° (+23.62°), while the adjacent interior step shows only mild unwinding. DZN yields a modest, positive terminal slide (+0.47 Å) and consistently positive shift across all steps (mean shift +0.36 Å), flattens roll to near zero (mean 0.24°), and induces a pronounced negative terminal tilt (≈ −9.25°; mean tilt − 2.56°). Thus, while both ligands enlarge the intercalation gap, DRN produces widening with unwinding and positive slide/negative shift, whereas DZN produces widening with overwinding, positive shift and axis re-orientation, indicating mechanistically distinct intercalation geometries with different consequences for local DNA mechanics.

### Groove width analysis reveals ligand-specific modulation of helical topology

To investigate how ligand binding influences the global conformation of the DNA duplex, we measured the major and minor groove widths across all base pair steps (Fig. 1g and Supplementary Table S4). Groove dimensions reflect the spatial rearrangement of DNA backbones and are critical for recognizing structural transitions associated with intercalation or groove-binding interactions. In the unliganded duplex, helical parameters are consistent with B-DNA, mean helical rise (H-Rise) 3.33 Å and helical twist (H-Twist) 39.19°, with a modest positive inclination (4.75°) and near-zero tip (−0.59°). The helix is slightly displaced toward negative *X* and *Y* (*X* = −0.74 Å; *Y* = −0.15 Å), and the terminal d(CG)/d(GC) steps show the expected higher twist and inclination (H-Twist 45.83°, inclination 11.33°).

DRN imposes the canonical intercalative signature. The intercalation step widens substantially (terminal H-Rise 3.54 to 7.23 Å), driving a higher global mean H-Rise (3.33 to 4.73 Å). Concomitantly, the helix unwinds (mean H-Twist 39.19° to 35.44°), with marked unwinding at the terminal step (45.83° to 37.87°) and at the adjacent interior step (33.49° to 28.76°), partially offset by overwinding at the central step (37.30° to 43.95°). DRN flattens the helix (inclination 4.75° to −0.89°; tip −0.59° to −3.93°), and repositions the helical axis across the base-pair frame, reversing the sign of both *X*- and *Y*-displacements (*X*: −0.74 to +0.70 Å; *Y*: −0.15 to +0.51 Å). At the terminal step, this lateral re-registration is pronounced (*X*: −1.06 to +1.85 Å; *Y*: −0.03 to +2.63 Å), consistent with insertion of a rigid chromophore and wedge-like separation of adjacent pairs.

DZN also enlarges the intercalation gap (terminal H-Rise 3.54 to 7.11 Å; mean H-Rise 3.33 to 4.72 Å) but produces a distinct helical response dominated by overwinding rather than unwinding. The global mean H-Twist rises to 48.20° (+9.02° versus DNA), driven by a dramatic increase at the terminal d(CG)/d(GC) step (45.83° to 73.63°), while the interior step modestly unwinds (33.49° to 28.12°) and the central step remains near baseline (37.30° to 37.52°). Unlike DRN, DZN largely preserves or slightly increases axial alignment (inclination 4.75° to 1.08°; tip −0.59° to +0.19°), and it does not drive the same positive *Y*-displacement: mean *Y* becomes more negative (−0.15 to −0.40 Å), and the terminal *Y* remains near zero (−0.22 Å). *X*-displacement shifts toward zero (−0.74 to −0.38 Å; terminal −1.06 to +0.22 Å), indicating a smaller lateral shift of the helical axis than observed with DRN.

Taken together, both ligands create a widened intercalation gap at the termini, but they diverge in the ensuing helical reorganization. DRN produces widening with global unwinding, axis flattening, and a large positive Y-shift/X-shift reversal. DZN, in contrast, produces widening with pronounced overwinding [especially at d(CG)/d(GC)], minimal axis flattening, and comparatively small lateral axis translation, indicating a mechanistically distinct intercalation geometry that reorients twist more than transverse positioning of the helix.

To further understand the molecular basis of DZN intercalation and its intrinsic genotoxic potential, we interrogated the adduct-forming reactivity of the DNA–DZN complex using quantum-chemical calculations (DFT). Because many intercalators do not inflict DNA damage in the absence of exogenous photo activators (e.g. riboflavin) [24], we explicitly mapped reaction pathways, energetics, and activation barriers to determine whether DZN can promote covalent/ionic transformations of DNA without external energy input.

### Frontier-orbital hybridization in the intercalated DNA–DZN complex stabilizes the adduct and primes deoxy activation

**Figure 2.**
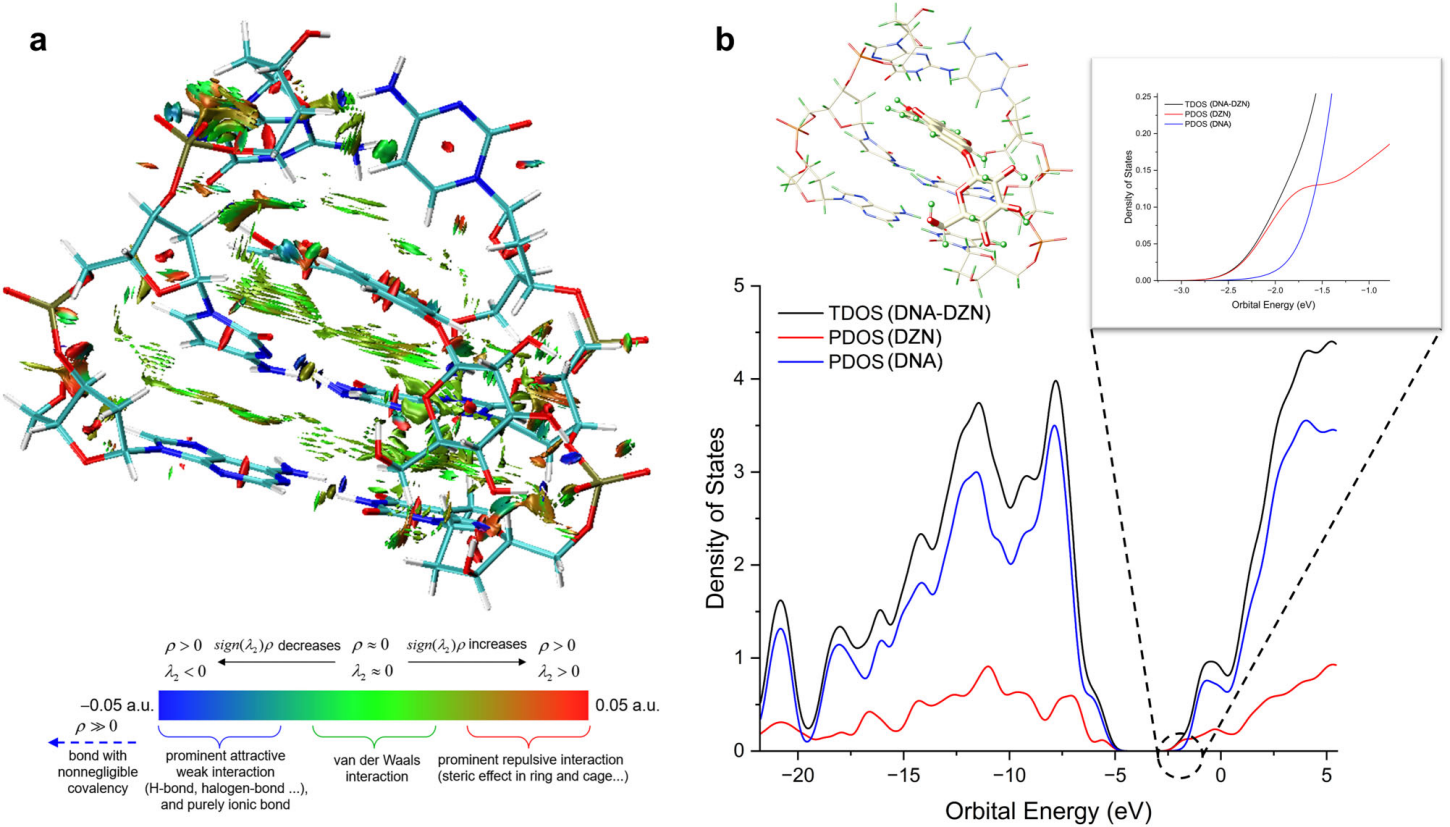
**(a**) NCI plot of the DNA–DZN complex showing green surfaces corresponding to dispersive van der Waals interaction, blue regions indicating hydrogen-bond contributions, and minor red patches for steric repulsion. (**b**) Density of states (DOS) and partial density of states (PDOS) based on fragmentation analysis, computed at the SMD (water)–RI-TPSS-D4/def2-TZVPP//RI-PBE-D4/def2-SVP level of theory, highlights orbital mixing between DNA and DZN. The highest occupied molecular orbital (HOMO) is dominated by DNA contributions (blue), while the lowest unoccupied molecular orbital (LUMO) is primarily DZN-localized (red), supporting the role of DZN as an electron acceptor. Together, these analyses rationalize the stabilization of the intercalated adduct.

To rationalize the stability of the intercalated complex, we analyzed noncovalent interactions at the C1–G6 / G2–C5 step (Fig. 2a). Intercalation reorganizes native DNA π–π stacking, replacing base–base contacts with new π–π interactions between DZN and adjacent bases. Local hydrogen bonds near the intercalation site are weakened due to steric clashes with DZN, while NCI analysis (Fig. 2a and Supplementary Fig. S1) highlights extensive dispersive contacts between DZN aromatic rings and DNA bases. These noncovalent contributions collectively yield a favorable interaction energy (Δ*E*_int_ = −58.5 kcal mol^−1^), supporting strong adduct stabilization.

DOS/PDOS analysis (Fig. 2b) indicates strong orbital mixing between DNA and DZN across the valence spectrum, stabilizing the intercalated adduct. The HOMO of the complex is dominated by DNA orbitals, whereas the LUMO is primarily localized on DZN. This frontier orbital alignment designates DZN as an electron acceptor, enabling charge transfer from neighboring DNA donor groups or nucleophiles in the cellular environment, an interaction absent in canonical DNA. Importantly, the DFT-optimized DNA–DZN structure deviates from native B-DNA, exposing the ribose *sp^3^* C–H bond at C4′. The localization of the acceptor LUMO on DZN polarizes this site, thereby priming C4′ for activation under physiological conditions.

### DFT elucidation of DZN–DNA adduct formation reveals π–π stack disruption, orbital hybridization, and ROS-driven cellular oxidative stress

**Scheme 1.**
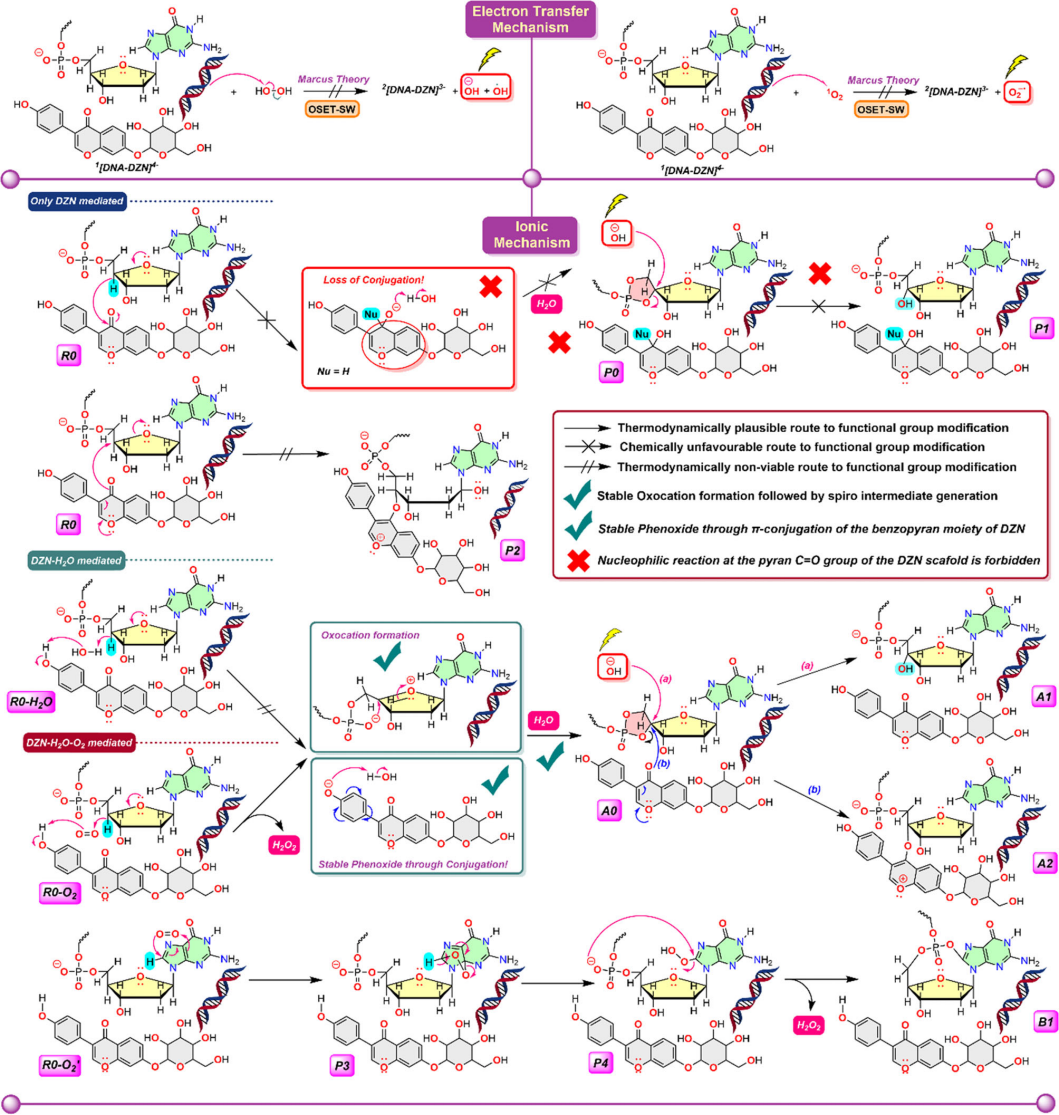
DFT-derived mechanistic pathways for DNA functional group modification at C4′ and C8. Two possible routes were examined: an outer-sphere electron-transfer or OSET (ionic) mechanism, and an ionic mechanism involving H_2_O, ^1^O_2_, or H_2_O_2_. The radical OSET pathway is thermodynamically inaccessible, whereas the ionic pathways involving DZN–H_2_O and DZN–H_2_O–O_2_ proceed via oxo-cation formation, phenoxide stabilization, and spiro-C4′ intermediates, ultimately leading to DNA fragmentation. Thermodynamically feasible routes are marked with ✓, while disfavored pathways are indicated with **×** for C4′ center activation. The route R0-O_2_′→P4 is taken from [53].

Based on previous reports [53–57], two DNA damage pathways were considered: a radical-mediated mechanism and an ionic mechanism (Scheme 1). For this purpose, we have adopted the C–H activation followed by functional group modification of the DNA deoxyribose sugar-phosphate back bone and Guanine base. All plausible mechanistic routes for the primary functional group modification (C–H to C–OH transformation) are summarized in Scheme 1. DFT results showed unfavorable pathways for such functional group modification under the influence of only DZN and DZN–H_2_O. The generation of singlet oxygen species in cancer cells [53, 58] and the absence of EPR signal in ROS detection experiment (Supplementary Fig. S2) enabled us to consider the role of ^1^O_2_ in the fragmentation pathway. Within the radical framework, Marcus’ theory applied under the outer-sphere single-electron transfer (OSET) model predicts that electron transfer between ¹ [DNA–DZN]^4−^ and singlet oxygen (^1^O_2_) is both kinetically and thermodynamically unfavorable (Δ*G*^‡^_OSET_ = 29.3 kcal mol⁻¹; Δ*G*^°^_OSET_ = 29.2 kcal mol⁻¹ at 37°C, Supplementary Tables S5 and S6). This agrees with the absence of an EPR signal (Supplementary Fig. S2), suggesting that the radical pathway leading to superoxide is not operative under these conditions. We therefore considered ionic mechanisms, involving three possible activation routes: (i) DZN-only, (ii) DZN–H_2_O, and (iii) DZN–H_2_O–O_2_. All proceed via oxo-cation formation following *sp^3^* C–H activation at the DNA C4′ position. However, in the DZN-only case, this protocol (R0→P0→P1) does not yield a stable oxo-cation. Instead, DFT calculations predict cleavage of the deoxyribose chain via the R0→P2 pathway (Scheme 1), which is strongly disfavored (Δ_r_*G* = 61.9 kcal mol⁻¹; *K*_eq_ ≈ 10^−44^), corresponding to a reaction essentially forbidden at 37°C.

**Scheme 2.**
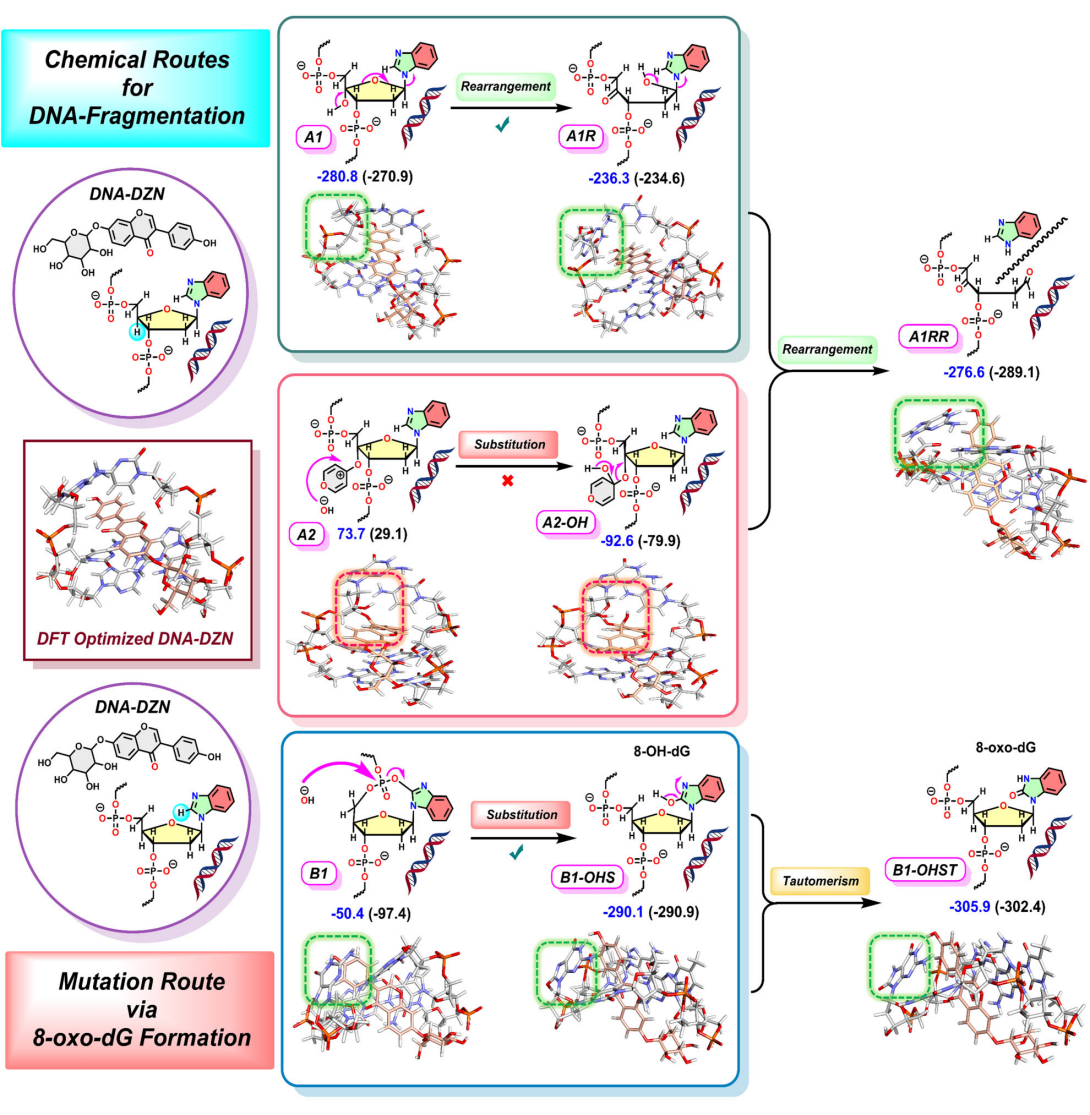
DFT-derived mechanistic pathways leading to DNA degradation. Two routes are observed: (i) deoxyribose fragmentation via A0→A1→A1RR, where successive rearrangements yield cleavage of the deoxyribose and detachment of the guanine base; and (ii) guanine mutation via B1→B1-OHS→B1-OHST, culminating in the formation of 8-oxo-dG. Green boxes are used to highlight the reaction center where external OH^−^ is the nucleophile and red boxes are used to highlight the reaction centers where the intercalated DZN is acting as the nucleophile.

Oxo-cation formation is feasible in both the DZN–H_2_O- and DZN–H_2_O–O_2_-mediated routes, each proceeding through an S_N_2-type reaction to yield the spiro intermediate A0. The oxo-cation is stabilized by resonance delocalization into the carbonyl group. In the DZN–H_2_O pathway (R0–H_2_O→A0), formation of A0 is uphill (Δ_r_*G* = 27.8 kcal mol⁻¹; *K*_eq_ ≈ 10^−20^), rendering this route thermodynamically unfavorable. In contrast, the O_2_-mediated pathway (R0–O_2_→A0) is strongly exergonic (Δ_r_*G* = –16.8 kcal mol⁻¹; *K*_eq_ ≈ 10^11^). In this case, singlet ^1^O_2_ abstracts a hydride from the deoxyribose sugar, forming a peroxide anion that subsequently removes a proton from the benzopyran –OH group of DZN. The resulting phenoxide, stabilized by π-conjugation, facilitates a spiro carbon center formation at the C4′ of A0. This pathway also generates hydrogen peroxide (H_2_O_2_), a known ROS [59]. Under the present conditions, however, H_2_O_2_ does not induce radical-based DNA fragmentation owing to the high OSET barrier (Δ*G*^‡^ = 38.8 kcal mol^−1^; Δ*G*° = 33.9 kcal mol^−1^ at 37°C). The phenoxide further abstracts protons from nearby water, increasing OH⁻ concentration and enabling the S_N_2-type transformation A0→A1. Of the subsequent routes, A0→A1 is thermodynamically accessible (Δ_r_*G* = –64.7 kcal mol⁻¹; *K*_eq_ ≈ 10^45^), whereas A0→A2 is not (Δ_r_*G* = 6.9 kcal mol⁻¹; *K*_eq_ ≈ 10^−5^). The A1 branch, therefore, constitutes the dominant functional group modification at the C4′ center, driving DNA fragmentation through subsequent rearrangements (Scheme 2).

In addition to C4′ modification, we examined a competitive C–H activation pathway mediated by ^1^O_2_ that targets the guanine nucleobase at C8. A similar initial activation sequence has been reported previously [53]. Because DZN is not directly involved in the early stages, the R0–O_2_′→P4 mechanism closely parallels earlier literature [53], where P3 corresponds to the endoperoxide intermediate and P4 denotes the 8-hydroperoxide species. Our DFT analysis focused on the subsequent step (P4→B1), which involves functional group modification at C8 accompanied by the release of H_2_O_2_. This transformation is strongly exergonic (Δ_r_*G* = –23.3 kcal mol⁻¹; *K*_eq_ ≈ 10^16^ at 37°C), rendering the reverse reaction inaccessible. The P4→B1 pathway ultimately produces guanine C8 modification/mutation, as illustrated in Scheme 2.

### Mechanistic insights into DZN-driven DNA degradation: a dual pathway model of ribose ring opening and guanine-to-8-oxoG mutation under redox-active conditions

As illustrated in Scheme 2, the chemical transformations proceed along two competing pathways: deoxyribose sugar fragmentation and guanine nucleobase mutation. Under conditions mimicking the cellular environment, DZN promotes hydroxide ion (OH⁻) release (Scheme 1), which initiates the downstream reactivity. Nucleophilic substitution at A0 predominantly yields A1, which is more stable than the alternative A2 formed by direct DZN attack (Schemes 1 and 2). A1 undergoes two successive rearrangements through the deoxyribose ring-opening channel to afford the low-lying product A1RR. Although the first rearrangement step (A1→A1R) requires a free energy input of 8.7 kcal mol^−1^ (ΔΔ_r_*G*), the overall pathway (R0–O_2_→A1RR) is highly exergonic (Δ_r_*G* = –69.1 kcal mol^−1^), with an equilibrium constant on the order of 10^48^, rendering it thermodynamically feasible. By contrast, the A2 branch proceeds via substitution at the DZN carbonyl carbon to give A2-OHS (Δ_r_*G* = –19.1 kcal mol⁻¹, *K*_eq_ ≈10^13^), which subsequently rearranges to converge at the same deoxyribose-cleavage product A1RR. In this final structure, hydroxyl groups of the ribose are converted into ketones, and the guanine base is fully detached from the DNA framework, effectively cleaving the backbone.

In the alternative mutation pathway, nucleophilic substitution occurs at the P–O–P bridge of B1, yielding the highly stable intermediate B1–OHS (Δ_r_*G* = –69.5 kcal mol^−1^, *K*_eq_ ≈ 10^49^), corresponding to 8-hydroxy-2′-deoxyguanosine (8-OHdG). This intermediate undergoes an exergonic tautomerization to form B1–OHST (Δ_r_*G* = –72.3 kcal mol^−1^, *K*_eq_ ≈ 10^50^), identified as 8-oxo-dG [53]. Thus, the B1→8-oxo-dG route represents a thermodynamically favorable mutational pathway that operates in parallel with the deoxyribose-cleavage mechanism under redox-active conditions in the presence of DZN and associated oxidants. Our calculation illustrates that both pathways are strongly exergonic and thermodynamically feasible under redox-active conditions.

### Comparative intercalation dynamics of daidzin and doxorubicin: structural disassembly of the DNA helix correlated with π–π interactions and helical conformational decay

**Figure 3.**
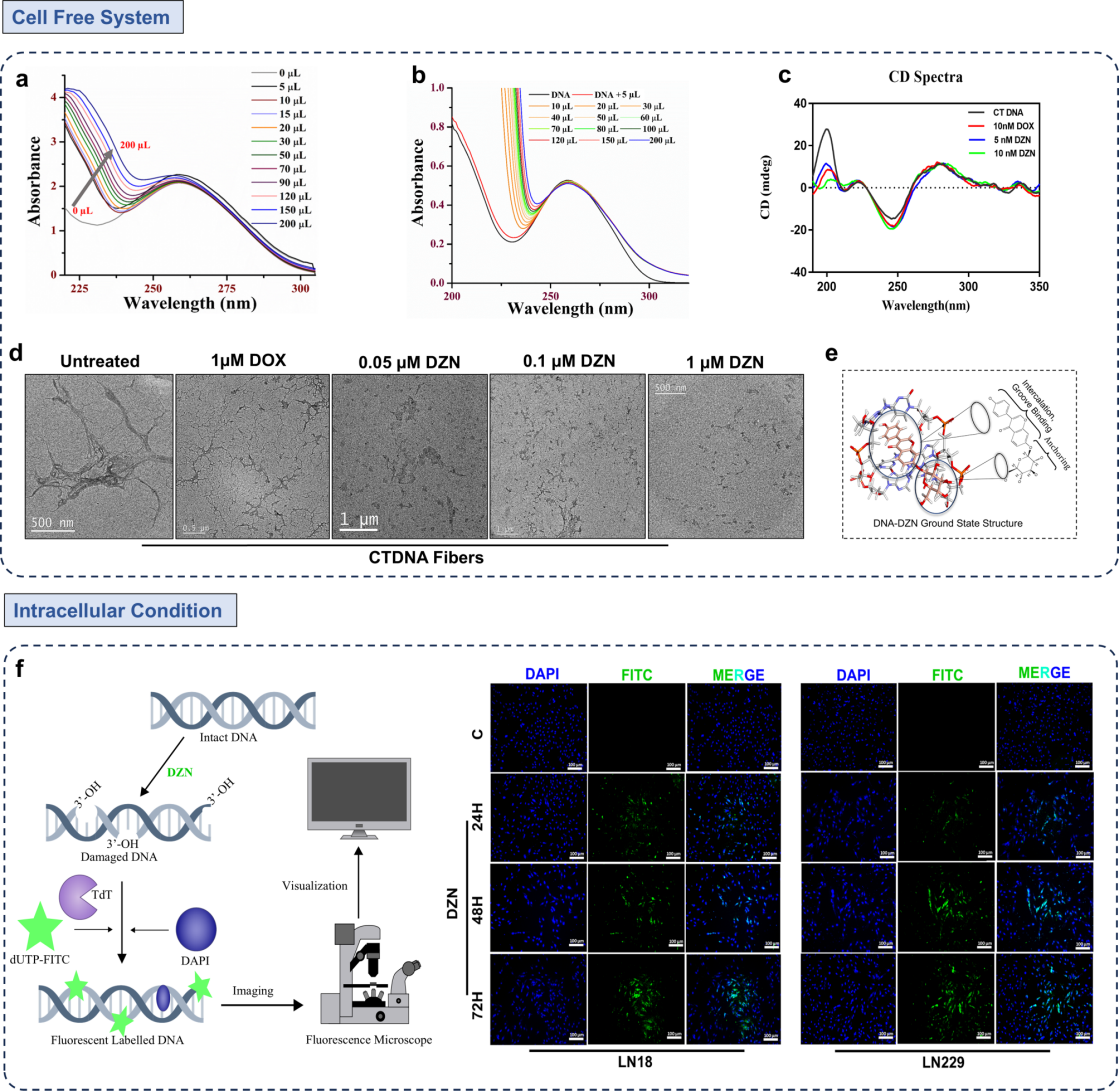
Structural and spectroscopic characterization of DZN-induced DNA fragmentation and DNA interaction analysis. (**a** and **b**) UV–Vis spectroscopy analysis of DZN and DOX at varying concentrations. Spectral shifts in absorbance provide insights into electronic transitions and molecular interactions, with distinct absorption peaks corresponding to ligand–DNA binding dynamics. Comparative analysis between DZN and DOX highlights differences in their DNA-binding affinity. (**c**) Circular dichroism (CD) spectroscopy of DZN and DOX at varying concentrations. CD spectra reveal alterations in DNA secondary structure upon ligand interaction, with changes in ellipticity suggesting conformational distortions induced by intercalation or groove binding. (**d**) Transmission electron microscopy (TEM) images of DNA fragmentation in the DZN–DNA complex. TEM analysis reveals morphological changes associated with DNA cleavage, confirming structural disintegration upon DZN binding. Representative micrographs depict DNA strand breakage and aggregation patterns, indicative of severe DNA destabilization. (**e**) DNA–DZN interaction profile. A comprehensive spectroscopic evaluation detailing the binding kinetics, affinity, and structural modifications in the DNA–DZN complex, reinforcing the molecular basis of DZN-induced DNA perturbation. (**f**) TUNEL assay detecting DNA fragmentation as a marker of apoptosis. LN18 and LN229 cells were treated with IC_50_ concentrations of DZN for 24, 48, and 72 h, followed by fluorescence microscopy imaging. An increase in green fluorescence (dUTP-FITC incorporation at 3′-OH termini of fragmented DNA) confirms apoptotic DNA damage. A pictorial representation of the TUNEL assay workflow is also provided.

The UV–Vis absorption spectra of DNA in the presence of increasing concentrations of DZN (Fig. 3a) and DOX (Fig. 3b) revealed distinct interaction patterns indicative of classical intercalative binding modes. For DZN, a pronounced hyperchromic effect was observed with increasing ligand concentration, characterized by a significant increase in absorbance around 225 nm, accompanied by a slight red shift [60, 61]. This suggests substantial disruption of the base stacking interaction within the DNA double helix [61, 62]. In contrast, DOX exhibited a more moderate hyperchromism with a gradual increase in absorbance and a subtle red shift [60, 62]. This indicates intercalation with less pronounced base stacking disruption [58, 63] consistent with the understanding that spectral changes in UV-Vis are associated with the conformation of DNA [62, 64]. These spectral changes confirm that both compounds intercalate into DNA, but DZN induces stronger perturbations in the DNA structure [65, 66], likely due to its distinct molecular interactions as previously observed in our *in silico* studies, which may influence its biological activity [61, 64]. Such UV–Vis spectral analyses provide valuable insights into the binding affinity and mode of interaction of small molecules e.g. DZN with DNA, supporting their potential therapeutic applications.

The CD spectra (Fig. 3c) of native CTDNA exhibit three characteristic bands: a positive ellipticity near ∼275 nm, a negative ellipticity near ∼245 nm, associated with the right-handed helicity and exciton coupling of base pairs that reflect the overall chiral conformation of the DNA helix [67, 68] and a positive ellipticity near ∼200–210 nm arising from asymmetric sugar-phosphate backbone geometry, indicating backbone conformation and helical twist [67]. Upon treatment with DZN and DOX, the ∼275 nm positive band remained largely unchanged in intensity, suggesting minimal impact on overall base stacking. In contrast, the negative band near ∼245 nm became more intense (greater negative ellipticity) for both DZN and DOX, indicating increased perturbation of DNA helicity and local conformational distortion (Fig. 3c). Notably, the positive ellipticity near ∼200–210 nm exhibited a significant decrease with DZN (5 and 10 nM) and DOX treatment, compared to free DNA, suggesting considerable alterations in the DNA backbone conformation and helical twist. These findings suggest that while both DZN and DOX intercalate into DNA and perturb helicity, DZN’s interaction induces pronounced backbone destabilization and helical disruption at lower concentration similar to that observed in UV–Vis spectra (Fig 3a), which is consistent with the severe fragmentation observed in low-dose TEM images (Fig. 3d).

To further investigate the DNA-damaging potential of DZN compared to DOX, a standard DNA intercalator, we employed low-dose TEM using CT-DNA alone and in the presence of DOX (1 µM) and varying concentrations of DZN (0.05, 0.1, and 1 µM) (Fig 3d). Untreated CTDNA exhibited long, continuous, and well-structured DNA fibers, indicative of an intact double-helical structure. CTDNA displayed extensive fragmentation when treated with 1 µM DOX, as expected from its established intercalative mode of action. Remarkably, DZN at just 0.05 µM induced complete DNA fragmentation comparable to 1 µM DOX, demonstrating its ability to exert a DNA-disrupting effect at a much lower concentration than DOX. At 0.1 and 1 µM DZN, the fragmented DNA structures persisted, suggesting a dose-dependent effect in which higher DZN concentrations maintain or even enhance DNA cleavage. The collective findings from TEM, UV–Vis, and CD spectroscopy suggest a possible binding mechanism for DZN where the isoflavone ring of DZN engages in π–π interactions with N_2_ bases of DNA, similar to the intercalation mechanism observed with DOX. The sugar moiety of DZN may serve as an anchor, stabilizing the DNA–DZN complex and potentially enhancing its effect on DNA, similar to anthracycline intercalators [69] (Fig. 3e). This enhanced stabilization may account for the greater DNA fragmentation observed at lower concentrations of DZN compared to DOX. Moreover, TUNEL assay results corroborated these findings, demonstrating increased green fluorescence in LN18 and LN229 cells due to dUTP–FITC incorporation at 3′-OH termini of fragmented DNA; however, no fluorescence was observed in SVGP12 upon DZN treatment (Fig. 3f and Supplementary Fig. S4). The presence of DNA fragmentation, a hallmark of apoptotic cell death, strongly supports that DZN induces glioma cell apoptosis through DNA damage and subsequent activation of the apoptotic cascade (Fig. 3f).

### Daidzin exhibits potent anti-glioma activity by inducing cell cycle arrest, inhibiting migration, and promoting apoptosis

**Figure 4.**
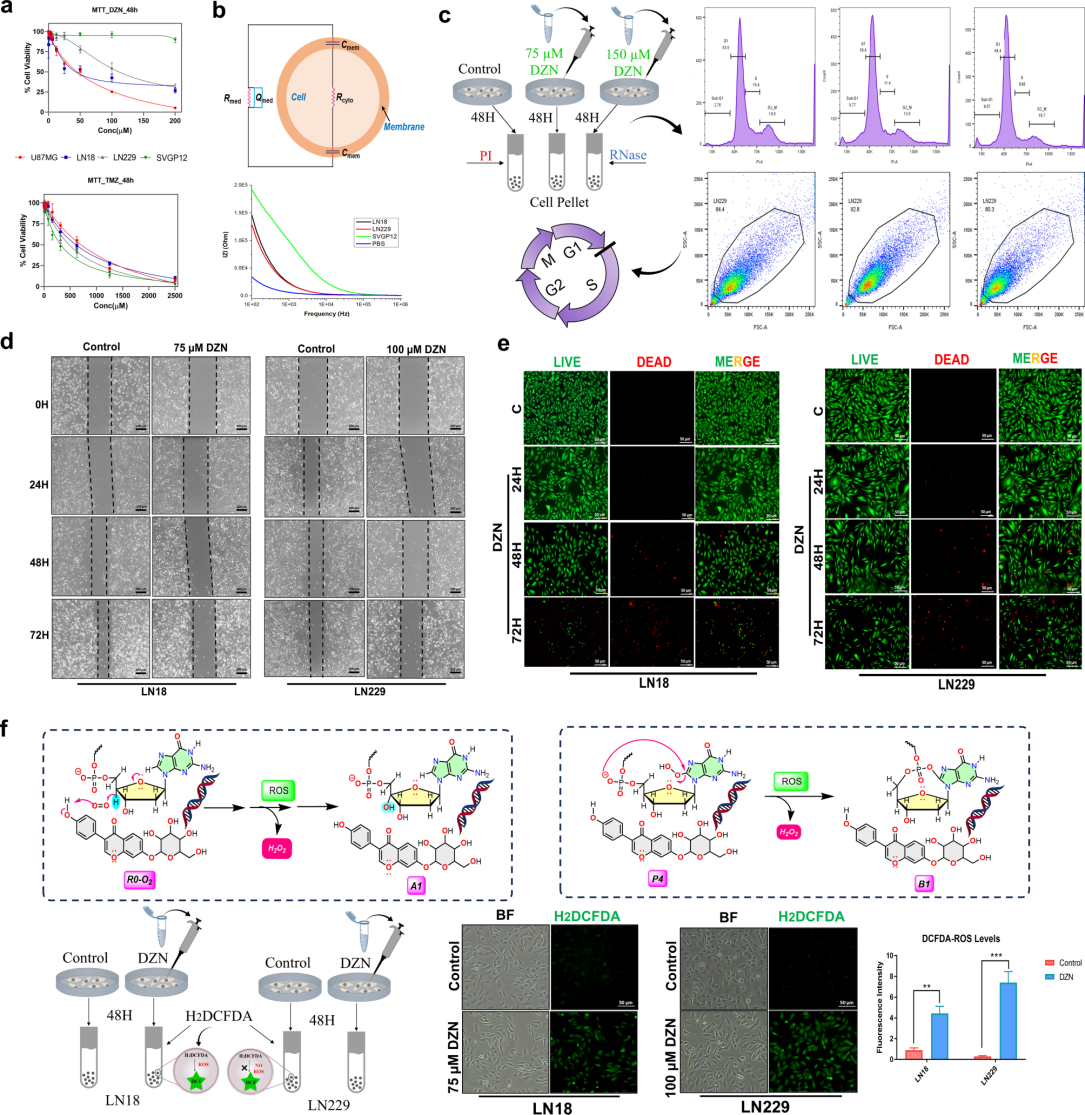
DZN exhibits potent anti-glioma activity by inducing cell cycle arrest, inhibiting migration, and promoting apoptosis. (**a**) MTT cytotoxicity assay for DZN, TMZ in LN18, LN229, U87MG, and noncancerous glial cell line SVGP12. Cells were treated with a starting concentration of 200 µM DZN, serially diluted 10-fold, and incubated to assess cell viability. The starting concentration of TMZ was 2500 µM, serially diluted 10-fold, demonstrating that TMZ required substantially higher concentrations to achieve comparable glioma cell inhibition, highlighting DZN’s superior potency. (**b**) Schematic representation of the electrical equivalent circuit of a biological cell. Impedance spectra showing the variation in impedance magnitude with frequency (Hz) for the different cell populations (LN18, LN229, SVGP12). Measurements were performed over a frequency range of 40 Hz –100 MHz using a sinusoidal excitation voltage of 15 mV. A pronounced change in impedance magnitude is observed between 100 Hz and 10 kHz. Representative figure is presented from three independent experiment. (**c**) Cell cycle analysis using flow cytometry (FACS) in LN229 cells. Cells were treated with a low dose (75 µM) and high dose (150 µM) of DZN, and the G1-phase cell cycle arrest was quantified. A schematic overview of the experimental workflow is presented, along with a graphical representation of the increase in G1-phase population and sub-G1 apoptotic fractions. (**d**) Migration assay assessing the inhibitory effects of DZN on glioma cell motility. LN18 and LN229 cells were treated with IC_50_ concentrations of DZN, and migration was monitored over 72 h, showing significant suppression of glioma cell movement. (**e**) Live/Dead assay demonstrating DZN-induced apoptosis in a time-dependent manner. LN18 and LN229 cells were treated with IC_50_ concentrations of DZN and captured at 24, 48, and 72 h. Red fluorescence (PI) indicates dead cells, while green fluorescence (Calcein AM) represents viable cells. An increase in PI-positive cells and a decline in live cell fluorescence over time confirms DZN’s cytotoxic effects. (**f**) Mechanistic route derived from quantum-chemical analysis of the DNA–DZN adduct showing photo independent, ionic ROS generation: the singlet-oxygen–assisted R0–O₂→A₀→A₁ (deoxy ribose activation/fragmentation) and P4→B1 (guanine C8 oxidation) routes produce H_2_O_2_, rationalizing the DCFDA signal increase observed in glioma cells. Representative DCFDA fluorescence micrographs of LN18 and LN229 cells following vehicle and DZN exposure. Robust green fluorescence is evident in DZN-treated LN18 and LN229 cells, consistent with increased ROS burden.

To evaluate the therapeutic potential of DZN against glioma, we determined its half-maximal inhibitory concentration (IC_50_) across various glial cell lines. DZN exhibits significantly greater cytotoxicity against glioma cells compared to the standard chemotherapeutic agent TMZ as represented by corresponding IC_50_ values. The IC_50_ values of DZN in LN18, LN229, and U87MG glioma cell lines were ≈62.111, ≈118.85, and ≈47.917 µM (Fig. 4a), respectively, whereas TMZ required substantially higher concentrations of ≈606.20, ≈471.80, and ≈716.67 µM (Fig. 4a), respectively, to achieve comparable inhibition. This highlights superior efficacy of our compound in suppressing glioma cell proliferation. Notably, DZN exhibited no detectable toxicity in noncancerous glial cells (SVGP12) even at concentrations up to 200 µM (Fig. 4a), suggesting a strong tumor-selective cytotoxic effect. However, IC_50_ value of TMZ against SVGP12 was found to be ≈312 µM, comparable to those identified for different glioma cell lines, validating its cytotoxic effect in all types of cells. This remarkable difference underscores DZN’s superior efficacy in suppressing glioma cell proliferation, suggesting its significance as a more potent alternative to standard anti-glioma drug, TMZ. To further dissect the mechanism underlying DZN’s cytotoxicity, flow cytometry (FACS) analysis was conducted to assess its impact on cell cycle progression. The results demonstrated that DZN treatment led to a pronounced G1 cell cycle arrest, thereby preventing glioma cells from transitioning into the S phase. Additionally, a time-dependent increase in the sub-G1 population was observed, indicating the accumulation of apoptotic cells (Fig. 4c). These findings suggest that DZN inhibits glioma progression by disrupting cell cycle regulation and subsequently inducing apoptosis. In addition to its anti-proliferative effects, DZN significantly impaired the migratory potential of LN18 and LN229 glioma cells in a time-dependent manner (Fig. 4d). This inhibition of cell migration suggests that DZN may also interfere with glioma invasiveness, a critical factor in tumor progression and metastasis. To further confirm the induction of apoptosis, a live/dead assay was performed. DZN treatment increased PI red fluorescence and concomitantly reduced calcein AM green fluorescence in a time-dependent manner, indicating a significant shift from viable to non-viable cells (Fig. 4e).

DZN markedly elevated intracellular ROS in LN18 and LN229 cells, as evidenced by increased DCFDA-derived green fluorescence in fluorescence micrographs (Fig. 4f). This observation is mechanistically concordant with our DFT predictions: the ^1^O_2_-assisted ionic pathways (R0–O₂→A₀→A₁ and P4→B1) generate H_2_O_2_ as a by-product, plausibly increasing the intracellular peroxide burden and, consequently, the DCFDA signal. Electrochemical impedance measurements further support a basis for selectivity. The resistance and capacitance values for SVGP12, LN18 and LN229 cells were determined using impedance-based spectral analysis and then fitted into a Bode plot using an equivalent circuit model in the “EIS Spectrum Analyser” opensource software to extract the cell membrane resistance and capacitance (Fig. 4b). The extracted parameters were then used to derive the overall impedance values using the equation: overall impedance = (*R*_med_ || *Q*_med_) – (*C*_mem_ || (*R*_cyto_* – C*_mem_)), where *R*_med_ is the medium resistance, while *Q*_med_ represents the constant-phase element (CPE) (Supplementary Table S8). The CPE is a nonideal capacitor with a phase angle between 0° to 90° and is expressed as 1/ [*Q(jw)^n^*], where Q represents the CPE, while n represents the deviation from ideal capacitance; *R*_cyto_ represents cytoplasmic resistance, and *C*_mem_ represents membrane capacitance [70].The analysis mainly focused on the changes in *R*_cyto_ and *C*_mem_, as *R*_med_ is determined by the conductivity of the surrounding medium in which the cells are suspended and therefore have a negligible effect on the overall impedance. As evident from the Bode plot (Fig. 4b), SVGP12 cells exhibited significantly higher overall impedance (corresponding to *R*_med_: 1.5E05 Ohm, *C*_mem_:1.4 nF) than LN18 (corresponding to *R*_med_: 1E06 Ohm, *C*_mem_:6.79 nF) and LN229 (corresponding to *R*_med_: 1E06 Ohm, *C*_mem_:6.84 nF), consistent with greater membrane integrity and lower passive permeability (Supplementary Table S8). In contrast, the more fluidic membranes of glioma cells are expected to facilitate passive diffusion of small molecules such as DZN, enhancing intracellular accumulation and amplifying oxidative injury at low extracellular concentrations (Fig. 4b). Together, enhanced ROS production and preferential uptake provide a coherent explanation for DZN’s tumor-selective cytotoxicity.

Collectively, these findings highlight DZN’s potent anti-glioma properties, characterized by its ability to arrest cell cycle progression, inhibit migration, and trigger apoptosis via DNA fragmentation. Given its significantly lower IC₅₀ compared to TMZ, DZN emerges as a highly promising candidate for glioma therapy, deserving further investigation into its molecular targets and potential clinical applications.

### Daidzin induces extensive DNA damage in glioma cells by promoting p-ATM-mediated double-strand breaks and 8-OHdG-associated oxidative stress

**Figure 5.**
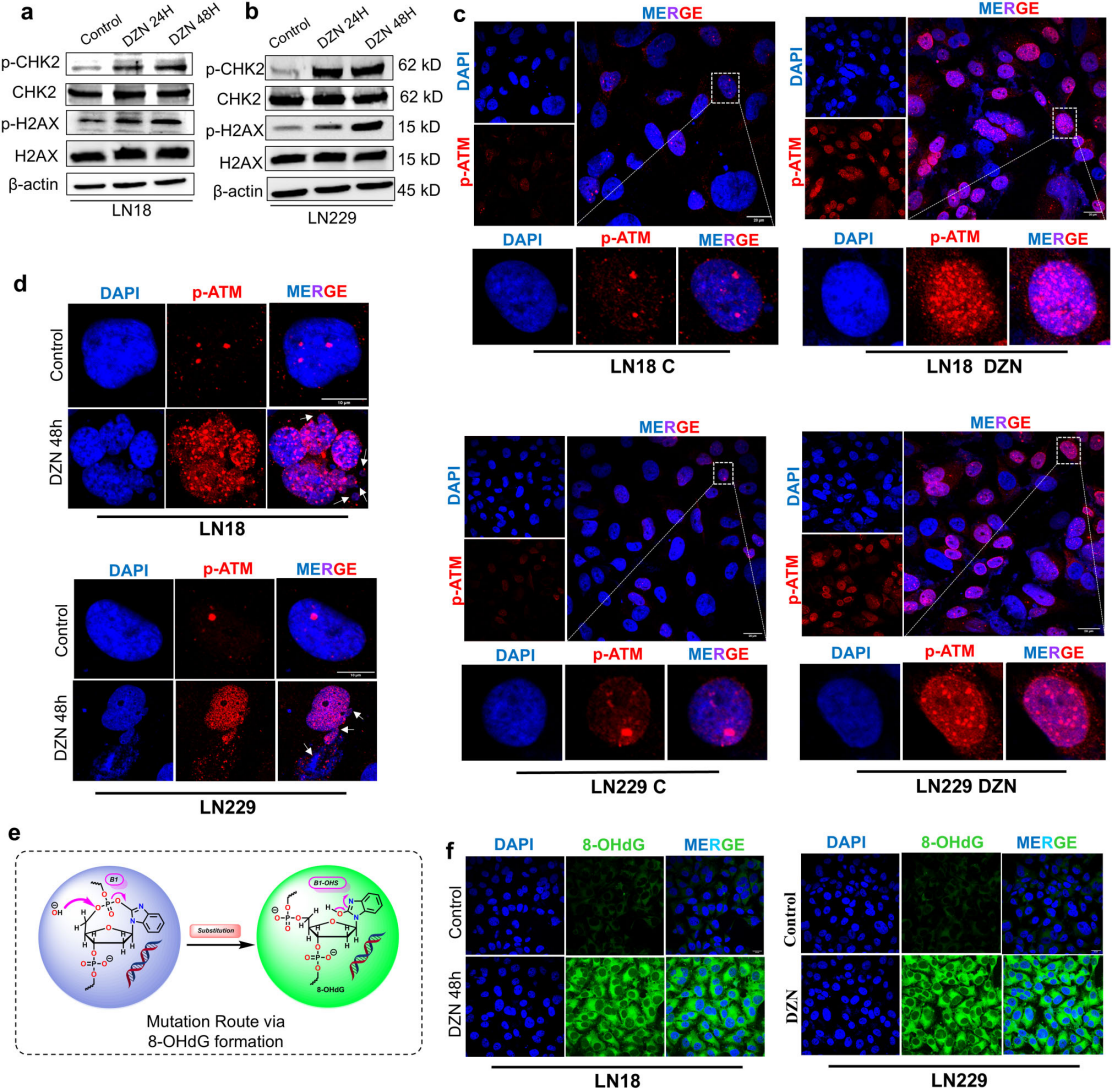
DZN induces extensive DNA damage in glioma cells by promoting p-ATM-mediated DSBs and 8-OHdG-associated oxidative stress. Western blot analysis of DNA damage response markers p-CHK2, CHK2, p-H2AX, and H2AX in (**a**)LN18, (**b**) LN229 cells following DZN treatment. The cells were treated with low-dose (50 µM in LN18, 75 µM in LN229) and high-dose (100 µM in LN18, 150 µM in LN229) DZN, and protein levels were assessed compared to untreated controls. β-Actin was used as a loading control. (**c**) Immunofluorescence cytometry (IFC) analysis of phosphorylated ataxia-telangiectasia mutated protein (p-ATM) foci in LN18 and LN229 cells following DZN treatment. (**d**) Assessment of nuclear leakage following DZN treatment in LN18 and LN229 cells. (**e**) Schematic representation of B1**→** B1-OHS route, producing 8-OHdG (**f**) 8-hydroxy-2′-deoxyguanosine (8-OHdG) immunofluorescence assay in glioma cells treated with DZN.

To elucidate the mechanistic basis of DZN’s cytotoxicity in glioma cells, we assessed its impact on key apoptotic regulators in LN18 and LN229 cells. Western blot analysis revealed a significant shift in the balance between pro-apoptotic and anti-apoptotic proteins, favoring apoptosis. DZN treatment upregulated the expression of Bax and Bak, critical pro-apoptotic markers, while simultaneously downregulating Bcl-xL, XIAP (Supplementary Fig. S4), key anti-apoptotic proteins that maintain cell survival. This indicates that DZN disrupts mitochondrial integrity, thereby promoting apoptotic signaling cascades in glioma cells. Beyond its apoptotic effects, DZN induced extensive DNA damage, particularly DSBs, as evidenced by the overexpression of phosphorylated checkpoint kinase 2 (p-CHK2) and phosphorylated histone H2AX (p-H2AX) in western blot analyses (Fig. 5a and b), while the total protein levels of CHK2 and H2AX (CHK2 and H2AX) remained unchanged. These key DNA damage response (DDR) proteins are essential mediators of genomic stability, and their heightened expression suggests that DZN-induced genotoxic stress leads to severe DNA fragmentation and checkpoint activation. Further validation of DZN’s DSB-inducing capability was obtained from immunofluorescence cytometry (IFC) analysis, where a robust increase in phosphorylated ataxia-telangiectasia mutated protein (p-ATM) foci was observed in both LN18 and LN229 cells (Fig. 5c and Supplementary Fig. S5). ATM kinase serves as a master regulator of the DNA damage response, and the presence of p-ATM foci strongly correlates with the formation of DSBs, reinforcing the notion that DZN exerts severe genotoxic effects. In addition to DNA strand breaks (Fig. 3d and f), DZN treatment induced nuclear leakage in both the glioma cells, a phenomenon indicative of compromised nuclear membrane integrity and severe chromatin disorganization (Fig. 5d). The nuclear destabilization further underscores DZN’s ability to disrupt cellular homeostasis, potentially pushing glioma cells toward irreversible damage and apoptosis. FACS analysis demonstrated a marked increase in G1-phase arrested cells, suggesting that DZN inhibits glioma proliferation by preventing the transition from G1 to S phase. This is a direct consequence of ATM/p-CHK2 pathway activation, which enforces a G1 checkpoint upon sensing DSBs, thereby preventing cells with damaged DNA from entering the S phase.

Our *in silico* analysis revealed DZN can lead to formation of 8-OHdG and 8-oxo-dG via B1→ B1-OHS route, consistently with our *in vitro* findings where DZN significantly upregulated the expression of 8-hydroxy-2′-deoxyguanosine (8-OHdG), a well-established biomarker of oxidative DNA damage in both LN18, and LN229 cells (Fig. 5e and f). This implicates ROS generation as a key contributor to DZN-induced cytotoxicity in glioma.

Together, these findings establish DZN as a potent glioma-targeting compound that exerts its effects through multiple converging mechanisms: mitochondrial apoptosis activation, ROS-induced genomic instability, and cell cycle disruption via DSB-mediated G1 cell cycle arrest. The combined evidence from western blot, IFC analysis, FACS studies, and *in silico* predictions strongly supports DZN’s therapeutic potential, demanding further investigation into its molecular targets and translational viability in glioma treatment.

### *In vivo* antitumor efficacy and systemic safety of daidzin in a C6 glioma rat model

**Figure 6.**
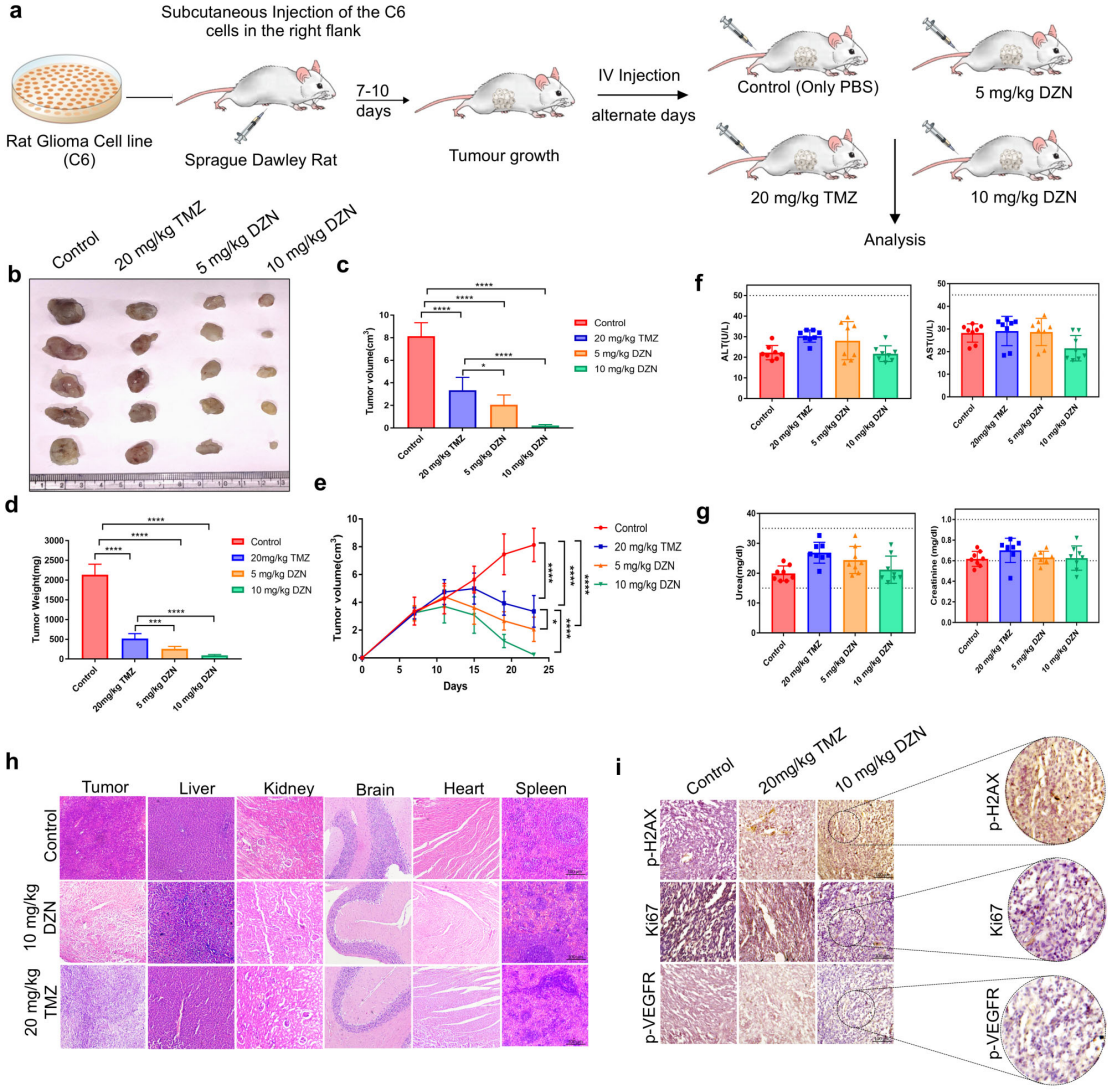
DZN suppresses C6 glioma growth and is well tolerated in Sprague–Dawley rats. (**a**) Study scheme: 6-week-old Sprague–Dawley rats were implanted subcutaneously with C6 glioma cells. Intravenous dosing (tail vein) commenced on day 7 at ∼300 mm³ tumor volume. Groups (*n* = 8 each): PBS (control), TMZ (20 mg·kg⁻¹), DZN (5 mg·kg⁻¹), and DZN (10 mg·kg⁻¹). (**b**) Representative images of tumors from treatment groups receiving PBS (control), TMZ (20 mg·kg⁻¹), DZN (5 mg·kg⁻¹), and DZN (10 mg·kg⁻¹). (**c**) Final volumes at day 23: control ∼8.12 ± 1.12 cm³, TMZ ∼3.3 ± 1.06 cm³, DZN 5 mg·kg⁻¹ ∼2 ± 0.82 cm³, and DZN 10 mg·kg⁻¹ ∼0.22 ± 0.08 cm³ (mean ± SD; unpaired two-tailed *t*-test; statistics indicated in plots). (**d**) Tumor weights at necropsy (mean ± SD): control 2138.52 ± 247.18 mg; TMZ 519.2 ± 116.58 mg; DZN 5 mg·kg⁻¹ 258.24 ± 59.01 mg; DZN 10 mg·kg⁻¹ 90.36 ± 20.72 mg; significance versus control and TMZ as indicated. (**e**) Longitudinal tumor trajectories (measured every 4 days); DZN 10 mg·kg⁻¹ shows tumor regression beginning from ∼day 12. (**f**) Hepatic chemistry (e.g. AST/ALT) remained within physiological ranges across groups. (**g**) Renal and metabolic panels (urea, creatinine) were unaltered, indicating absence of nephrotoxicity. (**h**) H&E histopathology of heart, spleen, brain, kidney, and liver revealed intact cytoarchitecture without inflammatory, degenerative, or necrotic lesions. However, H&E histopathology of tumors showed clear dose-dependent reduction in tumor cell density accompanied by an expansion of intercellular spaces in the treated groups relative to the untreated controls. (**i**) Tumor immunohistochemistry: increased p-H2AX in DZN-treated tumors (DNA damage), with concomitant reductions in Ki-67 (proliferation) and p-VEGFR (angiogenic signaling). Scale bars and magnifications as indicated. The values are represented as mean ± SD. *P < 0.05, **P < 0.01, ***P < 0.001, ***P < 0.0001.

In a subcutaneous C6 glioma rat model, systemic administration of DZN dramatically suppressed tumor growth compared to both the untreated control and the standard chemotherapy TMZ (Fig. 6a and Supplementary Fig. S6). Treatment was initiated when tumors reached ∼3 cm³ (Day 0 of therapy), and tumor volumes were monitored every 4 days thereafter. By the study endpoint (Day 23), control tumors had grown aggressively to approximately 8.12 ± 1.12 cm³, whereas all treated groups showed significantly lower tumor burdens (Fig. 6c and e). Notably, 20 mg/kg TMZ (the standard glioma chemotherapeutic) limited tumor expansion to ∼3.3 ± 1.06 cm³. Low-dose DZN at 5 mg/kg achieved a endpoint volume of ∼2 ± 0.82 cm³ (>70% reduction versus control, Fig. 6c and e) and high-dose DZN at 10 mg/kg almost completely halted tumor progression, final tumors were only ∼0.22 ± 0.08 cm³, reflecting a significant reduction in volume relative to controls and TMZ group (Fig. 6c and e). In fact, the 10 mg/kg DZN group showed evidence of tumor regression, as their average tumor size peaked around Day 11 and then declined thereafter (Fig. 6e), an outcome not observed in control or TMZ-treated animals. These volumetric results were corroborated by tumor weight measurements at necropsy. Mean tumor weights in the DZN 10 mg/kg group were just 90.36 ± 20.72 mg, dramatically lower than in controls (2138.52 ± 247.18 mg; >90% reduction) and significantly below the 519.2 ± 116.58 mg in the TMZ group (Fig. 6d). Even 5 mg/kg DZN yielded tumors of 258.24 ± 59.01 mg, indicating substantial regression compared to 20 mg/kg TMZ and control (all values mean ± SD for *n* = 8 rats/group) (Fig. 6d). Collectively, these results demonstrate a potent tumor-suppressive effect of DZN, with the high dose producing marked tumor regression surpassing the efficacy of standard TMZ.

Importantly, DZN treatment exhibited no apparent systemic toxicity at either 5 or 10 mg/kg, as evidenced by comprehensive clinical chemistry, hematology, and histopathological analyses. All treatment groups tolerated the IV injections well, with no observed adverse behaviors or weight loss. Hematological parameters remained within normal physiological ranges across control and treated rats (Fig. 6f and g). There were no significant differences in hemoglobin levels, total and differential leukocyte counts (neutrophils, lymphocytes, eosinophils, monocytes, and basophils), erythrocyte counts, hematocrit (PCV), or platelet counts between DZN-treated and control animals (Supplementary Table S9). Red blood cell indices (MCV, MCH, and MCHC) were likewise unaltered, indicating no myelosuppressive or hematotoxic effects of DZN (Supplementary Table S9). Similarly, serum biochemistry profiling revealed that DZN did not induce hepatic or renal toxicity. Liver enzymes aspartate aminotransferase (AST) and alanine aminotransferase (ALT) remained at baseline levels in DZN-treated rats, comparable to controls, indicating no hepatocellular damage (Fig. 6f). Bilirubin (total, direct, and indirect), as well as total protein and albumin concentrations, showed no aberrations, further supporting normal liver function (Supplementary Table S9). Renal function markers including blood urea and creatinine were also normal in all groups (Fig. 6g), with no elevation in DZN-treated rats, signifying absence of nephrotoxicity. Blood uric acid was unchanged, and electrolyte levels (sodium, potassium, and chloride) and calcium were all within normal limits across groups (Supplementary Table S9). Consistently, gross necropsy of major organs and histological examination (H&E staining) revealed no pathological lesions or tissue damage attributable to DZN (or TMZ) treatment (Fig. 6h and Supplementary Fig. S7). Figure 6h shows representative H&E sections of heart, spleen, brain, kidney, and liver from DZN-treated animals, which were indistinguishable from control tissues with intact cytoarchitecture and no signs of inflammation, degeneration, or necrosis. These findings confirm that DZN is well-tolerated at therapeutically effective doses, aligning with reports of minimal toxicity in preclinical studies of DZN (Fig. 6h).

While DZN caused no harm to normal organs, it induced pronounced cellular and molecular changes within the tumors consistent with an anti-cancer mechanism of action. Histological H&E staining of excised tumors revealed that control tumors were highly cellular and vascular with frequent mitotic figures, whereas DZN-treated tumors showed large areas of cell death/necrosis and reduced cellularity (Fig. 6h). Additionally, it demonstrated a clear dose-dependent reduction in tumor cell density accompanied by an expansion of intercellular spaces in the treated groups relative to the untreated controls (Fig. 6h). To further characterize these effects, we performed immunohistochemical (IHC) analysis of key tumor biomarkers on the harvested tumor sections (Fig. 6i). DNA damage marker p-H2AX (phosphorylated H2AX) was markedly elevated in DZN-treated tumors relative to controls. Tumors from rats receiving 10 mg/kg DZN exhibited intense nuclear p-H2AX staining in many tumor cells, indicating widespread DNA DSBs induced by the treatment. In contrast, control tumors showed only baseline p-H2AX positivity, reflecting minimal spontaneous DNA damage (Fig. 6i). The increase in p-H2AX level in DZN groups suggests enhanced DNA damage and tumor cell apoptosis triggered by DZN. Consistent with this, the proliferative index of the tumors was significantly diminished by DZN treatment. IHC for Ki-67, a nuclear antigen expressed during cell division, revealed a high percentage of Ki-67⁺ proliferating cells in control tumors (Fig. 6i). This was dramatically reduced in DZN-treated tumors, especially at 10 mg/kg, indicating that DZN effectively suppressed tumor cell proliferation (qualitatively fewer Ki-67 positive nuclei) (Fig. 6i). Likewise, an anti-angiogenic effect of DZN was evidenced by reduced staining for phosphorylated VEGF Receptor (p-VEGFR) in the treated tumors. Control tumors demonstrated strong p-VEGFR immunoreactivity associated with abundant blood vessel structures, whereas DZN-treated tumors showed greatly diminished p-VEGFR signals, correlating with likely decreased tumor vascularization (Fig. 6i). These IHC findings support that DZN treatment induces DNA damage in tumor cells while inhibiting their proliferation and angiogenesis, ultimately leading to tumor regression. Such multi-faceted antitumor activity of DZN is in line with emerging reports that DZN (daidzin) can promote oxidative DNA damage/apoptosis and inhibit cancer cell proliferation and angiogenic pathways. Importantly, the standard TMZ treatment exhibited a partial effect on these markers (Fig. 6i). TMZ is known to cause DNA damage and growth arrest in gliomas, but in our study the high-dose DZN elicited a markedly stronger response, as reflected by higher p-H2AX and lower Ki-67/p-VEGFR levels than achieved by TMZ. Overall, the *in vivo* tumor regression data combined with the absence of systemic toxicity underscore the potent therapeutic index of DZN in this glioma model. These results position DZN as a highly effective anticancer agent that can surpass the efficacy of standard chemotherapy (TMZ) while being well-tolerated, warranting further investigation into its mechanism and potential clinical translation.

## Discussion

This study leverages molecular dynamics simulations, quantum-chemical analyses, complemented with multi-modal *in vitro* and *in vivo* experiments to elucidate how the screened isoflavone, DZN, perturbs DNA structure and function, revealing a distinct intercalation geometry and dual chemical mechanism underlying its potent anti-glioma activity.

Our MD simulation analysis (Fig. 1) suggests that DZN consistently produced a ligand-specific deformation profile that is mechanistically distinct from the classical anthracycline motif, and these structural signatures aligned with pronounced biochemical and cellular consequences in glioma models.

At the level of local base-pair geometry, DRN induced the expected intercalative pattern with flattening of base pairs via propeller relaxation, increased opening, and modest negative stagger, together with pronounced terminal buckling consistent with wedge-like separation around the intercalation site (Fig. 1e and Supplementary Table S2). However, DZN produced a heterogeneous but sharper distortion with strong terminal curvature, a large positive propeller at the ends, substantial shear, and more negative stretch with modest opening (Fig 1e and Supplementary Table S2). These features indicate lateral base-pair sliding and curvature with limited in-pair unzipping, i.e. a packing-dominated deformation rather than the classical “unzipping” observed with DRN. Average base-step parameters reinforced this distinction. Both ligands widened the intercalation gap (rise ↑), but DRN caused net unwinding and positive slide with negative shift, whereas DZN drove net overwinding (particularly at terminal d(CG)/d(GC) steps), positive shift, smaller positive slide, and a pronounced negative terminal tilt (Fig. 1f and Supplementary Table S3). Helical descriptors reached the same conclusion where DRN widened and unwound the helix, flattened inclination, and translated the helical axis (*X/Y* reversal); whereas DZN widened but overwound the helix with minimal axis flattening and comparatively small lateral axis translation (Fig. 1g and Supplementary Table S4). Ligand-centered radial distributions further showed that DRN yields a more localized and rigid distortion, while DZN elicits a distributed, elastically accommodated rearrangement along neighboring steps (Fig. 1d and Supplementary Table S1). Together, these data indicate that DZN intercalates with a distinct geometric signature with widening plus overwinding than DRN.

In accordance with the MD simulation results, UV–Vis spectroscopy data revealed marked hyperchromism with a red shift for both ligands, consistent with intercalative stacking (Fig. 3a and b). The stronger hyperchromic response to DZN indicates greater disruption of base stacking and/or a larger population of perturbed states at comparable concentrations. Circular dichroism (CD) detected robust changes in the helicity-sensitive band (∼245 nm) and the backbone-sensitive band (∼200–210 nm), with DZN again producing larger decreases in ellipticity at lower doses, consistent with more extensive perturbation of backbone geometry and helical twist (Fig. 3c). Low-dose TEM further substantiated these findings where DZN achieved comparable fragmentation at 20-fold lower concentration (0.05 µM or higher) than DRN (1 µM) (Fig. 3d). The correlation between MD-derived overwinding/tilt/shift signatures and spectroscopic data supports a model in which DZN’s intercalation geometry imposes mechanical stress that destabilizes DNA duplex integrity at much lower ligand concentration than the standard DNA intercalators.

Moreover, natural polyphenolic intercalators such as berberine and ellipticine have shown anti-glioma potential, their development has been hampered by poor solubility, negligible BBB permeation, and systemic toxicity. DZN circumvents these issues with a glycosylated scaffold that enhances aqueous solubility and it’s isoflavone ring leads to moderate lipophilicity which helps DZN to cross BBB, which is an important criterion for any anti-glioma drug.

Quantum-chemical calculations clarify the chemical routes by which DZN promotes strand scission and oxidative DNA damage (Schemes 1 and 2). A canonical radical pathway (OSET to ^1^O_2_) was disfavored both kinetically and thermodynamically (∆*G*^‡^ = 29.3 kcal mol⁻¹; ∆*G*° ≈ 29.2 kcal mol⁻¹ at 37°C), in line with EPR-silent observations (Scheme 1 and Supplementary Fig. S2). Instead, ionic mechanisms proceeding via oxo-cation formation at the C4′ position were strongly supported in the presence of singlet oxygen and water. The ^1^O_2_-assisted path generated a thermodynamically stabilized spiro intermediate (A0; ∆_r_*G* = –16.8 kcal mol⁻¹; *K*_eq_ ≈ 10^11^) concomitantly releasing H_2_O_2_. Subsequent nucleophile-driven transformations (A0→A1) were exergonic (∆_r_*G* = –64.7 kcal mol⁻¹; *K*_eq_ ≈ 10^45^), consistent with deoxyribose fragmentation and nucleobase loss (A1RR) (Scheme 2). A complementary ^1^O_2_-initiated pathway at the guanine C8 yielded 8-OHdG and tautomerized 8-oxoG (B1→B1-OHST; ∆_r_*G* = –72.3 kcal mol^−1^; *K*_eq_ ≈ 10^50^), again producing H_2_O_2_ (Schemes 1 and 2). Together, the DFT results support a nonradical, ROS-coupled ionic mechanism, in which ^1^O_2_ initiates oxo-cation chemistry and H_2_O_2_ accumulation, thereby rationalizing both strand scission and oxidative base lesions in the DNA–DZN system.

These structural and chemical insights were mirrored in cellular and *in vivo* observations. In glioblastoma cell lines (LN18, LN229, and U87MG), DZN exhibited markedly lower IC_50_ values than TMZ while remaining non-toxic to normal glia up to 200 µM (Fig. 4a and Supplementary Fig. S3), indicating selective cytotoxicity. Treatment with DZN resulted in G1 cell cycle arrest, migration inhibition, and apoptosis (Fig. 4c–e) accompanied by activation of p-ATM, p-CHK2, and p-H2AX, together with accumulation of 8-OHdG (Fig. 5a–e and Supplementary Fig. S5), consistent with DSB, centered checkpoint activation and oxidative DNA damage. Nuclear leakage further suggested global chromatin and nuclear envelope destabilization (Fig. 5d). In a rat xenograft model, DZN at 5 mg·kg⁻¹ achieved significant tumor regression compared to TMZ (20 mg·kg⁻¹) without histopathological toxicity in liver, kidney, or brain tissues (Fig. 6b–h). Collectively, these findings support a dual-action mechanism in which DZN induces mechanical torsion within the DNA while simultaneously engaging ionic ROS chemistry that reinforces oxidative lesions, together overwhelming the glioma DNA damage response and enforcing apoptotic cell death.

The physicochemical attributes of DZN further enhance its therapeutic promise. The glycosylated scaffold improves aqueous solubility, while the isoflavone ring confers moderate lipophilicity sufficient for BBB penetration, thereby overcoming key limitations of other natural polyphenolic intercalators such as berberine or ellipticine. The distinct overwinding intercalation geometry defines a new structure–activity landscape, offering opportunities to modulate π–π stacking and torsional load without the cardiotoxic liabilities associated with anthracyclines. The strong activation of DNA damage signaling suggests potential synergy with DNA damage response inhibitors such as PARP or ATR antagonists, or with radiotherapy, where oxidative DNA lesions and DSB accumulation are therapeutically advantageous.

Despite these promising findings, certain limitations should be acknowledged. The molecular dynamics simulations were conducted on relatively short DNA duplexes using time-averaged parameters; extended simulations on longer, sequence-relevant genomic regions may reveal additional structural transitions pertinent to glioma biology. Furthermore, the proposed ionic and oxidative mechanisms were evaluated *in silico* under physiological conditions, and direct experimental quantification of intracellular singlet oxygen and hydrogen peroxide flux, along with comprehensive DNA lesion profiling, will be important for further validation. Collectively, our data strongly support oxidative DNA damage as the predominant driver of the observed DDR, rather than replication stress. However, as replication stress-specific pathways were not directly examined, a contributory role of replication-associated mechanisms cannot be fully excluded, representing a limitation of this study. Finally, although DZN demonstrates superior efficacy relative to TMZ in both *in vitro* and *in vivo* settings, its translational relevance warrants rigorous validation against clinically established anthracyclines and current glioma treatment regimens in orthotopic models. Notably, the present study is limited by the use of a subcutaneous C6 glioma model, which exhibits a relatively encapsulated growth phenotype and does not fully recapitulate the highly infiltrative nature of human glioblastoma.

In summary, DZN intercalates DNA through a “widening-plus-overwinding” geometry that differs fundamentally from classical anthracyclines, and engages a ROS-linked ionic mechanism leading to deoxyribose cleavage and oxidative base damage. These coupled structural and chemical perturbations trigger a robust DNA damage response, cell cycle arrest, and apoptosis in glioma cells while sparing normal glia over a wide concentration range. Our findings provide a mechanistic framework for structure-guided optimization of DZN analogues, where targeted modification of the aglycone and sugar moieties could refine π–π stacking geometry, groove anchoring, and aqueous compatibility, thereby enhancing DNA damage efficacy with minimal off-target effects.

## Supplementary Material

gkag397_Supplemental_File

## Data Availability

The data underlying this article will be shared on request to the corresponding author.
