# Supplementary material for: Coupling DNA intercalation with redox catalysis: selective killing mechanism of glioblastoma by daidzin
Source: Nucleic Acids Res. 2026 Apr 30;54(8):gkag397. doi: 10.1093/nar/gkag397 (PMC13129542; doi:10.1093/nar/gkag397)
Supplement: gkag397_Supplemental_File [file gkag397_supplemental_file.pdf]

Supplementary Information

Coupling DNA Intercalation with Redox Catalysis: Selective Killing mechanism of Glioblastoma by Daidzin

Shreya Banerjee [1], Sayan Paul [2†], Ranabir Majumder [1, 3†], Debolina Manna [1], Saugat Mondal [2], Dhruba Dhar [1], Souvik Karmakar[4], Jayasri das Sarma[4], Abhijit Das [5], N.D. Pradeep Singh[2], Soumen Das [1], Budhaditya Mukherjee [1], Anakuthil Anoop [6]*, Mahitosh Mandal [1]*

[1] School of Medical Science and Technology, Indian Institute of Technology, Kharagpur, 721302, India.

[2] Department of Chemistry, Indian Institute of Technology Kharagpur, Kharagpur 721302, India.

[3] Department of Physiological Sciences, Oklahoma State University, Stillwater, OK 74078, USA

[4] Department of Biological Science, Indian Institute of Science Education and Research, Kolkata, Mohanpur - 741 246, West Bengal, India

[5] Department of Bioscience and Biotechnology, Indian Institute of Technology, Kharagpur, 721302, India.

[6] School of Digital Sciences, Kerala University of Digital Science, Innovation, and Technology, Technopark Phase IV, Pallipuram, Thiruvananthapuram, Kerala 695317, India.

Corresponding Authors

***Mahitosh Mandal**

School of Medical Science and Technology, West Bengal, Indian Institute of Technology, Kharagpur, 721302, India

ORCID: 0000-0003-3861-3323

Email: mahitosh@smst.iitkgp.ac.in

***Anakuthil Anoop**

School of Digital Sciences, Kerala University of Digital Science, Innovation, and Technology, Thiruvananthapuram, Kerala, 695317, India.

ORCID: 0000-0002-8116-5506

Email: anoop.a@duk.ac.in

[†] Sayan Paul, Ranabir Majumder contributed equally to this work

DNA-Daunorubicin						
Step	Strand I			Strand II		
	P	O4'	C1'	P	O4'	C1'
CG/CG	6.46	3.81	3.13	10.61	8.37	7.95
GT/AC	9.73	6.32	6.05	6.85	3.98	3.87
TA/TA	8.47	7.04	6.04	8.99	6.81	6.20
AC/GT	9.16	6.04	5.47	11.06	8.08	7.48
CG/CG	7.23	5.11	5.11	9.22	7.34	6.74

DNA-Daidzin						
Step	Strand I			Strand II		
	P	O4'	C1'	P	O4'	C1'
CG/CG	9.60	6.75	6.40	6.53	3.52	3.16
GT/AC	10.52	7.45	6.70	8.07	6.93	6.00
TA/TA	8.97	6.03	5.29	10.03	7.49	6.59
AC/GT	9.24	6.09	5.41	10.94	7.97	7.36
CG/CG	9.10	6.59	6.13	7.82	5.43	4.94

Table S1: Radial displacement of P, O4′, and C1′ atoms in DNA upon ligand binding. The table summarizes the radial displacements P, O4′, and C1′ atoms in DNA when complexed with daunorubicin (DRN) and daidzin (DZN).

Base Pair Parameters	DNA					
	Buckle $(^0)$	Shear (Å)	Propeller $(^0)$	Opening $(^0)$	Stagger (Å)	Stretch (Å)
C1=G6	-3.65	1.512	-9.733	-1.237	0.029	-1.507
G2=C5	5.406	0.191	-6.607	0.428	0.125	-0.073
T3=A4	0.878	0.177	-6.192	-1.463	0.127	0.029
A4=T3	0.878	0.177	-6.192	-1.463	0.127	0.029
C5=G2	5.406	0.191	-6.607	0.428	0.125	-0.073
G6=C1	-3.65	1.512	-9.733	-1.237	0.029	-1.507

Base Pair Parameters	DNA-Daunorubicin					
	Buckle $(^0)$	Shear (Å)	Propeller $(^0)$	Opening $(^0)$	Stagger (Å)	Stretch (Å)
C1=G6	-24.625	-0.226	-4.239	-2.568	-0.43	-0.003
G2=C5	19.117	0.099	1.683	1.147	-0.181	0.01
T3=A4	2.558	0.229	-4.752	1.911	0.23	0.014
A4=T3	2.558	0.229	-4.752	1.911	0.23	0.014
C5=G2	19.117	0.099	1.683	1.147	-0.181	0.01
G6=C1	-24.625	-0.226	-4.239	-2.568	-0.43	-0.003

Base Pair Parameters	DNA-Daidzin					
	Buckle $(^0)$	Shear (Å)	Propeller $(^0)$	Opening $(^0)$	Stagger (Å)	Stretch (Å)
C1=G6	-31.756	6.75	20.838	-1.357	0.384	-2.07
G2=C5	15.611	0.28	-5.72	0.598	-0.079	-0.049
T3=A4	7.14	0.13	-3.781	-0.153	0.171	0.039
A4=T3	7.14	0.13	-3.781	-0.153	0.171	0.039
C5=G2	15.611	0.28	-5.72	0.598	-0.079	-0.049
G6=C1	-31.756	6.75	20.838	-1.357	0.384	-2.07

Table S2: Average intra–base-pair deformations induced by ligand binding. The table presents the average intra–base-pair parameters for ligand-bound DNA duplexes (DNA–DRN and DNA–DZN) relative to free DNA. Changes in buckle, propeller, opening, shear, stagger, and stretch are shown,

reflecting ligand-induced distortions in base-pair planarity, hydrogen-bond geometry, and in-/out-of-plane translational movements.

Base Step Parameters	DNA					
	Rise (Å)	Roll (^{0})	Shift (Å)	Slide (Å)	Tilt (^{0})	Twist (^{0})
C1=G6/G2=C5	3.671	8.523	0.191	-0.007	1.866	43.561
G2=C5/T3=A4	3.294	-1.192	0.171	-0.418	-0.505	32.723
T3=A4/A4=T3	3.191	1.775	-0.117	0.15	-0.324	36.115
A4=T3/C5=G2	3.294	-1.192	0.171	-0.418	-0.505	32.723
C5=G2/G6=C1	3.671	8.523	0.191	-0.007	1.866	43.561

Base Step Parameters	DNA-Daunorubicin					
	Rise (Å)	Roll (^{0})	Shift (Å)	Slide (Å)	Tilt (^{0})	Twist (^{0})
C1=G6/G2=C5	7.479	-1.092	-1.277	1.034	2.116	36.958
G2=C5/T3=A4	3.005	-1.367	1.02	-0.111	3.196	28.048
T3=A4/A4=T3	3.148	2.771	-0.026	0.046	0.04	43.188
A4=T3/C5=G2	3.005	-1.367	-0.026	-0.111	3.196	28.048
C5=G2/G6=C1	7.479	-1.092	-1.277	1.034	2.116	36.958

Base Step Parameters	DNA-Daidzin					
	Rise (Å)	Roll (^{0})	Shift (Å)	Slide (Å)	Tilt (^{0})	Twist (^{0})
C1=G6/G2=C5	7.537	0.034	0.238	0.471	-9.252	67.181
G2=C5/T3=A4	3.237	-0.839	0.476	-0.526	1.397	27.321
T3=A4/A4=T3	3.116	2.822	0.378	0.157	2.914	35.782
A4=T3/C5=G2	3.237	-0.839	0.476	-0.526	1.397	27.321
C5=G2/G6=C1	7.537	0.034	0.238	0.471	-9.252	67.181

Table S3: Average base-step parameters of ligand-bound DNA duplexes relative to free DNA. The table summarizes the average base-step parameters for DNA–DRN and DNA–DZN complexes compared with naked DNA. Variations in rise, roll, tilt, shift, slide, and twist reflect intercalation-induced widening, backbone-coupled bending, lateral translations, and alterations in helical winding associated with ligand binding.

Helical Parameters	DNA					
	Helical Rise (Å)	Helical Twist (^{o})	Inclination (^{o})	Tip (^{o})	X-displacement (Å)	Y-displacement (Å)
C1=G6/G2=C5	3.537	45.829	11.333	-2.524	-1.064	-0.031
G2=C5/T3=A4	3.225	33.492	-1.34	0.798	-0.678	-0.433
T3=A4/A4=T3	3.109	37.3	3.748	0.496	-0.222	0.156
A4=T3/C5=G2	3.225	33.492	-1.34	0.798	-0.678	-0.433
C5=G2/G6=C1	3.537	45.829	11.333	-2.524	-1.064	-0.031

Helical Parameters	DNA-Daunorubicin					
	Helical Rise (Å)	Helical Twist (^{o})	Inclination (^{o})	Tip (^{o})	X-displacement (Å)	Y-displacement (Å)
C1=G6/G2=C5	7.234	37.865	-1.559	-3.263	1.851	2.631
G2=C5/T3=A4	3.038	28.764	-2.648	-6.52	0.03	-1.377
T3=A4/A4=T3	3.093	43.954	3.954	-0.062	-0.259	0.038
A4=T3/C5=G2	3.038	28.764	-2.648	-6.52	0.03	-1.377
C5=G2/G6=C1	7.234	37.865	-1.559	-3.263	1.851	2.631

Helical Parameters	DNA-Daidzin					
	Helical Rise (Å)	Helical Twist (^{o})	Inclination (^{o})	Tip (^{o})	X-displacement (Å)	Y-displacement (Å)
C1=G6/G2=C5	7.106	73.628	1.197	5.725	0.219	-0.221
G2=C5/T3=A4	3.2	28.122	-1.603	-3.045	-0.933	-0.69
T3=A4/A4=T3	2.97	37.517	6.216	-4.428	-0.48	-0.154
A4=T3/C5=G2	3.2	28.122	-1.603	-3.045	-0.933	-0.69
C5=G2/G6=C1	7.106	73.628	1.197	5.725	0.219	-0.221

Table S4: Average helical descriptors of ligand-bound DNA duplexes relative to free DNA. The table presents the average helical parameters including H-rise, H-twist, inclination, tip, and X- and Y-displacements for DNA–DRN and DNA–DZN in comparison to naked DNA. These values capture ligand-induced global axis repositioning, variations in helical winding or unwinding, and translational offsets of the helical axis within the base-pair frame.

Computational Details:

Density functional theory (DFT) has been the method of choice for electronic structure calculations due to the ability to include (bulk) solvation effects easily [1,2] in reaction mechanisms. ORCA programme package (version 5.0.3) [3] is used in all our calculations. The primary geometric information is obtained from the last frame of the DNA-Daidzin complex from our 300 ns MD trajectory [4], where we have observed that Daidzin was intercalated between C1=G6/G2=C5 DNA bases and interacting with the Guanine base (G6) through a hydrogen bond (donor) in the DNA-ligand complex (**Fig. 1**). Geometry optimizations and frequency calculations are carried out in gas-phase using the generalized gradient approximation (GGA) with a Perdew-Burke-Ernzerhof exchange-correlation density functional (PBE) [5] along with Grimme's DFT-D4 dispersion correction [6] and a double-ζ valence basis with one set of polarization function (def2-SVP) [7,8] basis set. Density fitting with the Resolution of Identity (RI-J) method [9] is applied to speed up the calculation by using a suitable def2/J auxiliary basis set. In the main text, the basis set information for optimization and frequency calculation is denoted as BS1 and the method is denoted as PBE-D4/BS1. A denser grid setting 'defgrid3' that uses OptM3 with GC (2021) radial grid and Lebedev-590 angular grid with adaptive pruning and the Becke weight generation scheme with a basis function cutoff of $1.00e^{-11}$ are invoked in the optimization run. The lowest energy structure at the respective potential energy surface (PES) is verified by having only positive, real-frequency eigenvalues. It is important to note that the fully relaxed optimization of the adduct in the singlet potential energy surface (PES) is carried out considering that **DNA-DZN** is a tetra-anionic species. Therefore, the term used to denote the optimized structure of the **DNA-DZN** is given as ^{1}**[DNA-DZN]$^{4-}$**, where the prefix '1' suggests a singlet PES and the suffix stands for the tetra-anionic state of **DNA-DZN**.

Furthermore, the solvent-phase single-point calculation (SPE) is conducted using a larger triple-ζ def2-TZVP [7,8] basis set and TPSS meta-GGA functional [10,11] to improve the electronic energy. Additionally, Grimme's DFT-D4 dispersion correction [6] and auxiliary basis sets def2/J is used for this purpose on a denser grid setting 'defgrid3' that uses OptM3 with GC (2021) radial grid and Lebedev-590 angular grid with adaptive pruning. Density fitting with the Resolution of Identity (RI-J) method [9] is applied to speed up the calculation. To simulate the cell environment, we have considered the effect of water molecules on the final optimized structure (**DNA-DZN**). Hence, SMD [12] model is used to implicitly treat effects of solvent (water) during single poitnt energy calculation. The dielectric constant was set to 80.4 to

simulate the water solvent medium. The basis set information is termed BS2 and the method for SPE evaluation is denoted as SMD (water)-TPSS-D4/BS2 in the main text. The interaction energy between the DNA fragment and the intercalated Daidzin is calculated using the SPE of the ^{1}**[DNA-DZN]$^{4-}$**, the unrelaxed cutouts of the DNA fragment and Daidzin molecule: $\Delta \mathbf{E_{int}} = \mathbf{E_{DNA\text{-}DZN}} - (\mathbf{E_{DNA\text{-}frag}} + \mathbf{E_{DZN}})$. Here, **DZN** in E_{DZN} is a neutral species whereas the DNA-frag is tetra-anionic. Interaction energy calculations are performed in singlet PES.

Next, different routes to DNA-fragmentation are investigated using chemical intuition and previous literature reports [13–16], [17] .For this purpose, we have adopted the C-H activation followed by functional group modification of the DNA ribose sugar-phosphate back bone and Guanine base. The probable mechanistic pathway for the primary functional group modification (C-H to C-OH transformation) is shown in **Scheme 1**. Improved reaction Gibbs free activation energy values of the chemical intermediates ($\Delta_r G$) are estimated in 310K to account for the median basal body temperature of human. All calculations are performed assuming singlet potential energy surface (PES) due to the generation of singlet oxygen species in cancer cells [18]and the absence of EPR signal in ROS detection experiment (**Fig. S2**). Multiwfn [19] is used to obtain the Density of States plot. Structures and orbitals are generated using Jmol software [20]. The cartesian coordinates of the optimized structures are appended in **Table S10**.

In this work, we have considered two primary mechanisms that lead to the functional group modification (FGM) in DNA. In the radical mechanism, a total of two Outer Single Electron Transfer (OSET) reactions were studied: (i) the reaction between the ground state of the adduct ^{1}**[DNA-DZN]$^{4-}$** and ^{1}**H_2O_2**; (ii) the reaction between the ground state of the adduct ^{1}**[DNA-DZN]$^{4-}$**and triplet oxygen ^{3}**O_2**.

The two reactions are given as follows –

^{1}**[DNA-DZN]$^{4-}$ + $^{1}H_2O_2 \rightarrow$ ^{2}[DNA-DZN]$^{3-}$ + $^{2}H_2O_2^{-\bullet}$** (r.a) (λ^{a})

^{1}**[DNA-DZN]$^{4-}$ + $^{1}O_2 \rightarrow$ ^{2}[DNA-DZN]$^{3-}$ + $^{2}O_2^{-\bullet}$** (r.b) (λ^{b})

To calculate, the thermodynamic barriers involved, we have broken down each of these two reactions into two half-reactions and determined the OSET barriers using **eq. 1**. For example, the two OSET reactions originating from **r.a** are as follows –

^{1}**[DNA-DZN]$^{4-}$ + ^{2}[DNA-DZN]$^{3-}$ $\rightarrow$ ^{2}[DNA-DZN]$^{3-}$ + ^{1}[DNA-DZN]$^{4-}$** (r.a.1) (λ_{11}^{a})

$^{1}H_2O_2 + ^{2}H_2O_2^{-\bullet} \rightarrow ^{2}H_2O_2^{-\bullet} + ^{1}H_2O_2$ (r.a.2) (λ_{22}^{a})

The $^{2}H_2O_2^{-\bullet}$ can dissociate in the following stepwise way under an OSET-SW mechanism –

$^{2}H_2O_2^{-} \rightarrow ^{1}OH^{-} + ^{2}OH^{\bullet}$

The same principle is applied to **r.b** for evaluating the OSET reaction barrier with triplet oxygen.

^{1}**[DNA-DZN]**$^{4-}$ + ^{2}**[DNA-DZN]**$^{3-}$ → ^{2}**[DNA-DZN]**$^{3-}$ + ^{1}**[DNA-DZN]**$^{4-}$ (r.b.1) (λ_{11}^{b})

$^{1}O_2 + ^{2}O_2^{-\bullet} \rightarrow ^{2}O_2^{-\bullet} + ^{1}O_2$ (r.b.2) (λ_{22}^{b})

λ^{a} and λ^{b} are the reorganization energies for reaction **r.a** and **r.b**, respectively. In DFT calculations, individual reorganization energies (λ_{11}^{a} and λ_{22}^{a}) are calculated by subtracting the structurally optimized energy of the reactant (product) from unrelaxed energy of itself at the relaxed coordinate of the corresponding product (reactant). In **r.a.1** and **r.b.1**, the unrelaxed energy of the product ^{2}**[DNA-DZN]**$^{3-}$ is the energy on the potential surface of ^{2}**[DNA-DZN]**$^{3-}$ calculated by using the optimized geometry of ^{1}**[DNA-DZN]**$^{4-}$. Similarly, the unrelaxed energy of the product $^{2}H_2O_2^{-\bullet}$ in **r.a.2** is the energy on the potential surface of $^{2}H_2O_2^{-}$ calculated using the optimized geometry of $^{1}H_2O_2$. In **r.b.2**, the unrelaxed energy of the product $^{2}O_2^{-}$ is the energy on the potential surface of $^{2}O_2^{-\bullet}$ calculated by using the optimized geometry of $^{1}O_2$. To account for the effects of solvent on the reorganization energy, non-equilibrium parts of the implicit solvent (the slow/inertial charges) obtained from the optimized energy calculations were used in the corresponding unrelaxed energy calculations followed by an external iteration procedure. We have followed the same principle to obtain $\lambda_{11}^{a}, \lambda_{22}^{a}, \lambda_{11}^{b}$, and λ_{22}^{b}.

At this point, we can apply the general principle of self-exchange reactions of two simultaneous OSET reactions (**r.a.1** and **r.a.2**) to understand the complete OSET mechanism. Marcus's theory is used to calculate the OSET reaction activation energy barriers [21, 22]. The Gibbs free energy barrier for the OSET reaction was calculated using Marcus's theory as formulated in the following equation: [23–25]

$$\Delta G_{OSET}^{\ddagger} = \frac{\lambda}{4}\left(1 + \frac{\Delta G_{OSET}^{o}}{\lambda}\right)^{2} \qquad \text{(eq. 1)}$$

where ΔG_{OSET}^{o} is the Gibbs free energy of the OSET reaction, λ is the total reorganization energy. The reorganization energy (λ) is approximately determined by averaging the self-

exchange reorganization energy of individual reactants in the OSET reactions [26] as given below.

The total reorganization energy (λ^a or λ^b) is approximated as follows:

$$\lambda^a = \frac{\lambda_{11}^a + \lambda_{22}^a}{2} \qquad \text{(eq. 2)}$$

The above calculations yield two OSET barriers corresponding to **r.a** and **r.b** reactions.

Rx	Optimized species	**^{1}[DNA-DZN]$^{4-}$**	**^{2}[DNA-DZN]$^{3-}$**	**$^1O_2$**	**$^2O_2^{-\bullet}$**
**r.a**	E_{elec} (Eh)	-8780.284359	-8780.099440	-150.4132286	-150.547672
	G (Eh)	-8778.657586	-8778.474794	-150.4295383	-150.565740
	Unrelaxed species	**^{1}[DNA-DZN]$^{4-}$ /^{2}[DNA-DZN]$^{3-}$**	**^{2}[DNA-DZN]$^{3-}$ /^{1}[DNA-DZN]$^{4-}$**	**$^1O_2$/ $^2O_2^{-\bullet}$**	**$^2O_2^{-\bullet}$/ $^1O_2$**
	E_{elec}^U (Eh)	-8780.283313	-8780.092985	-150.3382886	-150.5268588
**r.b**	Optimized species	**^{1}[DNA-DZN]$^{4-}$**	**^{2}[DNA-DZN]$^{3-}$**	**$^1H_2O_2$**	**$^2H_2O_2^{-\bullet}$**
	E_{elec} (Eh)	-8780.284359	-8780.099440	-151.6465551	-151.7537938
	G (Eh)	-8778.657586	-8778.474794	-151.6440965	-151.7729322
	Unrelaxed species	**^{1}[DNA-DZN]$^{4-}$ /^{2}[DNA-DZN]$^{3-}$**	**^{2}[DNA-DZN]$^{3-}$ /^{1}[DNA-DZN]$^{4-}$**	**$^1H_2O_2$/$^2H_2O_2^{-\bullet}$**	**$^2H_2O_2^{-\bullet}$/$^1H_2O_2$**
	E_{elec}^U (Eh)	-8780.283313	-8780.092985	-151.5210934	-151.658552

Table S5. Calculation of the OSET barriers with reactants **$^1O_2$** and **$^1H_2O_2$** where **^{1}[DNA-DZN]$^{4-}$** is the common reactant. [a]Electronic energies (E_{elec}) and Gibbs free energies (G) of the structurally optimized species, and their corresponding unrelaxed electronic energies (E_{elec}^U) of the OSET reactions (**r.a** and **r.b**) mentioned below (in Hartree, 310 K). Note that X/Y denotes the geometry of the species X taken from the optimized geometry of the species Y.

[a]Unless otherwise stated, all the calculations performed at the SMD (water)-TPSS-D4/BS2//PBE-D4/BS1 level of theory. Closed-shell and open-shell wavefunctions are used wherever necessary. Calculation of G includes a correction for change of state from 1atm to 1M.

Reaction	$\Delta G_{OSET}^{\ddagger}$	λ	ΔG_{OSET}^{o}
**r.a**	29.3	32.4	29.2
**r.b**	38.8	71.6	33.9

Table S6. Reaction barriers $\Delta G_{OSET}^{\ddagger}$ of the two OSET reactions calculated using Eq. 1. λ for both OSET reactions (λ^a and λ^b) are calculated using Eq. 2. ΔG_{OSET}^{o} stands for the Gibbs free

energy of the OSET reactions. [a]Values of these energy parameters are given in kcal mol^{-1} unit at 310 K.

[a]Unless otherwise stated, all the calculations performed at the SMD (water)-TPSS-D4/BS2//PBE-D4/BS1 level of theory. Closed-shell and open-shell wavefunctions are used wherever necessary. Calculation of G includes a correction for change of state from 1atm to 1M.

Species	E_{elec} (Eh) (SMD-TPSS-D4/BS2)	G_{corr} (Eh, 310 K) (PBE-D4/BS1)	G (Eh, 310 K)
R0 (^{1}[DNA-DZN]$^{4-}$)	-8780.284359	1.62677277	-8778.657586
Unrelaxed DNA-frag	-7290.022770	-	-
Unrelaxed DZN	-1490.168335	-	-
^{2}[DNA-DZN]$^{3-}$	-8780.099440	1.62464673	-8778.474794
P2	-8780.185697	1.62682302	-8778.558874
R0-H_2O	-8856.767224	1.65274325	-8855.114481
R0-O_2	-8930.645385	1.63001699	-8929.015368
A0	-8779.510397	1.61958566	-8777.890812
H_2O_2 ($^1H_2O_2$)	-151.6465551	0.00245863	-151.6440965
H_2O	-76.47704416	0.00215199	-76.47489217
$^2H_2O_2^{-\bullet}$	-151.7822482	-0.00587759	-151.7881257
OH^-($^1OH^-$)	-75.97264029	-0.00950770	-75.98214799
$^2OH^\bullet$	-75.78115356	-0.00963070	-75.79078426
$^1O_2$	-150.4132286	-0.01723773	-150.4304664
$^2O_2^{-\bullet}$	-150.5476720	-0.01806813	-150.5657402
A1	-8855.560826	1.63086194	-8853.929964
A2	-8779.453143	1.61959660	-8777.833547
B1	-8779.500428	1.61867364	-8777.881754
A1R	-8855.543879	1.62775157	-8853.916128
A1RR	-8855.559237	1.62235978	-8853.936877
A2-OHS	-8855.489140	1.63192719	-8853.857213
B1-OHS	-8855.564371	1.62677956	-8853.937592
B1-OHST	-8855.570380	1.62842849	-8853.941952

Table S7. Energy components (Hartree, 310 K), used to calculate the Gibbs free energies of all DFT-optimized species.

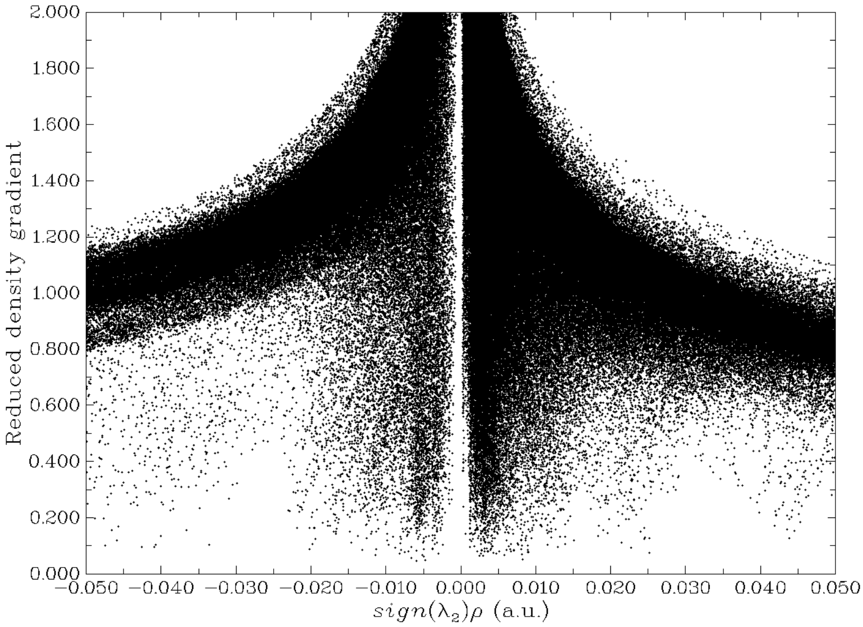

Figure S1. Graphical description of the Non-covalent Interaction (NCI) of DNA-DZN adduct calculated at SMD (water)-TPSS-D4/BS2//PBE-D4/BS1 level of theory. Sign(λ_2)ρ is shown on the X-axis, and the Reduced Density Gradient is plotted along the Y-axis

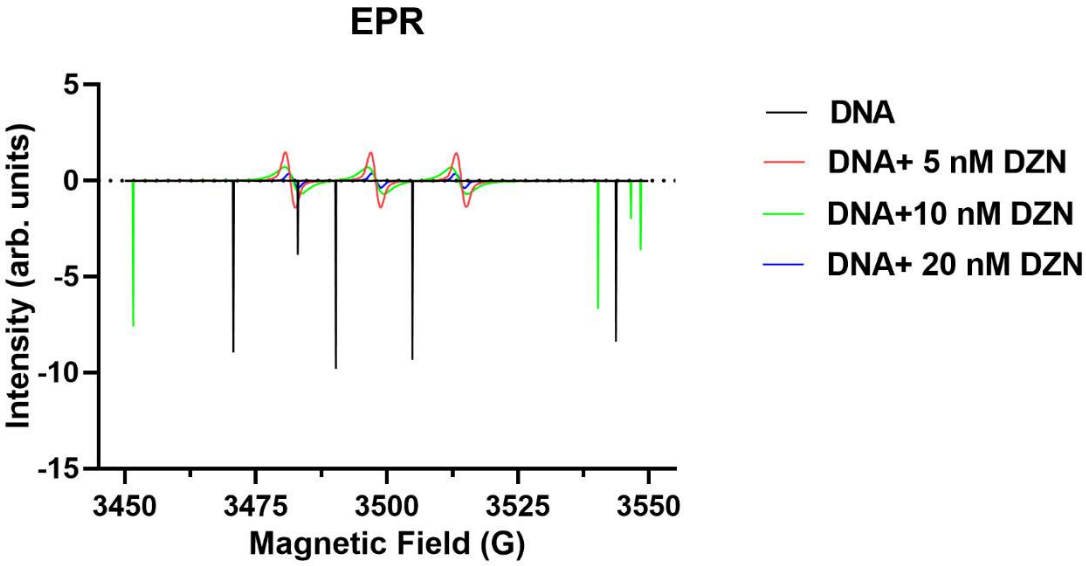

Figure S2: EPR spectra of DNA and DNA–DZN complexes showing absence of paramagnetic species. Electron paramagnetic resonance (EPR) spectra recorded for free DNA and DNA incubated with increasing concentrations of DZN (5, 10, and 20 nM) at room temperature.

EPR spectroscopy was performed to investigate the presence of paramagnetic intermediates generated upon ligand binding to DNA. Measurements were carried out using an X-band EPR

spectrometer at room temperature within a magnetic field range of 3450–3550 G. Samples of free DNA and DNA incubated with increasing concentrations of DZN (5, 10, and 20 nM) were analysed under identical conditions.

As shown in the EPR spectra, no characteristic signal corresponding to radical or oxygen-centered species was detected in any of the samples, indicating the absence of detectable paramagnetic intermediates. Complementary DFT calculations support this observation, revealing that the most favorable reaction pathway involves a singlet oxygen ($1O_2$)–assisted mechanism leading to the formation and release of hydrogen peroxide (H_2O_2). Since both $1O_2$ and H_2O_2 are diamagnetic species, no EPR signal is expected [27], consistent with the experimental findings.

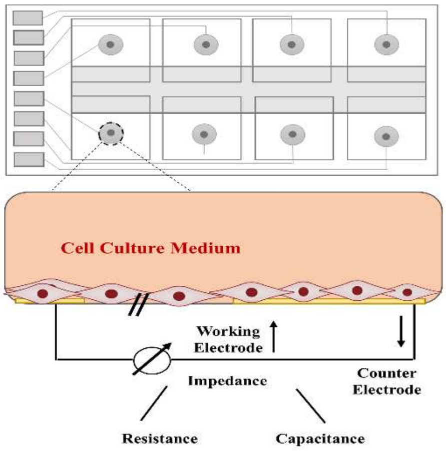

Cell	R_{med} (Ohm)	R_{cyto} (Ohm)	C_{mem} (nF)	n	Q
Ln18	1E06	1.7E05	6.79	0.15	8.5E-05
SVGP12	1.5E05	36955	1.4	0.77	1.42E-08
Ln229	1E06	1.3E05	6.84	0.16	7.8E-05

Table S8. Impedance characterization of glioma (LN18, LN229) and normal glial (SVGP12) cell lines. Electrical impedance spectroscopy (EIS) was performed to evaluate cell-specific membrane and cytoplasmic electrical properties. The fitted equivalent circuit parameters—membrane resistance (R_{me}d), cytoplasmic resistance (Rcyto), membrane capacitance (Cmem), constant phase element exponent (n), and pseudo-capacitance (Q)—were extracted from impedance spectra. SVGP12 normal glial cells exhibited significantly higher overall impedance than LN18 and LN229, consistent with greater membrane integrity and lower passive permeability.

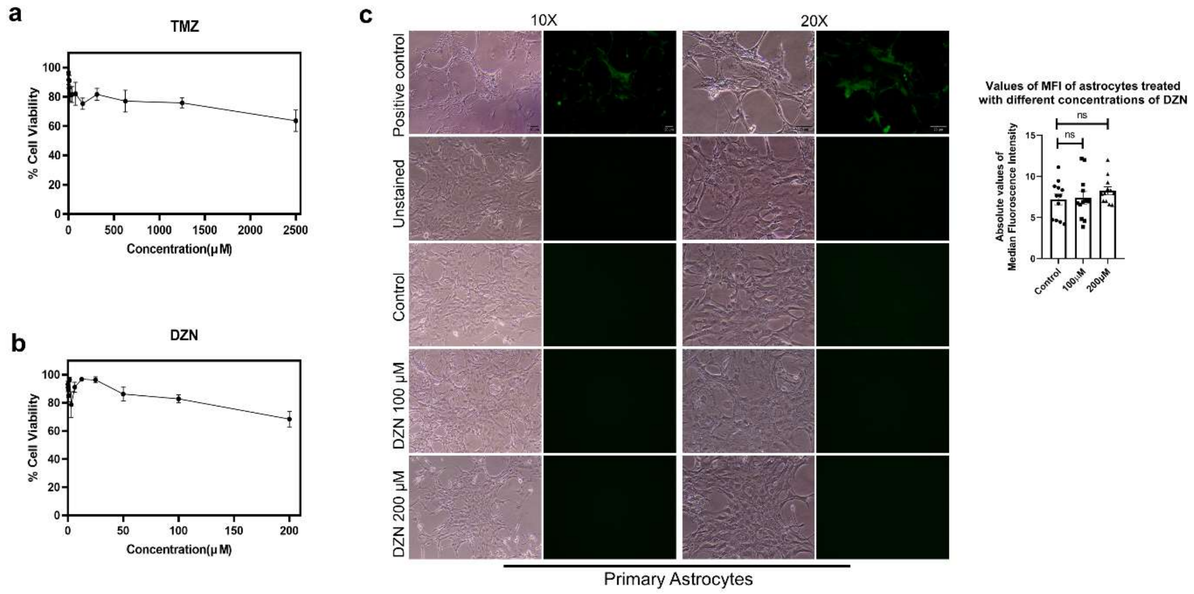

Figure S3: Evaluation of cytotoxicity and oxidative stress induction by DZN in primary astrocytes. (a–b) Primary astrocytes were treated with increasing concentrations of DZN (a) and Temozolomide (TMZ) (b) for 48 h, and cell viability was assessed. DZN exhibited minimal cytotoxicity even at higher concentrations, indicating limited toxicity toward non-cancerous astrocytes. Data are presented as mean ± SEM of three independent replicates. (c) Intracellular ROS levels were measured using a DCFDA assay following treatment with DZN (100 and 200 μM). Cells were incubated with 10 μM DCFDA for 30–40 min post-treatment. tert-Butyl hydroperoxide (TBHP) was used as a positive control to validate assay performance. No significant ROS generation was observed in DZN-treated astrocytes compared to control conditions. Data are presented as mean ± SEM of three independent replicates.

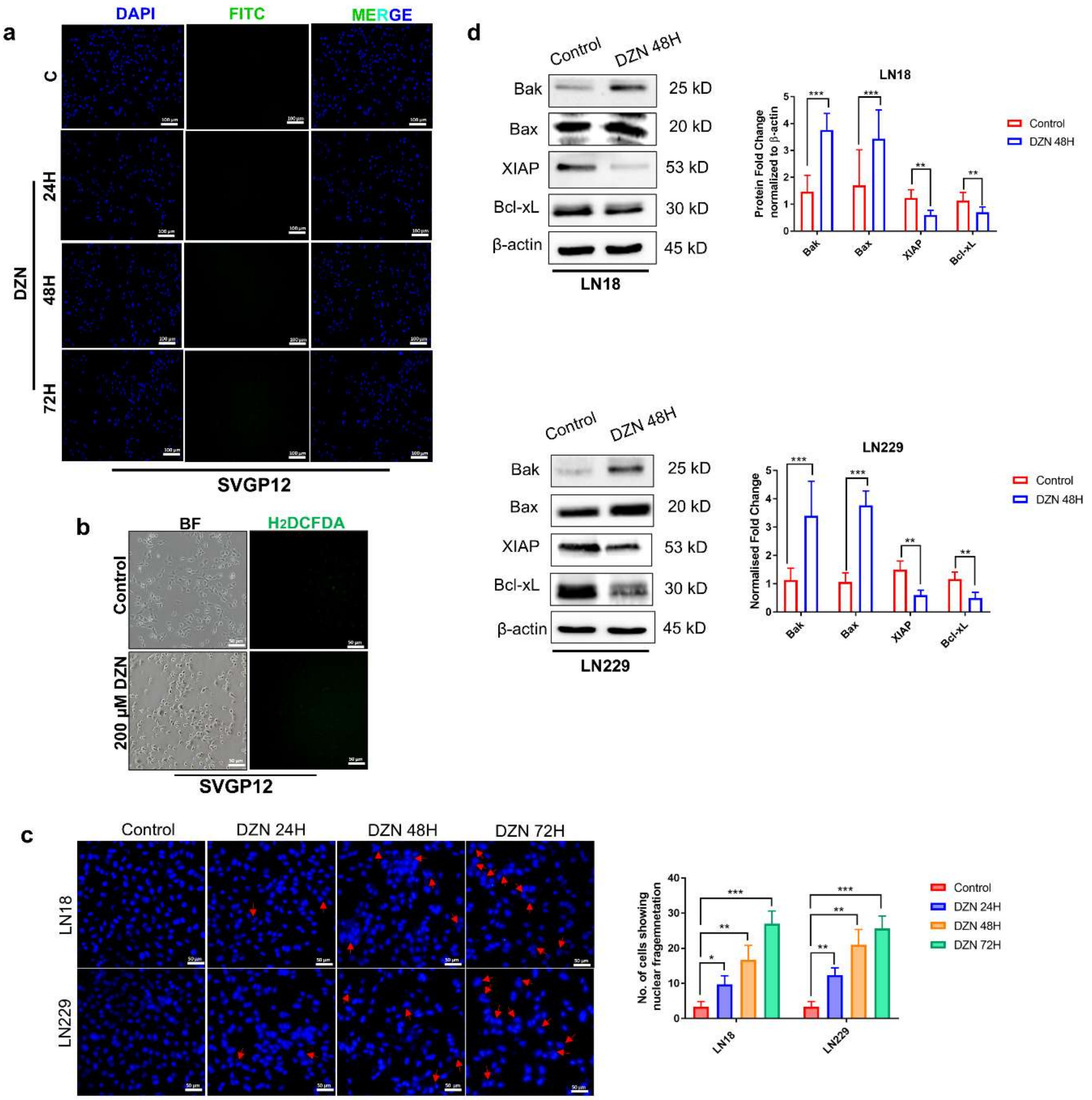

Figure S4: (a) TUNEL assay of SVGP12 cells after 24, 48, and 72 h of DZN exposure shows no evidence of DNA fragmentation. (b) SVGP12 cells were treated with DZN (200 µM) for 48 h. Intracellular ROS levels were assessed using DCFDA staining. No significant increase in fluorescence was observed compared to control. (c) Time dependent Hoechst staining of DZN-treated LN18 and LN229 cells reveals nuclear condensation and fragmentation, corroborating IFC results. (d) Western blots indicate increased expression of the pro-apoptotic marker Bak, Bax and decreased expression of the anti-apoptotic marker Bcl-xL, XIAP in LN18 and LN229 cells, confirming DZN-induced genomic instability and apoptosis in glioma.

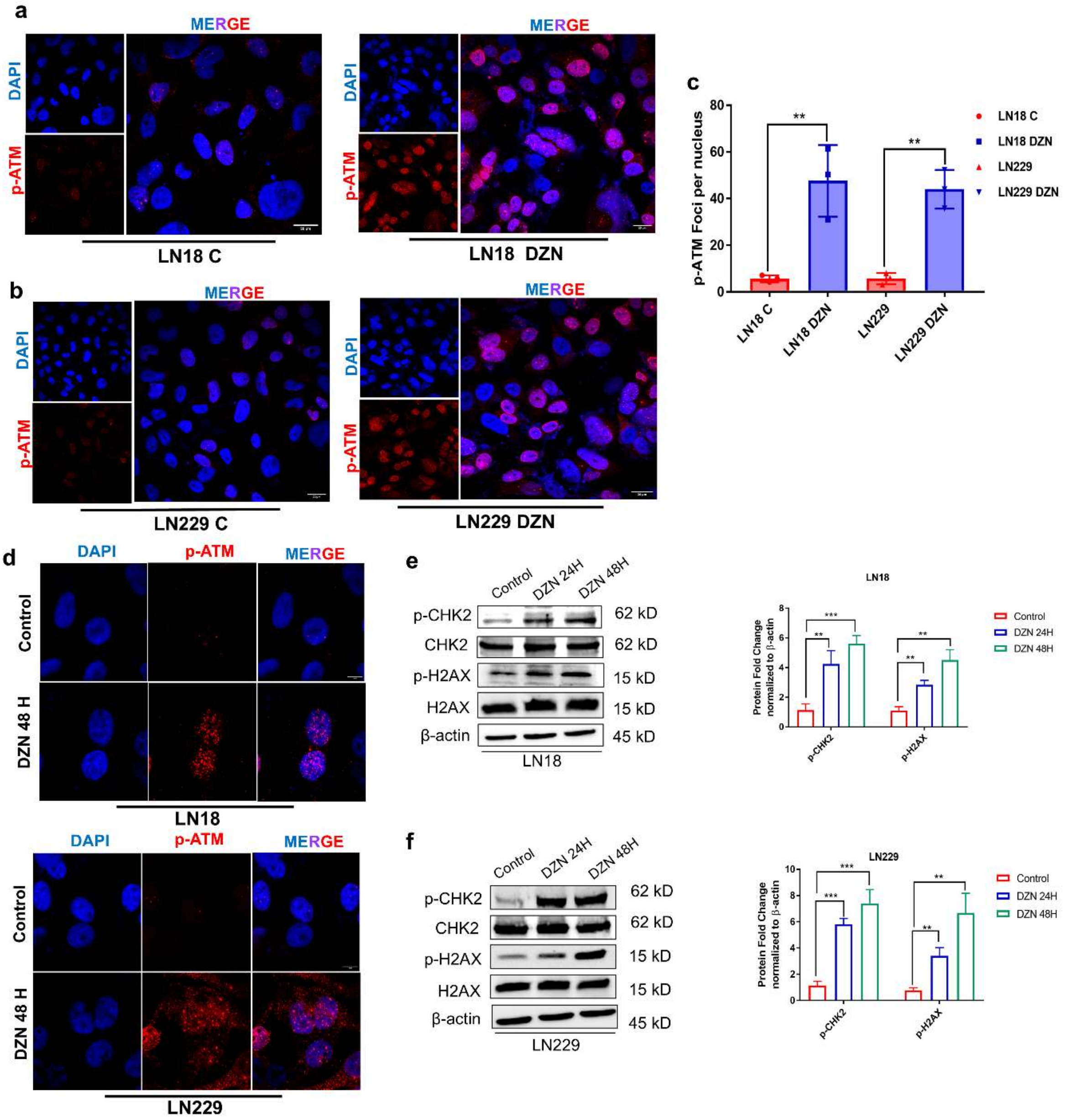

Figure S5: DZN-induced DNA damage in LN18 and LN229 cell lines. (a, b) Immunofluorescence cytometry (IFC) images showing increased formation of phosphorylated ataxia-telangiectasia mutated (p-ATM) foci in LN18 and LN229 cells, respectively, following DZN treatment. (c) Quantification of p-ATM foci in LN18 control, LN18 + DZN, LN229 control, and LN229 + DZN groups, with statistical significance indicated as *P < 0.05, **P < 0.01, ***P < 0.001, ****P < 0.0001. (d) Representative IFC images of selected cells displaying p-ATM foci at 60× magnification. (e, f) Western blot analysis and corresponding densitometric quantification of p-CHK2, total CHK2, p-H2AX, and total H2AX in (e) LN18 and (f) LN229 cells. Statistical significance is denoted as *P < 0.05, **P < 0.01, ***P < 0.001, ****P < 0.0001.

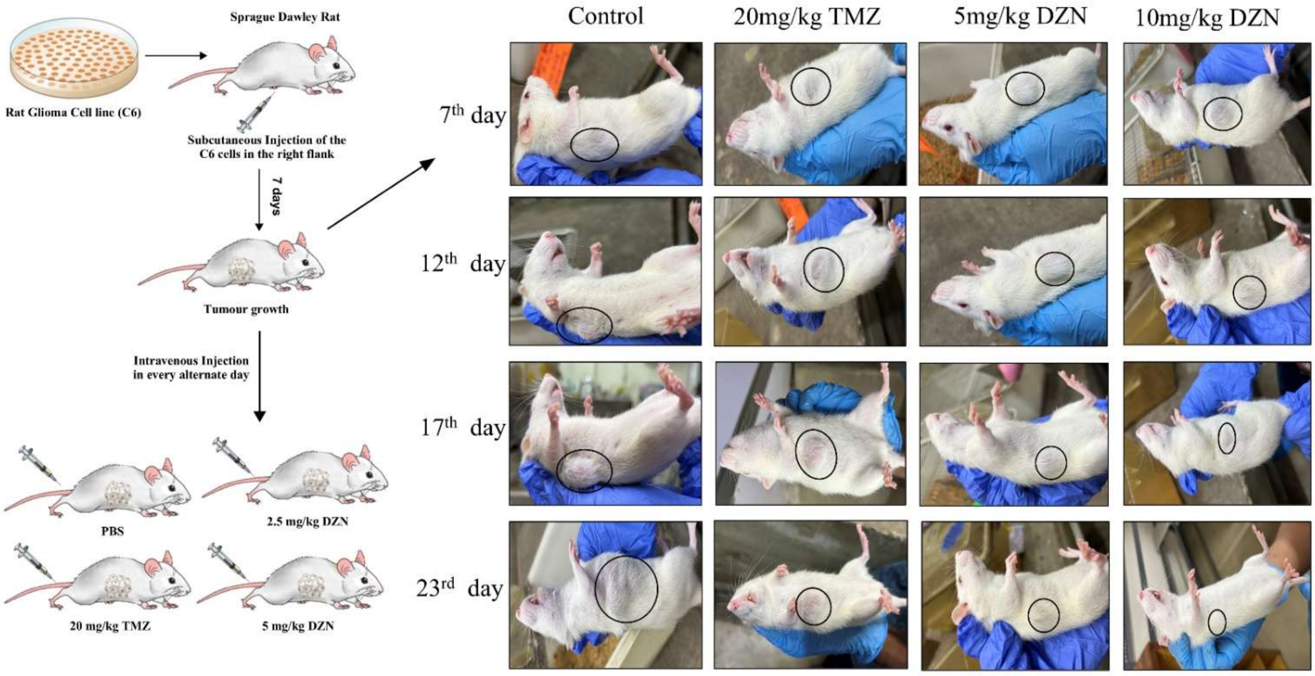

Figure S6: In vivo evaluation of DZN efficacy in a C6 glioma xenograft model. Representative images of Sprague–Dawley rats bearing subcutaneous C6 glioma tumors. Animals were randomized into four groups: control, 20 mg/kg temozolomide (TMZ), 5 mg/kg DZN, and 10 mg/kg DZN. Treatments commenced on day 7 and were administered on alternate days. Tumor volume was monitored on days 7, 11, 15, 19, and 23, followed by final tumor excision and weight measurement on day 23. DZN treatment resulted in a marked reduction in tumor volume and weight compared with the control, with the 5 mg/kg DZN group exhibiting antitumor efficacy comparable to 20 mg/kg TMZ.

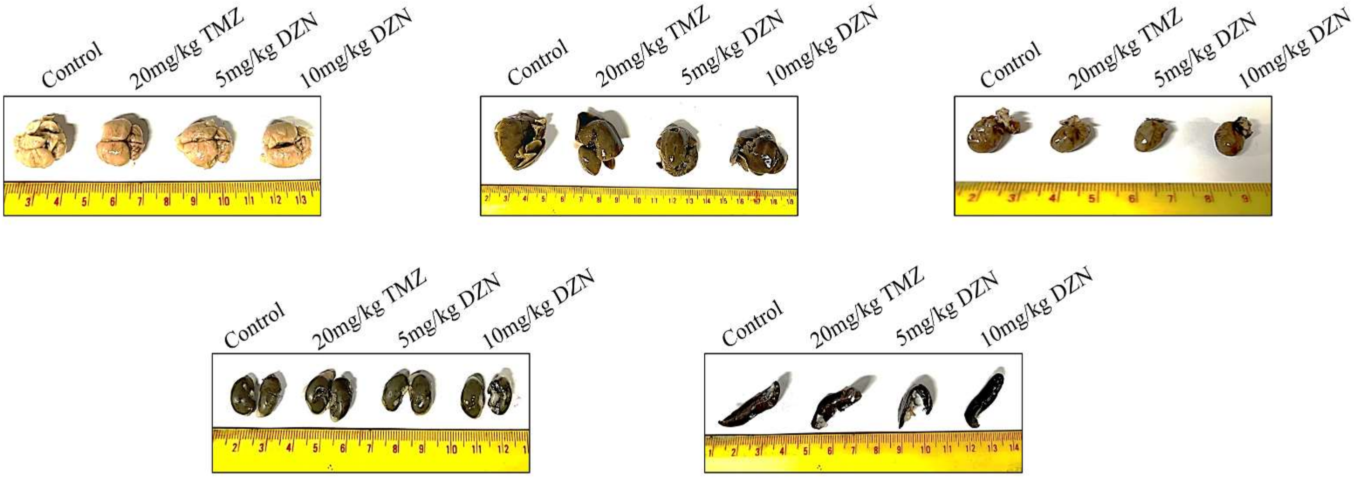

Figure S7: Gross examination of major organs following DZN and TMZ treatment. Representative images of heart, spleen, kidney, brain, and liver excised from all experimental groups on day 23. No overt morphological differences or signs of toxicity were observed in DZN- or TMZ-treated animals compared with tumor-bearing controls.

	Control	20mg/kg TMZ	5 mg/kg DZN	10mg/kg DZN
Haemoglobin (g/dl)	13.1	11.8	10.2	12.6
Total WBC Count (thou/mm3)	6.2	5.2	3.8	6.1
RBC (milli/mm3)	4.8	4.6	4.2	4.5
PCV/HCT (%)	40.2	35.8	37	39.2
MCV (fL)	69.1	56.4	66	68.2
MCH (pg)	23.6	24.5	22.4	21.9
MCHC (g/dl)	32.6	31.6	32.6	31.3
Platelet (thou/mm3)	215	181	195	252
Neutrophil (%)	33	36	39	29
Lymphocyte (%)	65	58	60	62
Eiosinophil (%)	1	3	1	3
Monocyte (%)	1	3	0	5
Besophil (%)	0	0	0	1
Bilirubin-Total (mg/dl)	0.78	0.58	0.72	0.78
Bilirubin-Direct (mg/dl)	0.39	0.41	0.4	0.52
Bilirubin-Indirect (mg/dl)	0.39	0.17	0.32	0.26
SGOT (U/L)	30.8	33.2	31.5	20.2
SGPT (U/L)	21.6	29.4	35.2	18.6
Total Protein (g/dl)	5.8	5.8	5.8	6.2
Albumin (g/dl)	3.1	3.2	4.2	4.3
Urea (mg/dl)	18.2	26	23	19.5
Creatinine (mg/dl)	0.58	0.66	0.6	0.64
Uric Acid (mg/dl)	3.8	4.9	5.4	5.1
Sodium (mEq/L)	126	128	126	129
Potassium (mEq/L)	3.4	3.9	4.1	3.2
Chloride (mEq/L)	101.6	101.8	97.2	96.8
Calcium (mg/dl)	8.8	9.2	9.5	9.2

Table S9: Complete blood profile of animals across experimental groups. Haematological parameters were measured in animals from four groups to assess systemic effects and potential treatment-related toxicity

Table S10. Cartesian coordinates of the DFT optimized structures.

R0

O	20.93323	27.49309	17.45590
C	19.74242	27.95763	16.85476
C	19.39047	27.23022	15.56402
O	20.45766	27.35209	14.60988
C	20.27883	26.29917	13.67475
N	21.51931	26.10970	12.92844
C	22.68891	25.98602	13.61220
C	23.86525	25.68449	12.97421
C	23.76958	25.47738	11.54769
N	24.87348	25.09590	10.86371
N	22.63895	25.65203	10.85970
C	21.46868	25.98107	11.48530
O	20.39466	26.15594	10.90645
C	19.06803	25.71000	15.68921
C	19.85396	25.08650	14.52261
O	17.67814	25.46105	15.53060
P	16.71785	25.30691	16.91396
O	15.33411	24.96062	16.42524
O	17.07257	26.39857	17.91702
O	17.45585	23.94139	17.60146
C	17.34457	22.66490	17.02258
C	17.80906	21.62490	18.04392
O	19.13284	21.95142	18.52121
C	19.05232	22.55788	19.82209
N	19.80844	23.79441	19.86477
C	19.42330	25.10038	19.58138
N	20.44377	25.94129	19.63294
C	21.55029	25.16781	19.94091
C	22.94378	25.50123	20.02766
O	23.45556	26.63764	19.98102
N	23.74297	24.34719	20.13821
C	23.27632	23.05144	20.20293
N	24.20162	22.06256	20.21756
N	21.97225	22.74474	20.23597
C	21.18120	23.81865	20.05940
C	16.97224	21.54394	19.33084
C	17.56473	22.67480	20.17165
O	17.27308	20.28668	19.95950
P	16.11751	19.43449	20.80678
O	16.84640	18.10230	21.12970
O	14.77985	19.48912	20.12612
O	16.00171	20.32384	22.22748
C	16.76495	20.01673	23.37748
C	18.25819	20.24809	23.26247
O	18.58811	21.63167	23.09524

C	19.98621	21.66873	23.31671	N	24.92552	24.89972	23.38133
N	20.42557	23.06272	23.35384	C	25.71502	23.81255	23.52551
C	19.55820	24.11696	23.16770	N	27.05071	23.77517	23.55854
C	19.98154	25.41499	23.07381	C	27.58081	25.00133	23.39100
C	19.06984	26.57584	22.81929	C	31.70825	24.81881	22.06451
C	21.41535	25.67793	23.18163	C	30.67034	23.77156	22.45575
O	21.90124	26.82464	23.14545	O	32.78283	24.37889	21.27386
N	22.22475	24.55952	23.34160	P	33.27528	25.41046	20.00471
C	21.81980	23.24751	23.41969	O	34.75113	25.17989	19.81621
O	22.59040	22.29580	23.55428	O	32.61658	26.77612	20.23452
C	19.06844	19.76969	24.50265	O	32.39051	24.71299	18.75506
C	20.19538	20.84102	24.59334	C	32.71417	23.35371	18.48493
O	19.49289	18.43313	24.41602	C	31.55915	22.64262	17.79474
O	32.03364	27.65275	22.64627	O	30.40139	22.66710	18.64564
C	32.77808	26.73357	23.39877	C	29.29875	23.26504	17.97164
C	32.19984	25.31255	23.43178	N	28.56501	24.15413	18.88248
O	31.05876	25.20310	24.30776	C	29.14928	25.32308	19.29450
C	30.03573	24.44635	23.68114	C	28.41096	26.31638	19.87627
N	28.91646	25.35113	23.37114	C	26.98843	26.10250	19.95606
C	28.96056	26.72349	23.17110	N	26.14623	27.10673	20.23381
N	27.75881	27.27529	23.07022	N	26.46826	24.87924	19.73912
C	26.88791	26.21473	23.20978	C	27.22370	23.85175	19.25725
C	25.46965	26.13560	23.22708	O	26.78227	22.69747	19.11630
N	24.67016	27.21175	23.12168	C	31.13359	23.20549	16.42021

C	29.88378	24.01384	16.76841	O	22.00661	18.97328	19.58715
O	30.85800	22.09405	15.55836	O	22.17862	19.28196	17.34824
P	30.72390	22.37361	13.91326	O	18.35043	17.05420	19.14153
O	30.51887	21.02695	13.24866	O	19.36618	17.67049	21.81490
O	31.75475	23.40839	13.46711	O	19.43027	18.37590	17.03963
O	29.25783	23.23464	13.87389	O	23.08708	17.60060	21.64099
C	28.05442	22.55965	13.56872	O	22.31329	24.05470	17.00550
C	27.61317	22.91132	12.15656	O	26.31363	23.99694	16.09926
O	27.31750	24.33192	12.03394	O	26.90818	30.39958	16.61244
C	28.44953	25.04337	11.52142	C	19.36450	18.01251	19.40307
N	28.96304	26.00645	12.48657	C	20.19139	17.74076	20.65818
C	30.18060	26.07079	13.17842	C	20.24904	18.11233	18.16166
N	30.28361	27.15176	13.92564	C	21.21987	18.88756	20.78410
C	29.09068	27.82396	13.75232	C	21.25110	19.23965	18.39890
C	28.57684	29.01194	14.36502	C	22.23147	18.69820	21.91223
O	29.09389	29.77695	15.20549	C	22.83391	20.46938	17.12355
N	27.24458	29.26536	13.93424	C	22.18654	21.70898	17.15603
C	26.45970	28.42195	13.17836	C	24.21681	20.38085	16.82533
N	25.12389	28.74128	13.07840	C	22.96234	22.85482	16.92971
N	26.94973	27.36252	12.55722	C	24.34249	22.80682	16.65378
C	28.24103	27.11444	12.87488	C	24.95564	21.53454	16.59229
C	28.65040	22.67301	11.04373	C	25.12578	24.04325	16.46716
C	29.46055	23.98477	11.04664	C	24.38146	25.29402	16.74637
O	28.01203	22.43105	9.78431	C	23.02399	25.20208	16.95872

C	25.03658	26.62384	16.75981
C	26.44648	26.73980	16.81305
C	24.28507	27.82805	16.76510
C	27.08314	27.98308	16.84274
C	24.92029	29.07594	16.75065
C	26.32941	29.16973	16.75975
H	20.71523	27.09295	18.35316
H	18.85734	27.82039	17.51680
H	19.84450	29.04599	16.62038
H	18.46084	27.69123	15.14694
H	19.51334	26.55075	12.90799
H	22.62580	26.15882	14.69586
H	24.80725	25.55906	13.52502
H	24.75366	24.95146	9.86258
H	25.77139	24.90077	11.34009
H	19.43162	25.31194	16.66019
H	20.75220	24.55412	14.89198
H	19.22088	24.37351	13.96275
H	17.99121	22.58138	16.11569
H	16.29555	22.45641	16.70501
H	17.87397	20.62550	17.56313
H	19.57491	21.87753	20.52563
H	18.40594	25.38769	19.26422
H	25.16952	22.26002	19.88136
H	23.81926	21.12818	20.06201
H	15.88066	21.62505	19.15003
H	17.38038	22.51220	21.24736
H	17.15364	23.64140	19.83242
H	16.64368	18.94695	23.66698
H	16.36570	20.65007	24.20182
H	18.64325	19.68547	22.38635
H	20.53196	21.19188	22.46787
H	18.50250	23.82828	23.07998
H	18.01175	26.25528	22.75703
H	19.34520	27.06141	21.85998
H	19.17312	27.34709	23.61180
H	18.42662	19.85210	25.41259
H	20.06363	21.49946	25.47567
H	21.19910	20.38517	24.63835
H	19.54078	18.16013	23.45705
H	32.22999	27.42098	21.67354
H	33.82297	26.63312	23.01088
H	32.85222	27.10188	24.44852
H	32.99857	24.61471	23.78713
H	29.61243	23.70378	24.39141
H	29.92857	27.24740	23.09420
H	25.11819	28.09239	22.87976
H	23.63225	27.10576	23.05609

H	25.16519	22.85889	23.61988	H	30.32975	23.89711	11.72889
H	31.18396	25.66304	21.56714	H	27.33412	23.12814	9.69425
H	31.17488	22.83450	22.77161	H	17.60099	17.31710	19.75028
H	29.94709	23.52795	21.65563	H	18.39330	17.81301	21.56054
H	33.62596	23.28558	17.84733	H	18.59753	17.90997	17.28364
H	32.92762	22.81810	19.43640	H	23.34440	17.73233	20.70504
H	31.86751	21.58471	17.62835	H	27.85338	30.26153	16.28742
H	28.56169	22.49061	17.67699	H	18.89225	19.01223	19.53623
H	30.23475	25.41054	19.11828	H	20.75594	16.78451	20.55503
H	28.88023	27.25769	20.18519	H	20.83321	17.16545	18.02847
H	26.53548	28.03083	20.40122	H	20.66346	19.84390	20.93403
H	25.10635	26.95978	20.15565	H	20.70424	20.20346	18.51223
H	31.92094	23.83931	15.96036	H	22.79383	19.65417	22.05110
H	29.17772	24.08971	15.92350	H	21.68631	18.49129	22.85609
H	30.19822	25.03377	17.06225	H	21.11831	21.82448	17.39081
H	28.19133	21.45640	13.63008	H	24.68177	19.38477	16.81691
H	27.27331	22.88184	14.29149	H	26.03624	21.50144	16.39414
H	26.66926	22.37357	11.91922	H	22.34660	26.06066	17.10895
H	28.05429	25.66276	10.68432	H	27.05285	25.82791	16.81612
H	30.91250	25.23967	13.14772	H	23.18368	27.81279	16.79398
H	24.71997	29.11803	13.94457	H	28.18102	28.03233	16.88600
H	24.57388	27.94163	12.73758	H	24.32667	30.00369	16.73303
H	29.29245	21.79814	11.27166	H	26.79492	30.02617	14.45925
H	29.83060	24.20070	10.02542	H	24.79683	24.51678	20.03905

H	23.30634	24.70664	23.36626

Unrelaxed-DNA fragment

O	20.93323	27.49309	17.45590
C	19.74242	27.95763	16.85476
C	19.39047	27.23022	15.56402
O	20.45766	27.35209	14.60988
C	20.27883	26.29917	13.67475
N	21.51931	26.10970	12.92844
C	22.68891	25.98602	13.61220
C	23.86525	25.68449	12.97421
C	23.76958	25.47738	11.54769
N	24.87348	25.09590	10.86371
N	22.63895	25.65203	10.85970
C	21.46868	25.98107	11.48530
O	20.39466	26.15594	10.90645
C	19.06803	25.71000	15.68921
C	19.85396	25.08650	14.52261
O	17.67814	25.46105	15.53060
P	16.71785	25.30691	16.91396
O	15.33411	24.96062	16.42524
O	17.07257	26.39857	17.91702
O	17.45585	23.94139	17.60146
C	17.34457	22.66490	17.02258
C	17.80906	21.62490	18.04392
O	19.13284	21.95142	18.52121
C	19.05232	22.55788	19.82209
N	19.80844	23.79441	19.86477
C	19.42330	25.10038	19.58138
N	20.44377	25.94129	19.63294
C	21.55029	25.16781	19.94091
C	22.94378	25.50123	20.02766
O	23.45556	26.63764	19.98102
N	23.74297	24.34719	20.13821
C	23.27632	23.05144	20.20293
N	24.20162	22.06256	20.21756
N	21.97225	22.74474	20.23597
C	21.18120	23.81865	20.05940
C	16.97224	21.54394	19.33084
C	17.56473	22.67480	20.17165
O	17.27308	20.28668	19.95950
P	16.11751	19.43449	20.80678
O	16.84640	18.10230	21.12970
O	14.77985	19.48912	20.12612
O	16.00171	20.32384	22.22748
C	16.76495	20.01673	23.37748
C	18.25819	20.24809	23.26247
O	18.58811	21.63167	23.09524
C	19.98621	21.66873	23.31671

N	20.42557	23.06272	23.35384
C	19.55820	24.11696	23.16770
C	19.98154	25.41499	23.07381
C	19.06984	26.57584	22.81929
C	21.41535	25.67793	23.18163
O	21.90124	26.82464	23.14545
N	22.22475	24.55952	23.34160
C	21.81980	23.24751	23.41969
O	22.59040	22.29580	23.55428
C	19.06844	19.76969	24.50265
C	20.19538	20.84102	24.59334
O	19.49289	18.43313	24.41602
O	32.03364	27.65275	22.64627
C	32.77808	26.73357	23.39877
C	32.19984	25.31255	23.43178
O	31.05876	25.20310	24.30776
C	30.03573	24.44635	23.68114
N	28.91646	25.35113	23.37114
C	28.96056	26.72349	23.17110
N	27.75881	27.27529	23.07022
C	26.88791	26.21473	23.20978
C	25.46965	26.13560	23.22708
N	24.67016	27.21175	23.12168
N	24.92552	24.89972	23.38133
C	25.71502	23.81255	23.52551
N	27.05071	23.77517	23.55854
C	27.58081	25.00133	23.39100
C	31.70825	24.81881	22.06451
C	30.67034	23.77156	22.45575
O	32.78283	24.37889	21.27386
P	33.27528	25.41046	20.00471
O	34.75113	25.17989	19.81621
O	32.61658	26.77612	20.23452
O	32.39051	24.71299	18.75506
C	32.71417	23.35371	18.48493
C	31.55915	22.64262	17.79474
O	30.40139	22.66710	18.64564
C	29.29875	23.26504	17.97164
N	28.56501	24.15413	18.88248
C	29.14928	25.32308	19.29450
C	28.41096	26.31638	19.87627
C	26.98843	26.10250	19.95606
N	26.14623	27.10673	20.23381
N	26.46826	24.87924	19.73912
C	27.22370	23.85175	19.25725
O	26.78227	22.69747	19.11630
C	31.13359	23.20549	16.42021
C	29.88378	24.01384	16.76841

O	30.85800	22.09405	15.55836
P	30.72390	22.37361	13.91326
O	30.51887	21.02695	13.24866
O	31.75475	23.40839	13.46711
O	29.25783	23.23464	13.87389
C	28.05442	22.55965	13.56872
C	27.61317	22.91132	12.15656
O	27.31750	24.33192	12.03394
C	28.44953	25.04337	11.52142
N	28.96304	26.00645	12.48657
C	30.18060	26.07079	13.17842
N	30.28361	27.15176	13.92564
C	29.09068	27.82396	13.75232
C	28.57684	29.01194	14.36502
O	29.09389	29.77695	15.20549
N	27.24458	29.26536	13.93424
C	26.45970	28.42195	13.17836
N	25.12389	28.74128	13.07840
N	26.94973	27.36252	12.55722
C	28.24103	27.11444	12.87488
C	28.65040	22.67301	11.04373
C	29.46055	23.98477	11.04664
O	28.01203	22.43105	9.78431
H	20.71523	27.09295	18.35316

H	18.85734	27.82039	17.51680
H	19.84450	29.04599	16.62038
H	18.46084	27.69123	15.14694
H	19.51334	26.55075	12.90799
H	22.62580	26.15882	14.69586
H	24.80725	25.55906	13.52502
H	24.75366	24.95146	9.86258
H	25.77139	24.90077	11.34009
H	19.43162	25.31194	16.66019
H	20.75220	24.55412	14.89198
H	19.22088	24.37351	13.96275
H	17.99121	22.58138	16.11569
H	16.29555	22.45641	16.70501
H	17.87397	20.62550	17.56313
H	19.57491	21.87753	20.52563
H	18.40594	25.38769	19.26422
H	25.16952	22.26002	19.88136
H	23.81926	21.12818	20.06201
H	15.88066	21.62505	19.15003
H	17.38038	22.51220	21.24736
H	17.15364	23.64140	19.83242
H	16.64368	18.94695	23.66698
H	16.36570	20.65007	24.20182
H	18.64325	19.68547	22.38635

H	20.53196	21.19188	22.46787
H	18.50250	23.82828	23.07998
H	18.01175	26.25528	22.75703
H	19.34520	27.06141	21.85998
H	19.17312	27.34709	23.61180
H	18.42662	19.85210	25.41259
H	20.06363	21.49946	25.47567
H	21.19910	20.38517	24.63835
H	19.54078	18.16013	23.45705
H	32.22999	27.42098	21.67354
H	33.82297	26.63312	23.01088
H	32.85222	27.10188	24.44852
H	32.99857	24.61471	23.78713
H	29.61243	23.70378	24.39141
H	29.92857	27.24740	23.09420
H	25.11819	28.09239	22.87976
H	23.63225	27.10576	23.05609
H	25.16519	22.85889	23.61988
H	31.18396	25.66304	21.56714
H	31.17488	22.83450	22.77161
H	29.94709	23.52795	21.65563
H	33.62596	23.28558	17.84733
H	32.92762	22.81810	19.43640
H	31.86751	21.58471	17.62835
H	28.56169	22.49061	17.67699
H	30.23475	25.41054	19.11828
H	28.88023	27.25769	20.18519
H	26.53548	28.03083	20.40122
H	25.10635	26.95978	20.15565
H	31.92094	23.83931	15.96036
H	29.17772	24.08971	15.92350
H	30.19822	25.03377	17.06225
H	28.19133	21.45640	13.63008
H	27.27331	22.88184	14.29149
H	26.66926	22.37357	11.91922
H	28.05429	25.66276	10.68432
H	30.91250	25.23967	13.14772
H	24.71997	29.11803	13.94457
H	24.57388	27.94163	12.73758
H	29.29245	21.79814	11.27166
H	29.83060	24.20070	10.02542
H	30.32975	23.89711	11.72889
H	27.33412	23.12814	9.69425
H	26.79492	30.02617	14.45925
H	24.79683	24.51678	20.03905
H	23.30634	24.70664	23.36626

Unrelaxed-DZN

O	22.00661	18.97328	19.58715

O	22.17862	19.28196	17.34824	C	26.44648	26.73980	16.81305
O	18.35043	17.05420	19.14153	C	24.28507	27.82805	16.76510
O	19.36618	17.67049	21.81490	C	27.08314	27.98308	16.84274
O	19.43027	18.37590	17.03963	C	24.92029	29.07594	16.75065
O	23.08708	17.60060	21.64099	C	26.32941	29.16973	16.75975
O	22.31329	24.05470	17.00550	H	17.60099	17.31710	19.75028
O	26.31363	23.99694	16.09926	H	18.39330	17.81301	21.56054
O	26.90818	30.39958	16.61244	H	18.59753	17.90997	17.28364
C	19.36450	18.01251	19.40307	H	23.34440	17.73233	20.70504
C	20.19139	17.74076	20.65818	H	27.85338	30.26153	16.28742
C	20.24904	18.11233	18.16166	H	18.89225	19.01223	19.53623
C	21.21987	18.88756	20.78410	H	20.75594	16.78451	20.55503
C	21.25110	19.23965	18.39890	H	20.83321	17.16545	18.02847
C	22.23147	18.69820	21.91223	H	20.66346	19.84390	20.93403
C	22.83391	20.46938	17.12355	H	20.70424	20.20346	18.51223
C	22.18654	21.70898	17.15603	H	22.79383	19.65417	22.05110
C	24.21681	20.38085	16.82533	H	21.68631	18.49129	22.85609
C	22.96234	22.85482	16.92971	H	21.11831	21.82448	17.39081
C	24.34249	22.80682	16.65378	H	24.68177	19.38477	16.81691
C	24.95564	21.53454	16.59229	H	26.03624	21.50144	16.39414
C	25.12578	24.04325	16.46716	H	22.34660	26.06066	17.10895
C	24.38146	25.29402	16.74637	H	27.05285	25.82791	16.81612
C	23.02399	25.20208	16.95872	H	23.18368	27.81279	16.79398
C	25.03658	26.62384	16.75981	H	28.18102	28.03233	16.88600

H	24.32667	30.00369	16.73303

^{2}[DNA-DZN]$^{3-}$

O	20.98695	27.46100	17.52049
C	19.82100	27.97228	16.90632
C	19.51004	27.29601	15.57999
O	20.61279	27.41475	14.67563
C	20.38013	26.44980	13.65614
N	21.60997	26.23369	12.90946
C	22.77036	26.00184	13.58320
C	23.92529	25.66769	12.92523
C	23.81731	25.54407	11.48793
N	24.89553	25.13110	10.78473
N	22.70023	25.82558	10.81431
C	21.55083	26.18491	11.45842
O	20.48614	26.44898	10.89960
C	19.16601	25.78038	15.64992
C	19.85458	25.20419	14.39999
O	17.75770	25.57684	15.59383
P	16.92016	25.24142	17.00426
O	15.51323	24.86873	16.59972
O	17.24694	26.29129	18.07057
O	17.77029	23.89700	17.54703
C	17.56257	22.60677	17.00738
C	17.94640	21.57372	18.06800
O	19.24866	21.89502	18.60961
C	19.10401	22.53368	19.88284
N	19.86227	23.77975	19.91509
C	19.47240	25.08335	19.68052
N	20.50070	25.93447	19.70944
C	21.60641	25.16470	19.94758
C	23.00807	25.50345	20.03219
O	23.49841	26.64379	20.01904
N	23.81567	24.35568	20.10554
C	23.35332	23.06319	20.14010
N	24.25306	22.06978	20.08029
N	22.03801	22.74435	20.19615
C	21.24072	23.80127	20.05877
C	17.05286	21.51404	19.31652
C	17.60390	22.66372	20.16431
O	17.34290	20.27695	19.97611
P	16.15508	19.43358	20.80340
O	16.86201	18.09355	21.13187
O	14.83900	19.52510	20.08869
O	16.03851	20.32405	22.22060
C	16.78147	19.99893	23.38028
C	18.27790	20.21531	23.27636
O	18.61115	21.59716	23.08244
C	19.99857	21.65053	23.34917
N	20.41953	23.05199	23.36458
C	19.52367	24.09095	23.24416
C	19.91564	25.39914	23.14701
C	18.96856	26.54421	22.95893
C	21.34735	25.69006	23.19245
O	21.80855	26.84595	23.13878
N	22.18552	24.58599	23.30065
C	21.81030	23.26448	23.38104
O	22.60536	22.32702	23.46351
C	19.08031	19.75292	24.52890
C	20.17438	20.85559	24.64881

O	19.55647	18.43515	24.43533	C	27.04264	26.09415	19.97279
O	31.86817	27.80121	22.47156	N	26.23604	27.11705	20.28365
C	32.68778	26.92154	23.19730	N	26.48281	24.89451	19.70887
C	32.12292	25.50431	23.34594	C	27.20620	23.86323	19.18764
O	31.03631	25.44975	24.29761	O	26.72641	22.72470	19.01715
C	29.98561	24.65303	23.78839	C	31.10515	23.13857	16.36830
N	28.84528	25.53461	23.46905	C	29.87025	23.97463	16.70410
C	28.86560	26.89905	23.22564	O	30.80349	22.02662	15.51271
N	27.65299	27.42237	23.08918	P	30.65851	22.30580	13.87668
C	26.80292	26.34893	23.25070	O	30.47885	20.96124	13.19598
C	25.38754	26.23829	23.23048	O	31.69060	23.34045	13.42507
N	24.56643	27.29225	23.08276	O	29.19036	23.14819	13.83136
N	24.86782	24.99018	23.37352	C	27.99998	22.48071	13.45610
C	25.67825	23.92392	23.56122	C	27.59609	22.89275	12.04908
N	27.01135	23.91971	23.64213	O	27.29909	24.31641	11.98486
C	27.51853	25.15563	23.47296	C	28.43266	25.05260	11.50828
C	31.53847	24.93033	22.04762	N	28.92500	25.98554	12.51444
C	30.54395	23.90069	22.57194	C	30.12375	26.02237	13.23920
O	32.54470	24.44279	21.19709	N	30.21172	27.07554	14.02667
P	33.04977	25.46242	19.92844	C	29.03035	27.76180	13.83990
O	34.54759	25.33337	19.82698	C	28.51473	28.94182	14.46357
O	32.28826	26.78722	20.06346	O	29.02301	29.67982	15.33383
O	32.29243	24.67782	18.65175	N	27.19772	29.22331	14.00441
C	32.64116	23.31258	18.45308	C	26.43250	28.42139	13.18462
C	31.51202	22.57615	17.74643	N	25.11517	28.77744	13.01340
O	30.33417	22.59246	18.57187	N	26.92684	27.36503	12.56238
C	29.25963	23.22922	17.89661	C	28.20102	27.08701	12.91622
N	28.54504	24.13520	18.81216	C	28.65856	22.70075	10.95029
C	29.16544	25.27396	19.25278	C	29.45809	24.01741	11.01590
C	28.46723	26.26478	19.88662	O	28.05517	22.48752	9.67258

O	22.02008	18.90503	19.61188
O	22.20395	19.17151	17.37069
O	18.35788	16.98669	19.17268
O	19.36953	17.62861	21.84496
O	19.44750	18.29791	17.06688
O	23.08377	17.54136	21.69584
O	22.28648	23.94118	16.99138
O	26.25337	23.91489	15.96586
O	26.91244	30.24916	16.78575
C	19.37487	17.93927	19.43042
C	20.19810	17.67767	20.69072
C	20.26177	18.02383	18.19012
C	21.22769	18.82485	20.80846
C	21.26804	19.14605	18.42000
C	22.23557	18.64765	21.94143
C	22.83503	20.35604	17.10345
C	22.18179	21.59406	17.14725
C	24.20695	20.27532	16.75132
C	22.94227	22.74156	16.89277
C	24.31330	22.70211	16.57610
C	24.93002	21.43221	16.49009
C	25.08420	23.94229	16.38626
C	24.35014	25.18662	16.73506
C	22.99088	25.09024	16.95928
C	25.01450	26.50192	16.80345
C	26.43058	26.60041	16.82065
C	24.27259	27.71434	16.89607
C	27.07845	27.83067	16.89246
C	24.91829	28.95089	16.92456
C	26.33109	29.03085	16.88810
H	20.75738	27.11197	18.43134
H	18.91849	27.82053	17.54108
H	19.94161	29.06719	16.72233
H	18.60468	27.78616	15.14210
H	19.64438	26.81488	12.90631
H	22.71837	26.12167	14.67436
H	24.86373	25.47312	13.46124
H	24.77312	25.04676	9.77683
H	25.77653	24.86217	11.25758
H	19.58824	25.32642	16.57069
H	20.70029	24.54543	14.67881
H	19.13731	24.62146	13.79302
H	18.20899	22.45475	16.11125
H	16.50258	22.47315	16.69300
H	18.02674	20.56748	17.60472
H	19.59363	21.87962	20.63198
H	18.44551	25.38868	19.40919
H	25.23692	22.26490	19.75627
H	23.85963	21.12943	19.99762
H	15.96847	21.58472	19.09143
H	17.37188	22.51329	21.23261
H	17.20512	23.62724	19.80228
H	16.64119	18.93001	23.66207
H	16.37963	20.63304	24.20192
H	18.66695	19.63928	22.41120
H	20.58008	21.16169	22.53148
H	18.47092	23.78003	23.20852
H	17.91573	26.20250	22.93574
H	19.19219	27.06612	22.00505
H	19.08817	27.29822	23.76496

H	18.42331	19.80804	25.42970	H	28.04425	25.69766	10.68841
H	19.98334	21.52690	25.51026	H	30.86012	25.19841	13.18600
H	21.18524	20.42366	24.74836	H	24.66398	29.19214	13.83470
H	19.56185	18.14271	23.48170	H	24.56049	28.00480	12.62551
H	31.99859	27.54014	21.50025	H	29.30168	21.82303	11.16198
H	33.69431	26.80925	22.72315	H	29.85143	24.27204	10.01299
H	32.85033	27.33992	24.21674	H	30.31311	23.90904	11.71229
H	32.94325	24.82902	23.69243	H	27.40689	23.20772	9.55395
H	29.60690	23.96243	24.57215	H	17.60437	17.26076	19.77077
H	29.82454	27.43592	23.12690	H	18.39934	17.77463	21.58444
H	24.99398	28.20488	22.94624	H	18.61654	17.82147	17.29776
H	23.52981	27.16805	23.04016	H	23.35136	17.64788	20.76009
H	25.14842	22.95855	23.65189	H	27.85051	30.12523	16.41352
H	30.97188	25.74387	21.54647	H	18.90972	18.94337	19.55527
H	31.07787	22.98920	22.91203	H	20.75913	16.71836	20.60232
H	29.76682	23.60342	21.84350	H	20.83512	17.07099	18.05905
H	33.57524	23.23126	17.85107	H	20.66709	19.78076	20.94920
H	32.81970	22.81552	19.43153	H	20.72230	20.11184	18.51331
H	31.83197	21.52060	17.59477	H	22.80342	19.60355	22.06534
H	28.50043	22.48160	17.58984	H	21.68640	18.46507	22.88787
H	30.24740	25.34712	19.05395	H	21.12354	21.70857	17.42159
H	28.97305	27.18339	20.20688	H	24.67733	19.28222	16.72778
H	26.65379	27.97296	20.64331	H	26.00308	21.40219	16.25375
H	25.20040	26.99465	20.24903	H	22.31600	25.94918	17.12285
H	31.90835	23.75006	15.90694	H	27.02712	25.68444	16.76002
H	29.17348	24.06369	15.85236	H	23.17201	27.70875	16.94266
H	30.20472	24.98640	17.00293	H	28.17683	27.87078	16.91552
H	28.14037	21.37673	13.47582	H	24.33699	29.88482	16.96656
H	27.19864	22.76995	14.16990	H	26.75070	30.00016	14.50417
H	26.66090	22.36223	11.76628	H	24.88388	24.54461	20.00142

H	23.26700	24.75938	23.32679

P2

O	20.35936	27.01885	17.14226
C	19.08153	27.30083	16.60438
C	18.89882	26.75997	15.19321
O	19.89523	27.30438	14.32385
C	19.94525	26.46875	13.17185
N	21.21157	26.69607	12.49227
C	22.37400	26.56445	13.20608
C	23.59386	26.69810	12.61175
C	23.60163	27.02827	11.20096
N	24.78631	27.17293	10.58685
N	22.45256	27.19221	10.51010
C	21.24650	26.98395	11.08556
O	20.16526	26.97665	10.45386
C	18.96694	25.21457	15.03263
C	19.73644	25.03341	13.70690
O	17.67473	24.63027	14.94356
P	16.96134	23.97320	16.32414
O	15.77860	23.16211	15.85498
O	16.92095	25.01903	17.43740
O	18.21304	22.94226	16.82364
C	18.27440	21.60630	16.37592
C	18.55220	20.67456	17.55611
O	19.67311	21.16332	18.32795
C	19.20875	21.82729	19.51476
N	19.79454	23.15139	19.59870
C	19.28930	24.37794	19.19148
N	20.20763	25.33027	19.23415
C	21.37100	24.71156	19.66368
C	22.72188	25.18473	19.72651
O	23.12918	26.34286	19.49289
N	23.62330	24.14708	20.03290
C	23.28555	22.82077	20.23514
N	24.28955	21.94752	20.45531
N	22.02014	22.38165	20.19999
C	21.13708	23.34390	19.87954
C	17.44935	20.53634	18.61628
C	17.67811	21.77203	19.49051
O	17.78913	19.36522	19.37411
P	16.67005	18.41667	20.15821
O	17.55085	17.25453	20.69261
O	15.44151	18.19508	19.32436
O	16.23950	19.39972	21.45530
C	16.80272	19.25378	22.74307
C	18.26283	19.63282	22.87525
O	18.50832	21.03872	22.69779
C	19.84463	21.17341	23.14830
N	20.23732	22.57900	23.14432
C	19.36627	23.60259	22.84429
C	19.77499	24.90301	22.70999
C	18.86070	26.03187	22.34369
C	21.19985	25.19661	22.87299
O	21.68543	26.33679	22.74960
N	22.00710	24.10971	23.18945
C	21.61516	22.80107	23.33060
O	22.38283	21.87871	23.61204
C	18.88794	19.26322	24.25536

C	19.86578	20.44807	24.49899
O	19.45724	17.97952	24.28243
O	31.82453	26.66518	21.73191
C	32.59794	25.90799	22.63315
C	31.97382	24.56855	23.04736
O	30.89863	24.72406	23.99966
C	29.81086	23.88626	23.64478
N	28.70210	24.73765	23.18602
C	28.78645	26.00495	22.62608
N	27.60176	26.56051	22.41813
C	26.69942	25.61299	22.85150
C	25.27829	25.58894	22.88739
N	24.51316	26.61163	22.46468
N	24.69754	24.46765	23.39275
C	25.45511	23.43499	23.82468
N	26.78858	23.35268	23.84455
C	27.35659	24.46430	23.33865
C	31.36223	23.78180	21.87872
C	30.32708	22.90922	22.57741
O	32.34838	23.09536	21.14788
P	33.06790	23.94685	19.86572
O	34.53092	24.16131	20.16895
O	32.14816	25.14588	19.53651
O	32.90946	22.80479	18.65930
C	32.26969	21.54852	18.82791
C	30.81691	21.53565	18.36927
O	30.00670	22.34052	19.25605
C	29.19536	23.15608	18.40869
N	28.51896	24.24294	19.10540
C	28.94765	25.54024	19.00893

C	28.07683	26.57946	19.21353
C	26.70040	26.25421	19.48513
N	25.73984	27.20312	19.53540
N	26.32673	24.98425	19.70896
C	27.20929	23.95364	19.59740
O	26.91600	22.77446	19.85009
C	30.58261	22.09497	16.93885
C	30.14390	23.52544	17.27259
O	29.49696	21.37174	16.36632
P	29.21494	21.35063	14.69563
O	27.88508	20.61662	14.57831
O	30.49136	21.04677	13.95654
O	28.90168	23.01459	14.40114
C	27.76565	23.56535	15.01275
C	26.66704	23.92352	14.00128
O	26.57672	25.53326	11.43115
C	25.95800	24.40940	11.05725
N	25.80530	24.34637	9.52086
C	26.75314	24.73516	8.59315
N	26.25502	25.09007	7.41797
C	24.88629	24.96177	7.58410
C	23.78804	25.33132	6.72138
O	23.76706	25.75613	5.56249
N	22.55017	25.15464	7.42552
C	22.37398	24.68085	8.70828
N	21.06495	24.58286	9.15459
N	23.38266	24.29089	9.46153
C	24.59489	24.48760	8.88538
C	26.14453	22.80356	13.03412
C	26.58748	23.10493	11.58979

O	24.73169	22.65097	13.11239	C	25.25768	29.36031	16.45420
O	22.77335	18.81311	19.81595	H	20.24934	26.54427	18.02747
O	23.20551	19.10241	17.60930	H	18.26525	26.84220	17.20730
O	19.47744	16.41700	18.95822	H	18.92084	28.40711	16.57197
O	20.00177	17.26286	21.71363	H	17.87745	27.05146	14.83898
O	20.69785	17.80412	16.96676	H	19.16300	26.74232	12.42994
O	23.68159	17.58130	22.06287	H	22.24405	26.36319	14.27665
O	22.22460	23.68730	16.78897	H	24.52932	26.57884	13.17210
O	25.61277	24.58810	14.75896	H	24.69934	27.28434	9.57136
O	25.64116	30.66965	16.57281	H	25.66227	26.57665	10.98781
C	20.30908	17.50953	19.31312	H	19.53298	24.76453	15.87559
C	20.97260	17.38486	20.68146	H	20.71567	24.54076	13.86996
C	21.35419	17.68408	18.21245	H	19.14531	24.40900	13.01100
C	21.83782	18.64753	20.89983	H	19.10687	21.49066	15.64237
C	22.15181	18.94607	18.53747	H	17.32252	21.31709	15.87442
C	22.69198	18.59169	22.16370	H	18.83894	19.67176	17.17440
C	23.48246	20.32074	17.07824	H	19.61242	21.26223	20.37847
C	22.62984	21.42741	17.14476	H	18.26705	24.52345	18.79085
C	24.72693	20.43565	16.39906	H	25.28238	22.22247	20.29394
C	23.08986	22.64212	16.63280	H	24.02197	20.96153	20.42356
C	24.34847	22.81854	15.98114	H	16.42628	20.45220	18.19536
C	25.13871	21.63361	15.84345	H	17.25649	21.63133	20.50151
C	24.68371	24.16324	15.58232	H	17.24261	22.66574	19.00771
C	23.90944	25.25572	16.14763	H	16.73738	18.19718	23.09300
C	22.65551	24.93785	16.63402	H	16.19815	19.88801	23.43057
C	24.37361	26.66714	16.18938	H	18.84384	19.08343	22.10584
C	25.74806	26.99570	16.15658	H	20.54371	20.66094	22.44701
C	23.45296	27.72926	16.38335	H	18.32533	23.28451	22.69346
C	26.18611	28.31750	16.28781	H	17.81346	25.68749	22.23847
C	23.88642	29.05078	16.51047	H	19.17993	26.47646	21.37842

H	18.90363	26.84250	23.10195	H	24.85332	24.37857	11.32095
H	18.09492	19.28293	25.04018	H	27.81463	24.77364	8.87123
H	19.49006	21.13706	25.28304	H	20.59306	25.51024	9.23036
H	20.87632	20.10246	24.77710	H	21.08525	24.19582	10.10440
H	19.77193	17.73877	23.36794	H	26.59644	21.83987	13.35301
H	31.94006	26.21148	20.83368	H	26.27884	22.24209	10.96391
H	33.59902	25.66199	22.20152	H	27.69408	23.18689	11.55320
H	32.76366	26.51328	23.55404	H	24.33509	23.32933	12.52601
H	32.77606	23.93981	23.50726	H	18.62088	16.58039	19.44880
H	29.41813	23.35910	24.54097	H	19.06586	17.23058	21.31978
H	29.76670	26.44774	22.38050	H	19.90504	17.23458	17.09599
H	24.96839	27.31724	21.88457	H	24.07875	17.72280	21.17933
H	23.47456	26.54279	22.48837	H	26.60995	30.69571	16.46417
H	24.87746	22.56259	24.17907	H	19.69885	18.44131	19.32876
H	30.82987	24.49298	21.21292	H	21.64814	16.49768	20.70600
H	30.82372	22.04909	23.07407	H	22.06698	16.82002	18.21742
H	29.53751	22.53136	21.90162	H	21.16444	19.53640	20.93645
H	32.82534	20.81118	18.20479	H	21.46636	19.81959	18.54055
H	32.31419	21.21783	19.88653	H	23.12596	19.60267	22.35443
H	30.45193	20.48218	18.38298	H	22.03466	18.34870	23.02426
H	28.36658	22.52966	18.01297	H	21.62647	21.41087	17.59324
H	30.02013	25.66779	18.78633	H	25.38264	19.55743	16.31986
H	28.41982	27.61997	19.16284	H	26.11803	21.60699	15.34133
H	25.95721	28.11457	19.13480	H	21.87597	25.67257	16.89949
H	24.73423	26.88347	19.51023	H	26.50551	26.21186	16.04792
H	31.48316	22.03680	16.29162	H	22.36850	27.54448	16.45760
H	29.69023	24.03443	16.40870	H	27.26761	28.53501	16.25974
H	31.00247	24.10454	17.66734	H	23.15767	29.86179	16.65214
H	27.37017	22.90442	15.81433	H	21.72090	25.40739	6.88358
H	28.04742	24.52252	15.51499	H	24.64832	24.42898	19.99206

H	23.08142	24.27752	23.29494
H	27.01782	24.76270	13.36471

R0-H_2O

O	20.56184	27.37716	17.71524
C	19.41343	27.90391	17.10443
C	19.22680	27.38432	15.68078
O	20.44432	27.51210	14.93399
C	20.34170	26.70514	13.75612
N	21.65633	26.17641	13.40782
C	22.19133	25.11139	14.08259
C	23.44695	24.64516	13.81692
C	24.21833	25.35401	12.82247
N	25.48170	24.99612	12.56854
N	23.67135	26.37414	12.12830
C	22.42443	26.85959	12.39707
O	21.94273	27.84450	11.82407
C	18.83368	25.90256	15.51163
C	19.25990	25.64260	14.05441
O	17.44868	25.65532	15.64665
P	16.79164	25.03649	17.07877
O	15.46879	24.41576	16.69781
O	16.99317	26.03168	18.21700
O	17.93051	23.82874	17.41043
C	17.77386	22.52096	16.90623
C	18.04452	21.50117	18.01490
O	19.26181	21.83822	18.71030
C	18.95594	22.43396	19.98509
N	19.70250	23.66059	20.15177
C	19.35166	24.96750	19.84409
N	20.37987	25.79341	19.93107
C	21.46402	25.00426	20.28730
C	22.86932	25.29151	20.36631
O	23.42375	26.40310	20.26222
N	23.63418	24.11678	20.52677
C	23.13154	22.83620	20.63284
N	24.01721	21.82005	20.71913
N	21.81748	22.57317	20.63304
C	21.06321	23.66446	20.40937
C	17.01052	21.40894	19.14791
C	17.43291	22.55807	20.06501
O	17.28075	20.16198	19.80567
P	16.16509	19.31562	20.70134
O	16.96658	18.04444	21.09250
O	14.82764	19.26002	20.02026
O	16.00746	20.28631	22.06700
C	16.67200	20.01044	23.28357
C	18.16350	20.27927	23.30299
O	18.48455	21.67149	23.17776
C	19.85731	21.72239	23.53246
N	20.28758	23.11672	23.61746
C	19.43351	24.17299	23.38797
C	19.86768	25.46837	23.30471
C	18.97353	26.63070	23.00030
C	21.30129	25.72470	23.44442
O	21.80177	26.86256	23.37689
N	22.09603	24.60492	23.66844
C	21.67797	23.29789	23.75357
O	22.43485	22.34412	23.94467
C	18.86265	19.80054	24.60991
C	19.95502	20.88899	24.81765

O	19.31640	18.47195	24.54998
O	31.95681	27.40709	22.32219
C	32.68984	26.49122	23.08872
C	32.08230	25.08428	23.16398
O	30.96051	25.02602	24.06927
C	29.90846	24.26947	23.49153
N	28.79634	25.18082	23.17909
C	28.86593	26.52542	22.83635
N	27.67840	27.11066	22.77679
C	26.78930	26.10543	23.09491
C	25.37658	26.08782	23.24280
N	24.60684	27.18189	23.09269
N	24.80856	24.90130	23.58855
C	25.57398	23.80355	23.77854
N	26.90448	23.71252	23.69368
C	27.45725	24.89031	23.34764
C	31.55046	24.55960	21.82026
C	30.50177	23.54786	22.27361
O	32.59336	24.05890	21.02350
P	33.15212	25.06036	19.76221
O	34.61919	24.75512	19.60371
O	32.55706	26.45702	19.96529
O	32.26700	24.40487	18.48834
C	32.60420	23.06877	18.14263
C	31.35575	22.24427	17.87503
O	30.46091	22.30369	18.99231
C	29.10939	22.30021	18.56897
N	28.42032	23.49298	19.10977
C	29.00891	24.72480	19.03951
C	28.31691	25.85188	19.39408
C	26.93840	25.68776	19.77524
N	26.13379	26.74641	19.94993
N	26.41350	24.45975	19.96388
C	27.11123	23.33751	19.64630
O	26.65064	22.18632	19.79232
C	30.54384	22.64740	16.63014
C	29.11780	22.23199	17.02919
O	31.03576	21.91731	15.51243
P	31.20200	22.64473	14.01616
O	31.70078	21.57381	13.06708
O	31.81486	24.02768	14.16631
O	29.55171	22.94130	13.64536
C	28.90381	22.10995	12.69657
C	28.79376	22.81707	11.34617
O	28.21068	24.12913	11.50285
C	29.17224	25.15055	11.24902
N	29.18936	26.18498	12.27510
C	30.17039	26.47506	13.24101
N	29.98742	27.64415	13.81682
C	28.83894	28.15138	13.24302
C	28.11866	29.36169	13.48848
O	28.37981	30.30697	14.26520
N	26.91607	29.40266	12.73343
C	26.42765	28.41274	11.90149
N	25.18100	28.56613	11.37543
N	27.14866	27.32902	11.60573
C	28.30476	27.24566	12.30088
C	30.11441	23.05113	10.58129
C	30.52168	24.44695	11.05642
O	29.91418	22.98995	9.16233

O	22.24930	19.17191	19.99318	H	20.37792	26.97116	18.61412
O	22.57420	19.61199	17.78656	H	18.47716	27.63663	17.64770
O	18.69781	17.17537	19.17398	H	19.48418	29.02058	17.04912
O	19.45994	17.73293	21.94544	H	18.40777	27.98674	15.20633
O	19.92209	18.57664	17.21311	H	20.07811	27.34988	12.89125
O	23.17121	17.70149	22.07812	H	21.55676	24.66297	14.86004
O	22.28644	24.38458	17.39695	H	23.87364	23.79995	14.37032
O	26.33552	24.69220	16.78452	H	26.03554	25.60101	11.94375
O	25.91199	30.86042	14.98818	H	26.01937	24.38076	13.22648
C	19.65288	18.15382	19.55479	H	19.42406	25.28892	16.22899
C	20.37653	17.85529	20.86402	H	19.59709	24.60518	13.86765
C	20.64301	18.32061	18.40215	H	18.36729	25.81811	13.42167
C	21.36783	19.01231	21.12045	H	18.51168	22.34221	16.08796
C	21.57391	19.47796	18.76047	H	16.74954	22.38088	16.49094
C	22.28506	18.78319	22.31913	H	18.19659	20.49799	17.56343
C	23.11163	20.85484	17.55538	H	19.33319	21.74141	20.76390
C	22.35966	22.03588	17.57410	H	18.34934	25.26404	19.48254
C	24.49503	20.88518	17.25019	H	25.01756	21.96873	20.45456
C	23.03748	23.24495	17.35310	H	23.60751	20.89395	20.56381
C	24.42602	23.31638	17.10840	H	15.95611	21.45458	18.80529
C	25.13838	22.09552	17.03396	H	17.06197	22.41354	21.09512
C	25.09068	24.61550	16.93327	H	17.07922	23.52219	19.65579
C	24.20085	25.77818	16.90879	H	16.55476	18.94034	23.57237
C	22.86566	25.58466	17.18243	H	16.18461	20.63893	24.06327
C	24.65905	27.12277	16.49157	H	18.63759	19.73098	22.46144
C	26.00479	27.53886	16.62496	H	20.48496	21.25971	22.73458
C	23.75218	28.02485	15.87887	H	18.38188	23.88670	23.25182
C	26.42905	28.79029	16.16345	H	17.91446	26.31791	22.91734
C	24.17314	29.27188	15.40783	H	19.27887	27.08948	22.03684
C	25.52093	29.66518	15.53149	H	19.06332	27.41965	23.77689

H	18.13540	19.86086	25.45505
H	19.72611	21.53786	25.68732
H	20.95816	20.45057	24.95603
H	19.46450	18.21492	23.59704
H	32.15411	27.14769	21.35551
H	33.72816	26.36128	22.69199
H	32.78260	26.88061	24.12964
H	32.87297	24.37688	23.51915
H	29.48655	23.55858	24.23421
H	29.84263	27.01039	22.66301
H	25.04591	27.98948	22.65522
H	23.56837	27.09865	23.10684
H	25.00554	22.88778	24.02137
H	31.03664	25.39785	21.30122
H	30.99945	22.61116	22.60060
H	29.75214	23.29413	21.50391
H	33.21569	23.05117	17.21101
H	33.20315	22.59474	18.94900
H	31.68082	21.18804	17.69946
H	28.55598	21.45305	19.02033
H	30.06482	24.74514	18.71701
H	28.79603	26.83724	19.34180
H	26.50985	27.67662	19.78472
H	25.10030	26.61966	20.10135
H	30.63529	23.73991	16.44560
H	28.98082	21.18079	16.70379
H	28.32245	22.83796	16.56103
H	29.46147	21.15691	12.56600
H	27.88301	21.90107	13.07943
H	28.11932	22.21367	10.69483

H	28.84365	25.68752	10.32599
H	30.94140	25.73002	13.51864
H	24.61210	29.29683	11.81140
H	24.62207	27.65733	11.36902
H	30.87685	22.29167	10.84444
H	31.16541	24.95690	10.31390
H	31.07551	24.37420	12.01444
H	29.18131	23.60556	8.96998
H	17.89229	17.38031	19.73062
H	18.50405	17.83056	21.61611
H	19.08946	18.07425	17.37137
H	23.51053	17.87218	21.17561
H	26.91812	30.83367	14.88203
H	19.13738	19.13339	19.67754
H	20.96443	16.91160	20.77507
H	21.27491	17.40046	18.30323
H	20.78783	19.95429	21.26267
H	20.98293	20.41134	18.88356
H	22.82505	19.73429	22.54592
H	21.66283	18.53442	23.20324
H	21.28003	22.05804	17.78275
H	25.04314	19.93295	17.23000
H	26.21048	22.13534	16.80744
H	22.10962	26.38835	17.24647
H	26.73500	26.85307	17.06977
H	22.69661	27.74960	15.73520
H	27.48676	29.07901	16.25499
H	23.45912	29.94384	14.90720
H	26.27076	30.13493	13.06482
H	24.68704	24.23944	20.41763

H	23.17897	24.74314	23.68174
O	26.99244	23.59621	14.46233
H	26.86908	24.06941	15.32824
H	27.95966	23.65722	14.27303

R0-O_2

O	20.70396	27.35598	17.45372
C	19.48699	27.77011	16.86675
C	19.18592	27.05963	15.55250
O	20.22370	27.34486	14.59951
C	20.30786	26.27044	13.68342
N	21.66572	26.25550	13.12325
C	22.72257	26.51867	13.93790
C	24.01317	26.43054	13.48272
C	24.17216	26.04092	12.10757
N	25.43017	25.97318	11.58322
N	23.14399	25.80593	11.29098
C	21.85138	25.88821	11.73856
O	20.85750	25.66634	11.04225
C	19.04244	25.51056	15.62812
C	19.94724	25.00635	14.48876
O	17.69886	25.09815	15.41387
P	16.69094	24.92700	16.76294
O	15.35492	24.47043	16.23200
O	16.92881	26.07703	17.73527
O	17.48031	23.64030	17.53534
C	17.36880	22.32005	17.06277
C	17.75038	21.36129	18.19106
O	19.04001	21.72105	18.73135
C	18.87769	22.41611	19.97868
N	19.64385	23.64585	19.98136
C	19.28966	24.93036	19.58843
N	20.32077	25.75935	19.61514
C	21.40549	24.99614	20.01790
C	22.80401	25.30855	20.11792
O	23.33983	26.42721	20.00041
N	23.57819	24.15086	20.33720
C	23.08337	22.87306	20.49899
N	23.97924	21.87111	20.63665
N	21.77148	22.59602	20.51072
C	21.00873	23.66567	20.22283
C	16.83494	21.37058	19.42609
C	17.37202	22.56444	20.21721
O	17.11495	20.16327	20.15230
P	15.97276	19.40110	21.09460
O	16.71463	18.11204	21.53964
O	14.62782	19.37978	20.42621
O	15.86943	20.43377	22.41703
C	16.59622	20.21840	23.61043
C	18.09452	20.43082	23.52569
O	18.44914	21.79704	23.27750
C	19.84065	21.83271	23.54730
N	20.30188	23.21954	23.51936
C	19.46011	24.27652	23.25100
C	19.91425	25.55661	23.08370
C	19.03283	26.71720	22.73775
C	21.35367	25.79634	23.19079
O	21.86615	26.92496	23.07575
N	22.13663	24.67421	23.44059
C	21.69913	23.38054	23.60046
O	22.44467	22.42275	23.81326

C	18.85366	20.02808	24.82398
C	19.99715	21.08245	24.87814
O	19.25162	18.68089	24.84946
O	32.02752	27.43025	22.29505
C	32.77164	26.44148	22.95091
C	32.12828	25.04970	22.95386
O	31.04056	24.96368	23.90005
C	29.95337	24.25911	23.32365
N	28.85034	25.20488	23.08836
C	28.93521	26.56304	22.80894
N	27.75258	27.15830	22.75062
C	26.85177	26.14520	23.00061
C	25.43444	26.12621	23.08913
N	24.67580	27.22755	22.93350
N	24.85012	24.93114	23.37017
C	25.60510	23.82221	23.54109
N	26.93678	23.72632	23.48794
C	27.50626	24.91521	23.21309
C	31.52452	24.62509	21.60368
C	30.48679	23.59661	22.04683
O	32.51025	24.16537	20.71638
P	33.04240	25.25097	19.51552
O	34.49267	24.91957	19.27053
O	32.50256	26.64151	19.86387
O	32.09086	24.75043	18.22041
C	32.43053	23.49331	17.64675
C	31.21377	22.58316	17.52106
O	30.47639	22.52728	18.75457
C	29.08664	22.43024	18.51126
N	28.38851	23.61769	19.07126
C	28.95175	24.85917	18.97951
C	28.24114	25.97710	19.32632
C	26.87167	25.79512	19.72382
N	26.06379	26.84869	19.92904
N	26.36078	24.55947	19.90368
C	27.07396	23.44554	19.58409
O	26.62073	22.28838	19.71053
C	30.18512	22.92960	16.42454
C	28.90685	22.30639	16.99185
O	30.52548	22.29115	15.19058
P	31.13008	23.11702	13.88852
O	31.35644	22.08188	12.80472
O	32.09354	24.22042	14.26709
O	29.70822	24.00244	13.38017
C	28.73193	23.35298	12.63519
C	28.74066	23.81862	11.18171
O	28.43337	25.22360	11.06520
C	29.64026	25.99319	11.03213
N	29.79548	26.84816	12.20638
C	30.77358	26.89223	13.21187
N	30.57461	27.85816	14.08197
C	29.39868	28.45568	13.68465
C	28.58978	29.44918	14.32208
O	28.80536	30.11043	15.35869
N	27.34073	29.59993	13.65410
C	26.93174	28.90250	12.53988
N	25.65056	29.11743	12.07500
N	27.71625	28.05606	11.89695
C	28.89027	27.83489	12.52513
C	30.07767	23.68818	10.42807

C	30.79388	24.99384	10.81806
O	29.84826	23.60008	9.01843
O	21.92449	18.92266	20.09118
O	22.14087	19.14154	17.84066
O	18.28751	16.96235	19.64953
O	19.22239	17.73194	22.31142
O	19.42410	18.16479	17.50824
O	22.94895	17.64127	22.23248
O	22.12694	23.88244	17.14893
O	26.20559	23.92848	16.71582
O	26.36628	30.41856	16.30737
C	19.29032	17.93895	19.88666
C	20.08087	17.73810	21.17667
C	20.21097	17.97835	18.66798
C	21.10709	18.89008	21.27138
C	21.19308	19.12993	18.87275
C	22.08880	18.75125	22.43272
C	22.76796	20.33653	17.56969
C	22.07362	21.54658	17.46612
C	24.16954	20.28557	17.37381
C	22.82024	22.70757	17.21588
C	24.21915	22.70062	17.05992
C	24.88065	21.45296	17.12565
C	24.97440	23.95686	16.88180
C	24.16146	25.19293	16.93813
C	22.79502	25.05095	17.03913
C	24.75218	26.55158	16.85382
C	26.14284	26.74027	16.66930
C	23.95213	27.72276	16.95033
C	26.70825	28.01319	16.53880

C	24.50680	28.99476	16.78216
C	25.88911	29.16065	16.54639
H	20.51906	26.91092	18.34037
H	18.61102	27.56111	17.52035
H	19.52692	28.86823	16.66244
H	18.20747	27.43470	15.16541
H	19.63231	26.40211	12.80934
H	22.46460	26.82968	14.95928
H	24.86198	26.61680	14.15294
H	25.52218	25.59575	10.64103
H	26.26517	25.94055	12.18471
H	19.41214	25.13312	16.60530
H	20.87027	24.54767	14.89216
H	19.41879	24.25306	13.87620
H	18.06328	22.14700	16.20536
H	16.33384	22.11055	16.70382
H	17.84307	20.32868	17.79234
H	19.34092	21.78426	20.76359
H	18.28687	25.20096	19.21582
H	24.97958	22.01487	20.36772
H	23.57827	20.93523	20.54847
H	15.75454	21.42676	19.17952
H	17.11576	22.48897	21.28840
H	16.98894	23.50223	19.77763
H	16.45889	19.17647	23.98210
H	16.17754	20.91854	24.36851
H	18.50004	19.80458	22.70327
H	20.40904	21.30410	22.74568
H	18.40026	24.00330	23.16033
H	17.96682	26.41978	22.69743

H	19.32072	27.12035	21.74442	H	27.72827	23.64128	13.06915
H	19.15552	27.54446	23.46846	H	27.94640	23.26705	10.63225
H	18.18202	20.18742	25.70157	H	29.51594	26.69442	10.17681
H	19.85608	21.79700	25.71414	H	31.55546	26.12187	13.30992
H	20.99066	20.61336	24.97914	H	24.95608	29.32808	12.79790
H	19.33929	18.34225	23.91451	H	25.35823	28.31324	11.50245
H	32.17233	27.23688	21.30243	H	30.65483	22.79833	10.74854
H	33.78433	26.31515	22.49132	H	31.47853	25.31212	10.00860
H	32.92897	26.74732	24.01213	H	31.38325	24.84079	11.73979
H	32.91121	24.29941	23.23017	H	29.21608	24.31763	8.81674
H	29.54283	23.51851	24.04337	H	17.52006	17.25369	20.22137
H	29.91849	27.04643	22.67205	H	18.25690	17.85740	22.02315
H	25.14425	28.04413	22.54573	H	18.58695	17.70927	17.75732
H	23.63671	27.15407	22.90060	H	23.22333	17.72731	21.29602
H	25.02565	22.90253	23.73827	H	27.36062	30.36707	16.12065
H	30.99703	25.50395	21.17242	H	18.80902	18.94074	19.95207
H	30.98943	22.64000	22.30009	H	20.64595	16.77715	21.14252
H	29.69842	23.39202	21.30258	H	20.80966	17.03298	18.60663
H	32.82890	23.64568	16.61780	H	20.54927	19.85249	21.36152
H	33.21919	22.99874	18.25229	H	20.63425	20.09105	18.93417
H	31.59241	21.56244	17.26123	H	22.64995	19.71117	22.54483
H	28.64647	21.57545	19.06141	H	21.51813	18.58782	23.37001
H	30.00218	24.89872	18.64033	H	20.98804	21.62913	17.62113
H	28.69964	26.97070	19.25414	H	24.67069	19.31156	17.46626
H	26.40410	27.76537	19.64565	H	25.97327	21.45017	17.01802
H	25.02684	26.69853	20.00125	H	22.07684	25.88792	17.06230
H	30.09342	24.02751	16.28511	H	26.79107	25.85925	16.63060
H	28.90942	21.23373	16.70697	H	22.87362	27.66652	17.16751
H	27.97128	22.77646	16.63759	H	27.79741	28.10841	16.42154
H	28.82591	22.24619	12.69614	H	23.86751	29.88936	16.83601

H	26.66622	30.16857	14.18713
H	24.63511	24.29015	20.26604
H	23.22425	24.79549	23.44140
O	28.34227	25.45570	14.53967
O	27.24382	25.16629	14.02070

A0

O	20.38079	27.14246	17.04612
C	19.05949	27.65079	17.07721
C	18.32917	27.45397	15.75542
O	19.00974	28.21299	14.71760
C	19.11020	27.48959	13.52368
N	20.54709	27.24673	13.19547
C	21.50187	27.26576	14.15439
C	22.82785	27.08650	13.81542
C	23.10132	26.90104	12.42762
N	24.42311	26.84749	11.99715
N	22.17522	26.86342	11.48100
C	20.84948	27.01436	11.80819
O	19.92228	26.97050	10.99807
C	18.20347	26.00373	15.22325
C	18.31505	26.19482	13.70244
O	16.95325	25.39702	15.48672
P	16.66206	24.56539	16.92661
O	15.40738	23.75586	16.71222
O	16.89391	25.52057	18.09732
O	18.01466	23.54693	16.94396
C	17.88195	22.18297	16.59039
C	18.13123	21.26089	17.78628
O	19.37448	21.61060	18.43356
C	19.10992	22.30294	19.66507

N	19.89016	23.52212	19.71103
C	19.55707	24.79210	19.27510
N	20.60199	25.60516	19.25865
C	21.67347	24.83842	19.68956
C	23.06975	25.12726	19.80332
O	23.63208	26.23036	19.62713
N	23.81744	23.97544	20.11094
C	23.30031	22.71118	20.31611
N	24.17125	21.70205	20.52603
N	21.98711	22.45471	20.29379
C	21.25023	23.52499	19.95263
C	17.10883	21.29766	18.93407
C	17.59517	22.48280	19.76893
O	17.32880	20.08479	19.67397
P	16.20836	19.39953	20.69789
O	16.96840	18.14591	21.21394
O	14.84939	19.32377	20.06722
O	16.14638	20.52393	21.94871
C	16.79422	20.33273	23.18983
C	18.29623	20.54064	23.18427
O	18.66428	21.89751	22.89869
C	20.03107	21.95860	23.26592
N	20.48288	23.34887	23.19874
C	19.63662	24.38275	22.86526
C	20.07815	25.66145	22.65474
C	19.18830	26.79181	22.23737
C	21.50956	25.92321	22.78335
O	22.01465	27.05002	22.61707
N	22.29834	24.82495	23.11228
C	21.87303	23.53135	23.32005

O	22.62750	22.59807	23.59765
C	18.98404	20.18951	24.53703
C	20.09371	21.27631	24.63855
O	19.42749	18.85807	24.61460
O	32.09794	27.81851	21.87262
C	32.88797	27.00433	22.69751
C	32.31662	25.60407	22.95081
O	31.21390	25.63030	23.88157
C	30.17447	24.78046	23.42817
N	29.03488	25.61495	23.01011
C	29.06336	26.93966	22.59318
N	27.85602	27.45819	22.42075
C	26.99739	26.42763	22.73944
C	25.57931	26.34617	22.77763
N	24.78113	27.39176	22.50550
N	25.03894	25.14633	23.12069
C	25.83889	24.09825	23.41650
N	27.17658	24.07035	23.44487
C	27.70077	25.26181	23.09871
C	31.76019	24.91576	21.69519
C	30.76817	23.92644	22.29922
O	32.78982	24.36548	20.90753
P	33.28036	25.24596	19.53982
O	34.75247	24.98756	19.36189
O	32.59906	26.61352	19.57330
O	32.41188	24.38221	18.35785
C	32.80086	23.03181	18.20586
C	31.58135	22.13810	18.06708
O	30.75372	22.23352	19.22820
C	29.38454	22.13931	18.88631
N	28.67902	23.37986	19.25964
C	29.26743	24.60840	19.12466
C	28.54284	25.75288	19.31336
C	27.13721	25.61010	19.59699
N	26.33000	26.67255	19.66977
N	26.59113	24.38981	19.79073
C	27.30940	23.25494	19.61757
O	26.83245	22.10693	19.73532
C	30.64826	22.43611	16.88153
C	29.31473	21.87390	17.37053
O	31.21138	21.76871	15.72702
P	30.57359	21.74889	14.25484
O	31.45768	21.05447	13.27735
O	30.15240	23.31856	13.90157
O	29.02900	21.18439	14.35968
C	28.06995	22.17769	13.94929
C	28.86203	23.27360	13.22651
O	28.21282	24.50129	13.32489
C	28.67661	25.38540	12.26934
N	29.28205	26.56172	12.83360
C	30.48420	26.70292	13.53926
N	30.58535	27.85484	14.15862
C	29.40362	28.51516	13.87838
C	28.82205	29.68318	14.47890
O	29.24392	30.40137	15.39652
N	27.51414	29.92091	13.95942
C	26.78774	29.07108	13.15182
N	25.51647	29.42186	12.81991
N	27.30418	27.94614	12.66520
C	28.56799	27.72272	13.06947

C	29.11352	23.09867	11.70481	C	27.20444	27.89836	16.48082
C	29.57520	24.52434	11.36603	C	25.03767	28.83225	17.03268
O	27.93638	22.71154	11.02089	C	26.41321	29.02670	16.78109
O	22.23642	18.93899	20.02267	H	20.54492	26.67608	17.93049
O	22.58624	19.07867	17.78905	H	18.43235	27.14330	17.84330
O	18.61998	16.97726	19.42507	H	19.06073	28.74772	17.30016
O	19.41769	17.79094	22.12182	H	17.29423	27.85328	15.86893
O	19.88615	18.11213	17.32572	H	18.73870	28.08221	12.66160
O	23.13644	17.68002	22.24280	H	21.15584	27.42072	15.19466
O	22.59843	23.77154	16.82973	H	23.61750	27.11239	14.57716
O	26.68862	23.77814	16.43306	H	24.52793	26.53759	11.02810
O	26.89620	30.30155	16.82673	H	25.14156	26.51022	12.64460
C	19.60959	17.95276	19.69993	H	19.03777	25.38846	15.62834
C	20.33092	17.77222	21.03360	H	18.76223	25.33882	13.16520
C	20.59882	17.95570	18.53556	H	17.28401	26.33428	13.32063
C	21.35521	18.92249	21.16318	H	18.64594	21.94583	15.81517
C	21.57084	19.10851	18.76578	H	16.87028	21.98325	16.17110
C	22.27744	18.80099	22.37348	H	18.24070	20.21729	17.42077
C	23.20645	20.25090	17.46198	H	19.49309	21.66817	20.48902
C	22.52357	21.46240	17.29001	H	18.53380	25.05937	18.94314
C	24.61024	20.18063	17.27140	H	25.18309	21.82930	20.31291
C	23.28439	22.59921	16.98335	H	23.75721	20.76748	20.48412
C	24.68813	22.57712	16.84062	H	16.05542	21.38089	18.59631
C	25.33449	21.32491	16.97212	H	17.24477	22.40636	20.81281
C	25.44956	23.81518	16.61497	H	17.25380	23.44079	19.33251
C	24.64577	25.05688	16.63148	H	16.62853	19.30147	23.57792
C	23.27251	24.92236	16.68579	H	16.33519	21.05254	23.90471
C	25.24709	26.41406	16.63072	H	18.74425	19.88409	22.40878
C	26.62312	26.62769	16.37202	H	20.66152	21.39971	22.53469
C	24.47600	27.55434	16.98475	H	18.58327	24.09134	22.75698

H	18.13238	26.46903	22.15748
H	19.51126	27.17260	21.24638
H	19.25888	27.64221	22.94804
H	18.25589	20.34822	25.36861
H	19.86164	22.02379	25.42391
H	21.08484	20.83720	24.84561
H	19.49495	18.47234	23.69655
H	32.25286	27.47197	20.93343
H	33.91175	26.85406	22.27223
H	33.01493	27.50730	23.68341
H	33.13108	24.96106	23.36801
H	29.78738	24.15936	24.26427
H	30.02461	27.45467	22.42656
H	25.23165	28.23922	22.16767
H	23.74083	27.28759	22.48178
H	25.29811	23.16429	23.65373
H	31.19734	25.67682	21.11296
H	31.31047	23.05899	22.72929
H	30.00473	23.55026	21.59680
H	33.42886	22.89687	17.29331
H	33.40463	22.68514	19.07157
H	31.94077	21.08658	17.93295
H	28.87931	21.34397	19.46710
H	30.34327	24.61395	18.87945
H	29.02287	26.73417	19.21585
H	26.71845	27.59755	19.50039
H	25.28716	26.53380	19.72444
H	30.60393	23.52539	16.67488
H	29.29090	20.78347	17.17367
H	28.41994	22.34748	16.92525
H	27.33913	21.70706	13.26415
H	27.55425	22.61782	14.83195
H	27.77142	25.75939	11.74756
H	31.21935	25.88835	13.57588
H	24.99455	30.02179	13.45955
H	24.98548	28.64388	12.37701
H	29.88071	22.32630	11.50157
H	29.44486	24.76033	10.29383
H	30.64046	24.65130	11.63517
H	27.21192	23.28913	11.33255
H	17.82397	17.27601	19.95542
H	18.46478	17.91093	21.78241
H	19.03042	17.67120	17.53822
H	23.45174	17.73175	21.31731
H	27.85555	30.32410	16.55960
H	19.13161	18.95921	19.71680
H	20.89193	16.80818	21.04334
H	21.19505	17.00685	18.53799
H	20.79966	19.88851	21.20541
H	21.01683	20.07346	18.75731
H	22.84215	19.75854	22.49136
H	21.65960	18.66719	23.28483
H	21.43565	21.56177	17.42453
H	25.10083	19.20801	17.41678
H	26.42721	21.29480	16.87567
H	22.56275	25.75945	16.63945
H	27.25009	25.76224	16.12747
H	23.42253	27.45169	17.28010
H	28.29081	28.00542	16.34529
H	24.42175	29.70154	17.30810

H	27.01635	30.65522	14.47684
H	24.87358	24.11074	20.08182
H	23.37319	24.96062	23.14561

H_2O_2

O	-0.81901	0.16643	0.01703
O	0.64808	0.15037	-0.01703
H	-0.97794	-0.53319	-0.64775
H	0.80701	0.84999	0.64775

$^{2}H_2O_2^{-\bullet}$

O	-1.29288	0.16928	0.02580
O	1.12197	0.14753	-0.02580
H	-0.66740	-0.37868	-0.50445
H	0.49645	0.69546	0.50444

OH^-

O	-0.80998	0.18124	0.03103
H	-0.96172	-0.53297	-0.64775

$OH^\bullet$

O	-0.81053	0.17863	0.02855
H	-0.96117	-0.53036	-0.64527

$^{1}O_2$

O	-6.15439	2.07364	0.00000
O	-4.94077	2.07364	0.00000

$^{2}O_2^{-\bullet}$

O	-6.22440	2.07364	0.00000
O	-4.87076	2.07364	0.00000

A1

O	20.92905	27.50220	17.44264
C	19.73468	27.96755	16.84900
C	19.37379	27.24069	15.56031
O	20.43281	27.36851	14.59774
C	20.26192	26.30918	13.66872
N	21.50446	26.12412	12.92415
C	22.67733	26.03272	13.60797
C	23.85444	25.72338	12.97527
C	23.75390	25.45664	11.55937
N	24.85986	25.05964	10.88500
N	22.61898	25.59548	10.87022
C	21.45056	25.94679	11.48675
O	20.37423	26.09978	10.90522
C	19.05798	25.71910	15.68698
C	19.84849	25.09734	14.52321
O	17.66922	25.46379	15.52659
P	16.70884	25.31123	16.90984
O	15.32480	24.96483	16.42178
O	17.06408	26.40391	17.91170
O	17.44628	23.94624	17.59928
C	17.34052	22.66982	17.01914
C	17.80952	21.63058	18.03922
O	19.13389	21.95864	18.51345
C	19.05572	22.56442	19.81443
N	19.81043	23.80197	19.85542
C	19.42383	25.10674	19.56866
N	20.44352	25.94883	19.61783
C	21.55085	25.17737	19.92751
C	22.94354	25.51254	20.01668
O	23.45473	26.64931	19.96816
N	23.74379	24.36006	20.13423
C	23.27911	23.06378	20.20222
N	24.20466	22.07559	20.22574

N	21.97511	22.75595	20.23176
C	21.18317	23.82815	20.05005
C	16.97533	21.54859	19.32787
C	17.56879	22.67967	20.16767
O	17.27767	20.29140	19.95617
P	16.12226	19.43683	20.80090
O	16.85330	18.10631	21.12580
O	14.78573	19.48819	20.11764
O	16.00087	20.32642	22.22124
C	16.76201	20.02137	23.37310
C	18.25536	20.25259	23.26051
O	18.58663	21.63596	23.09358
C	19.98487	21.67016	23.31457
N	20.42827	23.06282	23.34886
C	19.56382	24.11927	23.16184
C	19.99099	25.41584	23.06520
C	19.08248	26.57902	22.80976
C	21.42573	25.67470	23.17080
O	21.91486	26.81993	23.13227
N	22.23210	24.55410	23.33148
C	21.82318	23.24347	23.41264
O	22.59098	22.28974	23.54867
C	19.06416	19.77365	24.50152
C	20.19220	20.84387	24.59240
O	19.48643	18.43644	24.41490
O	32.05338	27.63019	22.65269
C	32.79035	26.70785	23.40861
C	32.21083	25.28693	23.43213
O	31.06443	25.17394	24.30009
C	30.04436	24.42101	23.66332
N	28.92740	25.32799	23.35262
C	28.97417	26.70116	23.15745
N	27.77377	27.25573	23.05853
C	26.90060	26.19653	23.19417
C	25.48216	26.12040	23.21221
N	24.68514	27.19867	23.10911
N	24.93521	24.88566	23.36608
C	25.72241	23.79648	23.50727
N	27.05816	23.75588	23.53760
C	27.59090	24.98108	23.37183
C	31.72827	24.79977	22.05943
C	30.68626	23.75202	22.43843
O	32.80898	24.36268	21.27505
P	33.30078	25.39294	20.00507
O	34.77395	25.15247	19.80744
O	32.65289	26.76268	20.24046
O	32.40480	24.70221	18.75896
C	32.71974	23.34128	18.48794
C	31.55771	22.63695	17.80275
O	30.40387	22.66953	18.65774
C	29.29966	23.26668	17.98495
N	28.56960	24.15995	18.89378
C	29.15233	25.33367	19.29447
C	28.41213	26.33158	19.86572
C	26.98995	26.11544	19.94889
N	26.14601	27.12056	20.21941
N	26.47211	24.88910	19.74548
C	27.22896	23.85860	19.27272
O	26.79069	22.70218	19.14283
C	31.13237	23.20326	16.42958

C	29.88193	24.01030	16.77716
O	30.86199	22.09256	15.56260
P	30.70169	22.38299	13.92480
O	30.53381	21.04107	13.23971
O	31.69963	23.45052	13.47647
O	29.21121	23.20140	13.90469
C	28.03007	22.49280	13.59029
C	27.59627	22.85652	12.17643
O	27.31508	24.30858	12.11847
C	28.40611	25.04627	11.59116
N	28.91932	26.02812	12.53989
C	30.13679	26.10259	13.23125
N	30.24142	27.19538	13.96068
C	29.04972	27.86635	13.77536
C	28.53732	29.06290	14.37214
O	29.05654	29.83778	15.20204
N	27.20449	29.31076	13.94051
C	26.41930	28.45733	13.19531
N	25.08397	28.77474	13.08907
N	26.90892	27.38938	12.58865
C	28.19883	27.14387	12.90983
C	28.69491	22.69898	11.08147
C	29.45521	24.02137	11.10632
O	28.03707	22.46133	9.81537
O	22.01845	18.98043	19.59110
O	22.19508	19.28940	17.35241
O	18.36212	17.06352	19.13767
O	19.37374	17.67695	21.81316
O	19.44590	18.38667	17.03848
O	23.09523	17.60731	21.64501
O	22.31966	24.06018	16.98803
O	26.33310	24.00998	16.13418
O	26.87711	30.43142	16.63288
C	19.37585	18.02168	19.40146
C	20.20060	17.74851	20.65760
C	20.26263	18.12189	18.16166
C	21.22934	18.89468	20.78614
C	21.26576	19.24807	18.40070
C	22.23854	18.70408	21.91630
C	22.85051	20.47750	17.12830
C	22.19935	21.71519	17.14982
C	24.23575	20.39168	16.84197
C	22.97379	22.86212	16.92488
C	24.35662	22.81748	16.66275
C	24.97331	21.54660	16.61103
C	25.13931	24.05564	16.48200
C	24.38626	25.30574	16.74441
C	23.02668	25.20927	16.94102
C	25.03351	26.63980	16.76227
C	26.44285	26.76563	16.80733
C	24.27415	27.83933	16.78158
C	27.07099	28.01352	16.84285
C	24.90048	29.09154	16.77189
C	26.30877	29.19561	16.77315
H	20.71446	27.09780	18.33912
H	18.85393	27.83098	17.51685
H	19.83605	29.05590	16.61427
H	18.43915	27.69914	15.15188
H	19.49425	26.54945	12.90054
H	22.61382	26.23561	14.68640

H	24.79846	25.61534	13.52593	H	32.85557	27.07282	24.46011
H	24.69892	24.75818	9.92476	H	33.00707	24.58714	23.78934
H	25.71287	24.76650	11.39162	H	29.61667	23.67482	24.36710
H	19.42140	25.32395	16.65917	H	29.94311	27.22369	23.08374
H	20.75241	24.57624	14.89466	H	25.13494	28.07631	22.85947
H	19.22270	24.37499	13.96722	H	23.64725	27.09428	23.04165
H	17.98712	22.58999	16.11196	H	25.17062	22.84398	23.60150
H	16.29228	22.45692	16.70178	H	31.20856	25.64674	21.56197
H	17.87475	20.63138	17.55811	H	31.18750	22.81270	22.75282
H	19.58108	21.88492	20.51688	H	29.96827	23.51328	21.63214
H	18.40578	25.39220	19.25203	H	33.62846	23.26740	17.84645
H	25.17397	22.26992	19.89139	H	32.93432	22.80451	19.43849
H	23.82229	21.14062	20.07351	H	31.85912	21.57710	17.63515
H	15.88342	21.62925	19.14895	H	28.56081	22.49209	17.69536
H	17.38710	22.51658	21.24374	H	30.23786	25.42063	19.11857
H	17.15592	23.64586	19.82947	H	28.87940	27.27613	20.16760
H	16.64075	18.95192	23.66395	H	26.53238	28.05086	20.35621
H	16.36101	20.65573	24.19583	H	25.10641	26.97184	20.13799
H	18.64129	19.68983	22.38484	H	31.91968	23.83897	15.97273
H	20.52877	21.18989	22.46659	H	29.17164	24.08230	15.93531
H	18.50720	23.83354	23.07540	H	30.19606	25.03168	17.06603
H	18.02342	26.26138	22.74879	H	28.19474	21.39516	13.63189
H	19.35832	27.06248	21.84952	H	27.22609	22.78974	14.29760
H	19.18859	27.35108	23.60113	H	27.99063	25.65903	10.75645
H	18.42195	19.85669	25.41108	H	30.86761	25.26997	13.21266
H	20.06018	21.50354	25.47380	H	24.67814	29.16275	13.94917
H	21.19544	20.38709	24.63888	H	24.53567	27.96923	12.75732
H	19.54149	18.16728	23.45516	H	29.33980	21.82322	11.29647
H	32.25644	27.40125	21.68079	H	29.85344	24.27613	10.10283
H	33.83854	26.60776	23.02957	H	30.31685	23.94037	11.80146

H	27.68917	23.32792	9.52739
H	17.61185	17.32529	19.74581
H	18.40111	17.81803	21.55749
H	18.61275	17.92047	17.28033
H	23.35286	17.74014	20.70924
H	27.82015	30.30381	16.29971
H	18.90357	19.02145	19.53432
H	20.76509	16.79225	20.55416
H	20.84626	17.17459	18.02894
H	20.67340	19.85145	20.93544
H	20.71996	20.21260	18.51350
H	22.80006	19.66009	22.05802
H	21.69138	18.49538	22.85864
H	21.12881	21.82834	17.37463
H	24.70345	19.39691	16.83878
H	26.05502	21.51505	16.41978
H	22.34380	26.06560	17.07823
H	27.05427	25.85690	16.80027
H	23.17314	27.81825	16.81929
H	28.16878	28.06984	16.88050
H	24.30000	30.01503	16.76536
H	26.75441	30.07507	14.45976
H	24.79728	24.53538	20.04642
H	23.31408	24.69764	23.35391
O	26.44518	22.17009	11.84793
H	26.53228	22.01980	10.87631

A1R

O	20.87978	27.40951	17.44972
C	19.63233	27.86725	16.96790
C	19.20704	27.20014	15.66806
O	20.16375	27.50071	14.63764
C	20.13927	26.43960	13.69911
N	21.39462	26.47538	12.94765
C	22.56890	26.52392	13.63280
C	23.77626	26.56231	12.98205
C	23.70852	26.57886	11.54667
N	24.86420	26.72622	10.81929
N	22.56888	26.53841	10.86294
C	21.35787	26.47561	11.50375
O	20.27227	26.42195	10.92455
C	19.04550	25.64879	15.70621
C	19.91969	25.16940	14.53631
O	17.69525	25.25969	15.48899
P	16.70004	25.07611	16.84521
O	15.34870	24.65762	16.32255
O	16.97728	26.19619	17.84111
O	17.47827	23.75368	17.57036
C	17.39102	22.45886	17.02819
C	17.83781	21.45349	18.09010
O	19.14751	21.80517	18.58837
C	19.03255	22.44856	19.86781
N	19.77745	23.69260	19.88782
C	19.38280	24.98777	19.57082
N	20.39638	25.83791	19.60730
C	21.50723	25.08103	19.93693
C	22.89748	25.42851	20.00761
O	23.39714	26.56836	19.92183
N	23.70699	24.28611	20.14897
C	23.25140	22.98816	20.25036
N	24.18433	22.00807	20.29349

N	21.94978	22.67097	20.29116
C	21.14917	23.73258	20.08697
C	16.97439	21.40061	19.36090
C	17.53672	22.56183	20.18144
O	17.27927	20.16556	20.02960
P	16.13272	19.32892	20.90297
O	16.87867	18.01806	21.27096
O	14.79562	19.34392	20.21917
O	16.00351	20.26429	22.29328
C	16.75853	19.99695	23.45841
C	18.25065	20.23877	23.35086
O	18.57133	21.62066	23.15148
C	19.96697	21.67365	23.38532
N	20.39730	23.07085	23.39025
C	19.52462	24.11509	23.17557
C	19.94127	25.41273	23.05012
C	19.02369	26.56182	22.76527
C	21.37382	25.68593	23.15088
O	21.85474	26.83318	23.08158
N	22.18854	24.57647	23.34304
C	21.79025	23.26515	23.45619
O	22.56538	22.32165	23.61992
C	19.05305	19.79818	24.61020
C	20.17012	20.88079	24.68469
O	19.48889	18.46351	24.56089
O	32.00504	27.65138	22.52868
C	32.73959	26.76373	23.32719
C	32.16231	25.34398	23.40743
O	31.01242	25.26632	24.27471
C	29.99528	24.48688	23.66625
N	28.87751	25.37889	23.31657
C	28.92014	26.74439	23.07266
N	27.71807	27.29182	22.95560
C	26.84778	26.23575	23.13001
C	25.42952	26.15596	23.15654
N	24.62756	27.22672	23.01864
N	24.88748	24.92584	23.35789
C	25.67826	23.84476	23.53559
N	27.01412	23.80948	23.56250
C	27.54213	25.02944	23.35014
C	31.68639	24.79872	22.05432
C	30.64132	23.76883	22.47182
O	32.77064	24.32567	21.29587
P	33.27137	25.29892	19.98532
O	34.74175	25.03553	19.79691
O	32.63742	26.68333	20.16507
O	32.36687	24.56847	18.76894
C	32.66438	23.19480	18.54952
C	31.49605	22.48641	17.87857
O	30.33703	22.56611	18.72338
C	29.25631	23.17791	18.02471
N	28.52706	24.09758	18.90471
C	29.10262	25.28696	19.26860
C	28.35409	26.30011	19.80028
C	26.93292	26.07875	19.88647
N	26.08223	27.08693	20.12098
N	26.42280	24.84285	19.72522
C	27.18668	23.80236	19.28847
O	26.75532	22.63983	19.19326
C	31.08962	23.02410	16.48925

C	29.87329	23.88756	16.81487
O	30.76463	21.90092	15.65522
P	30.64178	22.15775	14.00868
O	30.40429	20.81429	13.35208
O	31.68450	23.17804	13.55643
O	29.18670	23.04656	13.93855
C	27.98696	22.35869	13.70676
C	27.62340	22.27316	12.24896
O	27.29574	24.84697	11.49675
C	28.67186	25.13317	11.29788
N	29.10867	26.00437	12.38788
C	30.16450	25.88592	13.30354
N	30.26303	26.92806	14.10575
C	29.23496	27.76700	13.73274
C	28.76227	28.99391	14.30324
O	29.20429	29.65847	15.26091
N	27.56877	29.43245	13.65797
C	26.92308	28.79649	12.62365
N	25.76342	29.36181	12.13728
N	27.39483	27.69717	12.06098
C	28.50332	27.20992	12.66321
C	28.63411	22.58002	11.11259
C	29.50817	23.83820	11.15141
O	27.85404	22.59967	9.91399
O	22.04127	18.92155	19.73367
O	22.21571	19.18371	17.48770
O	18.41244	16.94464	19.31601
O	19.40456	17.63839	21.97841
O	19.48408	18.23205	17.18926
O	23.12768	17.60464	21.82124
O	22.31072	23.95168	17.02198
O	26.31603	23.88492	16.12817
O	26.87362	30.31329	16.37910
C	19.41220	17.92267	19.56004
C	20.23584	17.69074	20.82462
C	20.30124	18.00698	18.32096
C	21.25035	18.85223	20.92894
C	21.28657	19.15355	18.53636
C	22.25860	18.69809	22.06542
C	22.85759	20.37237	17.23225
C	22.20001	21.60734	17.23581
C	24.23962	20.28642	16.92994
C	22.96616	22.75337	16.97655
C	24.34553	22.70757	16.69739
C	24.96814	21.43886	16.66497
C	25.12280	23.94164	16.47950
C	24.37011	25.19804	16.71325
C	23.01374	25.10166	16.93230
C	25.01769	26.53198	16.67946
C	26.42754	26.65427	16.68326
C	24.26463	27.73634	16.67636
C	27.06037	27.89951	16.64628
C	24.89338	28.98373	16.59495
C	26.30026	29.08176	16.54887
H	20.71636	26.98639	18.34786
H	18.80742	27.66492	17.68686
H	19.67934	28.96968	16.78704
H	18.20485	27.59889	15.37924
H	19.34114	26.56916	12.93547
H	22.48104	26.55381	14.72760

H	24.72186	26.57146	13.53983	H	32.79799	27.17269	24.36279
H	24.74914	26.56574	9.81715	H	32.95768	24.66084	23.79720
H	25.78320	26.49220	11.23249	H	29.56645	23.76931	24.39855
H	19.42233	25.23725	16.66644	H	29.88759	27.26624	22.97839
H	20.89135	24.78794	14.90599	H	25.07165	28.09641	22.73363
H	19.41408	24.36371	13.97276	H	23.58993	27.11583	22.96314
H	18.06052	22.35396	16.14047	H	25.12957	22.89476	23.66636
H	16.35149	22.23418	16.69165	H	31.17059	25.62422	21.51821
H	17.92305	20.44149	17.63992	H	31.14061	22.84274	22.82610
H	19.54457	21.79455	20.60316	H	29.92676	23.49807	21.67257
H	18.36326	25.25466	19.24386	H	33.57783	23.08483	17.91995
H	25.15165	22.20502	19.95457	H	32.86163	22.68866	19.52058
H	23.80917	21.06688	20.16210	H	31.77983	21.41687	17.74542
H	15.88569	21.46317	19.15664	H	28.50814	22.41291	17.73342
H	17.32986	22.42916	21.25730	H	30.18852	25.37389	19.09684
H	17.12354	23.51366	19.80482	H	28.81413	27.25733	20.07153
H	16.64469	18.93457	23.77668	H	26.46113	28.02434	20.22600
H	16.34579	20.64930	24.26106	H	25.04391	26.92400	20.05397
H	18.64841	19.65734	22.49286	H	31.90078	23.61085	16.00996
H	20.52422	21.17936	22.55422	H	29.17209	23.97812	15.96767
H	18.47082	23.81802	23.09205	H	30.22824	24.89841	17.09188
H	17.96734	26.23416	22.71047	H	28.02750	21.30537	14.07364
H	19.29736	27.02433	21.79416	H	27.15970	22.86746	14.25095
H	19.12203	27.35359	23.53796	H	28.77506	25.74971	10.37097
H	18.40185	19.89758	25.51173	H	30.80489	24.98310	13.35298
H	20.02425	21.56047	25.54855	H	25.15183	29.78147	12.84277
H	21.17705	20.43466	24.75104	H	25.26830	28.69214	11.52433
H	19.55482	18.16997	23.60906	H	29.32781	21.70107	11.15873
H	32.21773	27.38269	21.56912	H	30.03614	23.86905	10.17584
H	33.79004	26.65028	22.95849	H	30.29034	23.73133	11.92741

H	27.08191	24.20368	10.77605
H	17.65594	17.21128	19.91413
H	18.43152	17.76019	21.71530
H	18.65717	17.75955	17.44036
H	23.38631	17.71857	20.88328
H	27.84773	30.17200	16.16957
H	18.92568	18.91829	19.66877
H	20.81184	16.73890	20.74754
H	20.89983	17.06605	18.21185
H	20.68274	19.80524	21.05616
H	20.72771	20.11256	18.62792
H	22.80960	19.66285	22.18723
H	21.71077	18.50480	23.01071
H	21.13127	21.71898	17.46974
H	24.71123	19.29358	16.94165
H	26.04709	21.40949	16.45861
H	22.33278	25.95925	17.06615
H	27.03773	25.74524	16.70417
H	23.16566	27.72362	16.75549
H	28.15840	27.94910	16.66843
H	24.29400	29.90700	16.56776
H	27.10099	30.19870	14.16038
H	24.75849	24.46738	20.04583
H	23.26964	24.72919	23.36013
O	26.52955	21.83078	11.88562
H	27.03942	22.10437	10.22211

A1RR

O	20.95451	27.64839	17.41315
C	19.82925	28.06297	16.67117
C	19.56049	27.23122	15.42294
O	20.68222	27.23577	14.52248
C	20.42543	26.16001	13.62452
N	21.61096	25.87294	12.82783
C	22.82288	25.74465	13.43547
C	23.93914	25.35939	12.74113
C	23.73892	25.05858	11.34450
N	24.77498	24.58856	10.60842
N	22.56448	25.21456	10.72912
C	21.45487	25.63590	11.40585
O	20.34838	25.80809	10.88854
C	19.17787	25.73652	15.64659
C	19.95502	25.01121	14.53265
O	17.78060	25.53966	15.47708
P	16.77812	25.48077	16.83585
O	15.38880	25.21404	16.31478
O	17.17153	26.56623	17.83112
O	17.40883	24.08340	17.56923
C	17.36291	22.83195	16.93055
C	17.88963	21.77209	17.89832
O	19.21071	22.12900	18.36175
C	19.13684	22.66447	19.69309
N	19.86660	23.91320	19.78468
C	19.46582	25.22149	19.53333
N	20.47366	26.07494	19.61681
C	21.58942	25.30827	19.91131
C	22.97985	25.65096	20.02901
O	23.48904	26.78884	20.04088
N	23.78810	24.49791	20.10128
C	23.33581	23.19625	20.12228
N	24.26953	22.21433	20.11363

N	22.03572	22.87854	20.13855
C	21.23652	23.95108	19.98839
C	17.08039	21.61175	19.19540
C	17.65388	22.72481	20.07250
O	17.42809	20.34010	19.76810
P	16.30000	19.40350	20.56041
O	17.07636	18.08433	20.81944
O	14.96466	19.44362	19.87395
O	16.14172	20.21655	22.02255
C	16.91390	19.88622	23.15974
C	18.40044	20.15945	23.05583
O	18.70349	21.55714	22.95816
C	20.10266	21.60048	23.17165
N	20.53527	22.99366	23.26351
C	19.66557	24.05269	23.12031
C	20.08660	25.35460	23.07804
C	19.17315	26.52310	22.86815
C	21.51993	25.61602	23.19779
O	22.00522	26.76357	23.21420
N	22.33135	24.49304	23.30630
C	21.92913	23.17832	23.32893
O	22.70361	22.22437	23.41844
C	19.22403	19.63422	24.26858
C	20.32834	20.72250	24.41095
O	19.67470	18.31306	24.11084
O	32.15968	27.59038	22.65173
C	32.90432	26.63181	23.35347
C	32.31734	25.21428	23.32001
O	31.18302	25.07001	24.19859
C	30.14812	24.35221	23.54615
N	29.02804	25.27585	23.30348
C	29.07118	26.65853	23.19239
N	27.86920	27.21656	23.14721
C	26.99802	26.14995	23.23198
C	25.57954	26.06896	23.26193
N	24.77723	27.14807	23.23202
N	25.03728	24.82572	23.34626
C	25.82732	23.73183	23.41059
N	27.16374	23.69261	23.42038
C	27.69187	24.92683	23.32342
C	31.81169	24.79414	21.93367
C	30.76336	23.74065	22.27802
O	32.87813	24.37836	21.11644
P	33.37526	25.45906	19.89243
O	34.84038	25.20061	19.66098
O	32.74856	26.82333	20.19313
O	32.45892	24.83364	18.62021
C	32.72046	23.46869	18.32158
C	31.52646	22.83914	17.61961
O	30.38364	22.87492	18.48400
C	29.30514	23.56684	17.85906
N	28.60660	24.41282	18.82779
C	29.18602	25.57135	19.27758
C	28.44616	26.52852	19.91579
C	27.02711	26.28991	20.01621
N	26.17330	27.27037	20.33893
N	26.52116	25.06796	19.76570
C	27.28072	24.07125	19.23295
O	26.86246	22.91375	19.06481
C	31.12127	23.51549	16.29386

C	29.92219	24.36572	16.70302	O	22.44464	24.41532	16.96845
O	30.79445	22.48764	15.34444	O	26.47954	24.46901	16.16617
P	30.69812	22.89997	13.73174	O	26.83501	30.81976	15.98791
O	30.68430	21.61852	12.93094	C	19.59489	18.18113	19.07759
O	31.62826	24.07655	13.42661	C	20.43307	17.86669	20.31440
O	29.14663	23.59356	13.68227	C	20.47397	18.37465	17.84354
C	28.03137	22.76171	13.96171	C	21.42659	19.03510	20.50267
C	27.20498	22.41779	12.74350	C	21.44436	19.51700	18.13730
O	27.35740	25.08879	11.75379	C	22.44498	18.81104	21.61838
C	28.51574	24.71099	11.55022	C	23.01221	20.83642	16.93199
N	31.47685	26.55024	14.39978	C	22.35217	22.06627	17.01835
C	32.24589	27.18405	15.35965	C	24.39753	20.77652	16.64099
N	31.76567	28.34893	15.76036	C	23.11543	23.23130	16.84722
C	30.60115	28.49077	15.03366	C	24.50090	23.20997	16.57992
C	29.56030	29.46980	15.10101	C	25.12497	21.94637	16.46852
O	29.44626	30.47168	15.84462	C	25.26332	24.46365	16.44388
N	28.51146	29.16315	14.19746	C	24.46942	25.68973	16.64496
C	28.37437	27.99635	13.46972	C	23.12104	25.58206	16.89086
N	27.20330	27.81322	12.78458	C	25.07709	27.03212	16.52084
N	29.34010	27.09656	13.39548	C	26.40877	27.26996	16.92970
C	30.40344	27.36871	14.18513	C	24.34538	28.12589	15.99894
C	27.84382	22.25903	11.34869	C	26.99184	28.53573	16.82683
C	28.82516	23.37376	10.93537	C	24.93561	29.38698	15.85388
O	26.80312	22.10547	10.41324	C	26.27362	29.60554	16.24819
O	22.20879	19.21248	19.31249	H	20.67450	27.25002	18.28852
O	22.36960	19.63176	17.09092	H	18.88858	27.99904	17.26472
O	18.61193	17.20555	18.76503	H	19.97603	29.12367	16.34993
O	19.61503	17.70858	21.46736	H	18.67414	27.67614	14.90441
O	19.64709	18.67139	16.73648	H	19.63930	26.42895	12.88472
O	23.33339	17.75786	21.28237	H	22.84494	25.97699	14.50852

H	24.90905	25.24319	13.24152
H	24.58656	24.35715	9.63528
H	25.72437	24.49673	10.99017
H	19.51088	25.39291	16.64892
H	20.83392	24.47636	14.94203
H	19.30511	24.28932	14.00435
H	18.00357	22.82650	16.01638
H	16.32495	22.57663	16.60846
H	17.98009	20.79587	17.37622
H	19.68747	21.96389	20.35486
H	18.44911	25.50284	19.20833
H	25.23748	22.43061	19.79476
H	23.89545	21.28364	19.91802
H	15.98406	21.66658	19.03512
H	17.49363	22.51265	21.14357
H	17.21006	23.69156	19.77773
H	16.82280	18.80263	23.40642
H	16.49779	20.47475	24.00866
H	18.79663	19.65119	22.15217
H	20.64544	21.16350	22.30031
H	18.61032	23.76532	23.02174
H	18.11477	26.20426	22.80205
H	19.44175	27.04038	21.92345
H	19.28167	27.26728	23.68536
H	18.58409	19.65403	25.18311
H	20.18084	21.33921	25.32069
H	21.34114	20.28590	24.43842
H	19.75480	18.10372	23.13819
H	32.35360	27.41141	21.66983
H	33.94653	26.54635	22.95541
H	32.98543	26.94772	24.41944
H	33.11368	24.49382	23.63301
H	29.73205	23.57270	24.22027
H	30.03861	27.18472	23.13114
H	25.22178	28.04132	23.03346
H	23.73902	27.04372	23.15902
H	25.27825	22.77420	23.45427
H	31.29749	25.66874	21.48073
H	31.25930	22.78163	22.53636
H	30.02940	23.54951	21.47339
H	33.62591	23.36978	17.67775
H	32.91005	22.90026	19.25854
H	31.78263	21.77953	17.38759
H	28.53738	22.84437	17.51481
H	30.26597	25.68669	19.08649
H	28.90881	27.46142	20.25810
H	26.54820	28.19974	20.51077
H	25.13352	27.11247	20.24916
H	31.94786	24.13123	15.88541
H	29.20890	24.55669	15.88215
H	30.30162	25.33840	17.07004
H	28.35547	21.79312	14.41178
H	27.37160	23.27033	14.69987
H	29.36185	25.39944	11.79523
H	33.15402	26.71379	15.76222
H	26.35547	28.17542	13.23084
H	27.11707	26.84902	12.41110
H	28.46506	21.33051	11.45323
H	28.74361	23.48122	9.82932
H	29.86639	23.08068	11.16981

H	26.00707	21.99812	10.99983
H	17.85673	17.40663	19.38928
H	18.63626	17.82710	21.22322
H	18.83041	18.16507	16.95178
H	23.57856	17.94877	20.35331
H	27.84451	30.76846	16.09697
H	19.09081	19.15601	19.26529
H	21.02573	16.93501	20.15747
H	21.08680	17.45426	17.66146
H	20.84222	19.96446	20.70470
H	20.87255	20.45966	18.29691
H	22.97775	19.77316	21.81620
H	21.90694	18.53087	22.54738
H	21.27934	22.15528	17.24329
H	24.87196	19.78674	16.58421
H	26.20548	21.92521	16.27709
H	22.43307	26.43652	17.04054
H	27.00032	26.43831	17.32583
H	23.29817	27.99224	15.68621
H	28.02483	28.69345	17.17142
H	24.37121	30.22523	15.41758
H	27.71192	29.80426	14.26734
H	24.83797	24.68054	20.03151
H	23.41121	24.63806	23.33275
O	26.00792	22.15433	12.84221
H	31.57738	25.55787	13.99534

A2

O	20.32725	26.71953	16.79568
C	18.99317	27.11657	16.51446
C	18.55419	26.77429	15.09959
O	19.33612	27.54868	14.16236
C	19.52401	26.85839	12.95020
N	20.97101	26.87393	12.62160
C	21.88628	26.65089	13.60607
C	23.22583	26.60882	13.31619
C	23.60678	26.89279	11.96381
N	24.92856	26.94606	11.64543
N	22.71175	27.14854	11.00540
C	21.37821	27.08177	11.27100
O	20.50231	27.16504	10.38364
C	18.67739	25.29761	14.64820
C	18.94655	25.44323	13.13749
O	17.49887	24.53667	14.80891
P	17.08778	23.78011	16.26668
O	16.00225	22.78799	15.93217
O	16.99305	24.83553	17.36656
O	18.53544	22.96136	16.59357
C	18.64128	21.58521	16.27133
C	18.76219	20.70365	17.51609
O	19.81514	21.18810	18.37918
C	19.26149	21.89080	19.50167
N	19.85055	23.21743	19.58303
C	19.36627	24.42891	19.12206
N	20.28396	25.38431	19.16198
C	21.42906	24.77508	19.65165
C	22.77736	25.24860	19.76199
O	23.19493	26.41128	19.57104
N	23.66702	24.20155	20.06333
C	23.31975	22.88309	20.27855
N	24.32577	22.00895	20.51310

N	22.05402	22.45109	20.24921
C	21.18282	23.41337	19.89754
C	17.56055	20.61083	18.46831
C	17.73899	21.85139	19.34802
O	17.80329	19.43487	19.25933
P	16.59404	18.59963	20.04676
O	17.36169	17.37665	20.62075
O	15.36744	18.46622	19.19450
O	16.23860	19.63703	21.32257
C	16.74204	19.44852	22.62965
C	18.21634	19.74799	22.81267
O	18.53023	21.13382	22.60514
C	19.84806	21.24620	23.11093
N	20.24897	22.65104	23.11243
C	19.37153	23.67146	22.82331
C	19.77090	24.97511	22.69851
C	18.85075	26.09848	22.32987
C	21.18971	25.27710	22.87679
O	21.65795	26.42859	22.78320
N	22.00952	24.19222	23.16698
C	21.62687	22.87576	23.29276
O	22.40244	21.95491	23.55208
C	18.76582	19.39818	24.22808
C	19.80523	20.53313	24.46827
O	19.25778	18.08712	24.33502
O	31.69484	27.10761	21.35766
C	32.54243	26.31749	22.16254
C	31.96416	24.94700	22.53624
O	30.95287	25.04180	23.56454
C	29.85365	24.21041	23.24802
N	28.69776	25.07364	22.93653
C	28.71315	26.42127	22.60920
N	27.50369	26.96047	22.55075
C	26.65397	25.91512	22.84960
C	25.23839	25.82310	22.94024
N	24.41823	26.87141	22.74402
N	24.71798	24.60666	23.25524
C	25.52947	23.54636	23.46320
N	26.86516	23.51855	23.41026
C	27.37053	24.72522	23.09301
C	31.27654	24.22198	21.36913
C	30.29295	23.30623	22.08714
O	32.20256	23.57147	20.53467
P	32.91104	24.49688	19.30225
O	34.37764	24.68281	19.59853
O	31.98833	25.71134	19.04600
O	32.69536	23.45153	18.01602
C	32.33591	22.08760	18.16586
C	30.88030	21.82768	17.78686
O	30.00037	22.48721	18.72174
C	29.07434	23.25589	17.95334
N	28.40354	24.27601	18.73717
C	28.92240	25.53213	18.91705
C	28.12849	26.55206	19.37552
C	26.73560	26.26079	19.59824
N	25.82292	27.22227	19.80760
N	26.29659	24.98948	19.58634
C	27.09617	23.95781	19.21446
O	26.72786	22.77064	19.23363
C	30.46684	22.34369	16.38208

C	29.89888	23.71826	16.75470
O	29.40559	21.50742	15.91761
P	29.11953	21.13350	14.30573
O	27.85279	20.28852	14.38128
O	30.38849	20.80883	13.56910
O	28.63852	22.68517	13.69558
C	27.62354	23.34553	14.38312
C	26.25932	23.33281	13.68638
O	26.27258	24.20397	12.56903
C	25.69121	23.52096	11.43647
N	26.07672	24.20081	10.22993
C	27.30647	24.74730	9.86014
N	27.20913	25.60270	8.86364
C	25.85765	25.64961	8.56105
C	25.10763	26.48718	7.64462
O	25.48119	27.31413	6.81862
N	23.70159	26.24578	7.82617
C	23.10111	25.36306	8.69466
N	21.72822	25.30283	8.67985
N	23.80821	24.56159	9.47993
C	25.13573	24.77544	9.39390
C	25.71916	21.96048	13.15427
C	26.08802	22.07526	11.66993
O	24.32642	21.80516	13.35392
O	22.69620	18.43779	20.14453
O	23.29404	18.74845	17.98621
O	19.28019	16.33559	19.03527
O	19.68320	17.12049	21.83548
O	20.74898	17.63305	17.15189
O	23.35143	17.16397	22.45846
O	22.25579	23.25845	16.94041
O	25.37670	24.04697	14.62252
O	26.18644	29.95474	17.05264
C	20.17324	17.34598	19.46251
C	20.72813	17.16000	20.87495
C	21.30296	17.43930	18.43672
C	21.67658	18.34757	21.16100
C	22.16706	18.62841	18.84388
C	22.43502	18.24368	22.48061
C	23.57126	19.93466	17.40162
C	22.69714	21.02618	17.40471
C	24.83957	20.04626	16.75775
C	23.13648	22.21968	16.84710
C	24.39040	22.37603	16.18115
C	25.23420	21.22123	16.14389
C	24.63508	23.68233	15.65532
C	23.94749	24.79952	16.27339
C	22.69453	24.50353	16.77727
C	24.51340	26.16388	16.35814
C	25.91163	26.35761	16.28050
C	23.70528	27.29056	16.65365
C	26.48506	27.61399	16.48524
C	24.27403	28.54812	16.85808
C	25.66951	28.71830	16.78993
H	20.32522	26.38337	17.75018
H	18.26618	26.60417	17.18237
H	18.87885	28.22164	16.64005
H	17.47610	27.05111	15.00526
H	19.05225	27.38560	12.09518
H	21.47619	26.54583	14.62481

H	23.97149	26.44240	14.09879
H	25.17226	27.04083	10.65765
H	25.60134	26.46995	12.24985
H	19.52965	24.81815	15.18122
H	19.60734	24.65819	12.72328
H	17.96431	25.36160	12.63157
H	19.56547	21.43802	15.66734
H	17.76330	21.25433	15.67282
H	19.05727	19.68156	17.19460
H	19.58624	21.35306	20.41244
H	18.35522	24.55157	18.68609
H	25.28021	22.23997	20.16549
H	24.04130	21.02865	20.54240
H	16.57790	20.55786	17.95652
H	17.23224	21.72542	20.32117
H	17.34316	22.74699	18.83351
H	16.59885	18.39745	22.97096
H	16.15222	20.11544	23.29807
H	18.79618	19.14545	22.08212
H	20.57210	20.72765	22.43954
H	18.33297	23.34738	22.67005
H	17.80442	25.74958	22.23284
H	19.16334	26.53944	21.36046
H	18.89466	26.91391	23.08213
H	17.94451	19.50600	24.97614
H	19.45920	21.24814	25.24180
H	20.79072	20.13436	24.76583
H	19.49063	17.73838	23.42977
H	31.78596	26.72457	20.42952
H	33.51710	26.11882	21.65519
H	32.75508	26.87249	23.10412
H	32.79830	24.30268	22.90674
H	29.54344	23.62123	24.13813
H	29.66487	26.92681	22.38398
H	24.84302	27.71556	22.36670
H	23.38080	26.74309	22.72405
H	25.00039	22.60365	23.68917
H	30.70246	24.97782	20.79258
H	30.82713	22.42153	22.49184
H	29.45683	22.95382	21.45576
H	32.98183	21.48261	17.48897
H	32.49693	21.73882	19.20673
H	30.69693	20.72902	17.81242
H	28.25312	22.58937	17.61582
H	30.00427	25.65622	18.70247
H	28.54389	27.55345	19.54214
H	26.11865	28.19465	19.79347
H	24.80204	26.96362	19.77497
H	31.30596	22.36273	15.65601
H	29.31702	24.19045	15.94727
H	30.72235	24.38457	17.07936
H	27.47884	22.94354	15.41113
H	27.88339	24.42561	14.48255
H	24.57922	23.62656	11.44183
H	28.22454	24.47603	10.39853
H	21.26230	26.20374	8.93928
H	21.42171	24.60654	9.36674
H	26.25620	21.11699	13.63942
H	25.53059	21.34834	11.05055
H	27.18078	21.94330	11.54262

H	23.85157	22.51132	12.87506
H	18.40784	16.57767	19.46531
H	18.77677	17.19677	21.37650
H	19.90000	17.13699	17.21574
H	23.82716	17.26844	21.60975
H	27.15566	29.89724	16.95631
H	19.64508	18.32823	19.44709
H	21.32404	16.21934	20.93786
H	21.93893	16.51869	18.47628
H	21.06890	19.28409	21.14930
H	21.54217	19.54561	18.82549
H	22.93132	19.22447	22.68352
H	21.70556	18.06621	23.29718
H	21.68800	21.02917	17.84271
H	25.51766	19.18183	16.76220
H	26.23055	21.22765	15.67333
H	21.92643	25.24700	17.02788
H	26.58059	25.50902	16.09252
H	22.61081	27.20321	16.73909
H	27.58134	27.72362	16.45464
H	23.63993	29.41317	17.09860
H	23.10160	26.82101	7.22969
H	24.69738	24.47570	19.98943
H	23.08040	24.36905	23.24319

A2-OHS

O	20.29244	26.82395	16.62677
C	18.98520	27.34916	16.49142
C	18.37614	27.02964	15.13285
O	19.10725	27.72328	14.09190
C	19.27986	26.92511	12.94644
N	20.73376	26.78451	12.66739
C	21.62315	26.67941	13.69598
C	22.96667	26.57252	13.44221
C	23.38363	26.65200	12.07562
N	24.71140	26.65949	11.78528
N	22.50779	26.76254	11.06779
C	21.17227	26.75756	11.31453
O	20.30791	26.70644	10.40668
C	18.34131	25.53568	14.72929
C	18.55880	25.59385	13.20705
O	17.09825	24.90908	14.98330
P	16.76604	24.19262	16.48160
O	15.54811	23.32618	16.26724
O	16.89975	25.24138	17.58499
O	18.14767	23.23734	16.65247
C	18.10232	21.86225	16.31384
C	18.29687	20.97972	17.54560
O	19.52595	21.31731	18.21453
C	19.27255	22.11156	19.36843
N	20.00316	23.37541	19.32247
C	19.53515	24.63986	19.01003
N	20.48563	25.56219	19.06554
C	21.63963	24.87719	19.41508
C	22.98016	25.32847	19.67059
O	23.39102	26.50264	19.77496
N	23.86371	24.24301	19.80403
C	23.50769	22.91133	19.80508
N	24.51947	22.01119	19.85990
N	22.23549	22.49689	19.76734
C	21.36716	23.50069	19.54544

C	17.24107	21.07713	18.65714
C	17.74616	22.25924	19.48711
O	17.35253	19.86244	19.42778
P	16.05292	19.19992	20.22822
O	16.60391	17.81866	20.68395
O	14.77560	19.34247	19.45425
O	15.93895	20.17411	21.59400
C	16.60415	19.86001	22.79933
C	18.11837	19.93089	22.75382
O	18.57772	21.25892	22.50348
C	19.96430	21.22616	22.77996
N	20.44039	22.61092	22.85028
C	19.60441	23.68193	22.61898
C	20.05320	24.97300	22.56741
C	19.17832	26.15027	22.26307
C	21.47663	25.21114	22.78706
O	21.97171	26.35422	22.82986
N	22.26218	24.07708	22.96244
C	21.83203	22.76753	22.99356
O	22.57547	21.80047	23.15460
C	18.81085	19.51217	24.07696
C	20.11230	20.37342	24.05609
O	18.97691	18.12638	24.22523
O	31.96751	27.45668	22.42062
C	32.80279	26.45529	22.94436
C	32.24015	25.03177	22.84654
O	31.19303	24.78142	23.80792
C	30.12944	24.07336	23.19093
N	28.97006	24.97572	23.09247
C	28.97664	26.36373	23.08653
N	27.76157	26.89269	23.10648
C	26.91966	25.80004	23.12296
C	25.50421	25.68548	23.13559
N	24.68684	26.75480	23.17200
N	24.98545	24.42931	23.12838
C	25.80264	23.35296	23.11212
N	27.14036	23.34245	23.11246
C	27.64290	24.59103	23.10969
C	31.61523	24.70651	21.48213
C	30.65426	23.57709	21.83512
O	32.59762	24.40884	20.52479
P	33.14254	25.68188	19.53425
O	34.64927	25.68179	19.58997
O	32.27363	26.91673	19.83645
O	32.57831	25.13315	18.05754
C	32.84971	23.78124	17.70633
C	31.58327	23.09025	17.20201
O	30.56021	23.17345	18.21434
C	29.41972	23.83171	17.66993
N	28.70093	24.60400	18.68737
C	29.16569	25.81545	19.12561
C	28.34156	26.68581	19.79164
C	26.96259	26.28834	19.95108
N	26.01062	27.16879	20.30657
N	26.59380	25.01630	19.73614
C	27.42606	24.11986	19.13927
O	27.11435	22.93768	18.93174
C	30.97111	23.67620	15.90584
C	29.96029	24.66062	16.50165
O	30.32888	22.62161	15.19866

P	30.24511	22.63825	13.49558
O	29.85496	21.22781	13.10254
O	31.40612	23.43253	12.94344
O	28.85181	23.63366	13.26662
C	27.82307	23.59828	14.21655
C	26.50971	22.90324	13.81993
O	25.84482	23.71287	12.79632
C	25.80156	22.97697	11.55762
N	25.99961	23.89066	10.45464
C	27.13271	24.63443	10.13267
N	26.91276	25.52390	9.18330
C	25.56918	25.38472	8.87198
C	24.71700	26.14240	7.97890
O	24.97843	27.04372	7.18305
N	23.36183	25.70483	8.13445
C	22.88228	24.74891	9.00222
N	21.51869	24.56899	9.03024
N	23.68447	24.00320	9.74766
C	24.97936	24.37431	9.66152
C	26.67296	21.49275	13.17724
C	26.83155	21.86469	11.70058
O	25.57577	20.63311	13.40305
O	22.06716	17.65078	20.15869
O	22.72821	18.16283	18.06861
O	18.24152	16.66711	18.91607
O	18.83348	16.96375	21.79962
O	19.95848	17.81820	17.17195
O	22.40033	15.85252	22.13166
O	21.89775	22.57257	16.34386
O	25.81122	22.93207	15.05044
O	26.38121	28.85082	17.56168
C	19.34606	17.37739	19.44608
C	19.83687	16.85288	20.79564
C	20.46170	17.34386	18.40265
C	21.08632	17.66514	21.20249
C	21.61600	18.20166	18.92246
C	21.82289	17.11370	22.42196
C	23.11814	19.31286	17.41944
C	22.22963	20.35363	17.11982
C	24.48330	19.40597	17.06681
C	22.73896	21.49068	16.48399
C	24.07620	21.58047	16.06561
C	24.95229	20.52963	16.38024
C	24.40995	22.80009	15.24008
C	23.79396	24.00971	15.97563
C	22.49244	23.79776	16.33983
C	24.47916	25.28133	16.30935
C	25.88386	25.35872	16.45485
C	23.75155	26.46784	16.59834
C	26.52434	26.54728	16.81649
C	24.38216	27.64933	16.99676
C	25.78156	27.69802	17.11630
H	20.40051	26.50979	17.57614
H	18.28497	26.91486	17.23943
H	18.98665	28.46317	16.60667
H	17.32016	27.39172	15.13651
H	18.88633	27.42870	12.03914
H	21.20160	26.71342	14.71672
H	23.68664	26.50270	14.26494
H	24.98396	26.60520	10.80067

H	25.36260	26.25577	12.46090
H	19.16680	24.99382	15.24127
H	19.10994	24.72612	12.79900
H	17.55385	25.61281	12.73950
H	18.93748	21.64701	15.61101
H	17.13371	21.61129	15.82637
H	18.39282	19.92327	17.21703
H	19.69643	21.56133	20.23087
H	18.49962	24.83010	18.67010
H	25.47542	22.30083	19.56950
H	24.24329	21.04738	19.67003
H	16.20676	21.20782	18.27784
H	17.40345	22.17865	20.53332
H	17.38260	23.20649	19.04770
H	16.35309	18.83117	23.14791
H	16.23877	20.58462	23.56200
H	18.48410	19.25967	21.94718
H	20.52394	20.74529	21.93985
H	18.55196	23.41314	22.46195
H	18.11876	25.84870	22.15134
H	19.50638	26.62255	21.31328
H	19.26016	26.92361	23.05569
H	18.17661	19.86292	24.92684
H	20.18994	21.03080	24.94388
H	21.01688	19.74137	24.01838
H	18.98259	17.69654	23.32072
H	32.05326	27.37323	21.41210
H	33.79276	26.43144	22.42373
H	32.99890	26.67867	24.01856
H	33.07804	24.31273	23.02497
H	29.79266	23.23668	23.84020
H	29.92880	26.91773	23.04053
H	25.13171	27.65099	22.98180
H	23.65914	26.63692	23.00402
H	25.27862	22.38085	23.08831
H	31.02131	25.58948	21.16283
H	31.21615	22.63064	21.97816
H	29.85957	23.40003	21.08793
H	33.62777	23.73999	16.90884
H	33.22977	23.21872	18.58480
H	31.81896	22.02045	17.00350
H	28.67972	23.07518	17.33141
H	30.22262	26.06042	18.92131
H	28.72050	27.64686	20.16095
H	26.25941	28.15558	20.29838
H	24.99668	26.90652	20.15657
H	31.72361	24.16262	15.24819
H	29.18812	24.98915	15.78685
H	30.51540	25.53859	16.88520
H	28.15424	23.10857	15.15377
H	27.52431	24.64315	14.45535
H	24.77659	22.56930	11.40803
H	28.07396	24.47950	10.68084
H	20.99611	25.45318	9.24701
H	21.29243	23.87893	9.75369
H	27.60087	21.00996	13.54793
H	26.62322	21.00338	11.03850
H	27.86256	22.23127	11.53571
H	24.75719	21.18327	13.45303
H	17.44968	17.02418	19.41844

H	17.95595	17.27021	21.38420
H	19.01869	17.52542	17.20659
H	22.80286	15.98721	21.24769
H	27.33346	28.65122	17.65015
H	19.06517	18.44974	19.57834
H	20.14116	15.78436	20.69230
H	20.85680	16.29948	18.30461
H	20.76266	18.71492	21.40602
H	21.27538	19.25115	19.08393
H	22.59377	17.86590	22.72389
H	21.11296	17.00262	23.26596
H	21.17584	20.35577	17.42095
H	25.15507	18.57800	17.33411
H	26.00383	20.60698	16.07702
H	21.80820	24.57127	16.71496
H	26.47982	24.45383	16.32216
H	22.65430	26.48728	16.53531
H	27.62090	26.56317	16.92050
H	23.78188	28.53334	17.25364
H	22.68394	26.26192	7.60928
H	24.89378	24.50088	19.84529
H	23.33609	24.21130	23.04969
O	23.73270	22.61509	13.98701
H	23.91603	23.43008	13.46727

B1

O	21.08096	27.43500	17.66423
C	19.90988	27.98557	17.09291
C	19.53162	27.35307	15.76253
O	20.60678	27.49135	14.81415
C	20.36307	26.52490	13.80773
N	21.58634	26.33111	13.02854
C	22.76001	26.13577	13.68026
C	23.91291	25.81144	13.00520
C	23.77171	25.64895	11.57836
N	24.84575	25.24249	10.84671
N	22.64035	25.89116	10.92195
C	21.49797	26.25851	11.58280
O	20.42533	26.50235	11.03407
C	19.14927	25.84144	15.78820
C	19.89295	25.27084	14.56622
O	17.75225	25.65577	15.63326
P	16.79985	25.44182	17.01755
O	15.40393	25.16354	16.52491
O	17.20351	26.46166	18.07622
O	17.50785	24.01713	17.61008
C	17.30193	22.77155	16.98989
C	17.73064	21.66865	17.96078
O	19.06638	21.93543	18.44841
C	19.00075	22.48716	19.77443
N	19.79267	23.69894	19.87134
C	19.43656	25.03018	19.68043
N	20.47633	25.84196	19.78089
C	21.56323	25.02276	20.02822
C	22.95867	25.32150	20.13843
O	23.48742	26.45149	20.17625
N	23.73836	24.14937	20.16101
C	23.24660	22.86213	20.11322
N	24.15276	21.85730	20.01825
N	21.93946	22.57794	20.13322
C	21.16550	23.67718	20.05277

C	16.90040	21.54924	19.24881
C	17.51863	22.63503	20.12984
O	17.19177	20.26300	19.81693
P	16.08442	19.45573	20.77227
O	16.79218	18.09960	21.03634
O	14.69847	19.56181	20.20644
O	16.13536	20.35030	22.19256
C	16.93905	19.98077	23.29612
C	18.43052	20.19656	23.12349
O	18.75847	21.58240	22.97584
C	20.15767	21.63973	23.18232
N	20.54914	23.04792	23.29615
C	19.64135	24.07429	23.15081
C	20.00743	25.39320	23.13663
C	19.04843	26.52263	22.91898
C	21.42283	25.71170	23.29562
O	21.86100	26.87823	23.33204
N	22.27566	24.62089	23.41944
C	21.93064	23.28713	23.40047
O	22.74618	22.36778	23.48272
C	19.28059	19.68643	24.32252
C	20.40808	20.75678	24.41410
O	19.71554	18.35716	24.18466
O	31.86643	28.16418	22.50869
C	32.73589	27.29650	23.18846
C	32.20559	25.86793	23.35609
O	31.16816	25.79447	24.35836
C	30.11331	24.96616	23.90773
N	28.93201	25.80729	23.64685
C	28.89429	27.16473	23.36953
N	27.65903	27.63708	23.25099
C	26.85455	26.53671	23.46280
C	25.44245	26.37198	23.46463
N	24.58225	27.39122	23.30988
N	24.97408	25.10712	23.63120
C	25.82828	24.07793	23.81766
N	27.16388	24.12450	23.88417
C	27.61912	25.37725	23.69234
C	31.56825	25.28381	22.08661
C	30.62711	24.23081	22.66100
O	32.53385	24.81136	21.17804
P	33.01184	25.86347	19.92638
O	34.50380	25.73701	19.77716
O	32.22651	27.16710	20.08059
O	32.23916	25.06753	18.64242
C	32.69323	23.75118	18.39072
C	31.62904	22.96507	17.63768
O	30.48079	22.75453	18.46509
C	29.30843	23.33323	17.89962
N	28.56687	24.10521	18.89422
C	29.13977	25.23791	19.41913
C	28.39640	26.15645	20.09986
C	26.97261	25.92750	20.14317
N	26.12994	26.90566	20.48134
N	26.46272	24.72496	19.80510
C	27.21784	23.75777	19.22048
O	26.78467	22.62714	18.93623
C	31.13152	23.63033	16.33752
C	29.79214	24.22118	16.73983
O	31.05076	22.54972	15.33998

P	30.41038	22.65193	13.87969
O	30.75364	21.50924	12.98532
O	30.92967	24.12220	13.24789
O	28.84432	22.98577	14.10327
C	27.73610	22.21475	13.61340
C	27.34503	22.59574	12.19152
O	27.14717	24.01764	12.07327
C	28.31695	24.64990	11.54257
N	29.01974	25.43258	12.55464
C	30.16821	25.24625	13.34132
N	30.41915	26.23271	14.15884
C	29.38007	27.12578	13.96086
C	29.03706	28.35432	14.62639
O	29.62774	28.97962	15.51957
N	27.77764	28.83532	14.15823
C	26.90495	28.16905	13.33008
N	25.63541	28.66496	13.21784
N	27.26419	27.08785	12.64867
C	28.47785	26.62850	13.00341
C	28.29623	22.25523	11.02026
C	29.13385	23.54389	10.84722
O	27.56023	21.91981	9.84949
O	21.86039	18.88153	19.29467
O	21.92944	19.23424	17.06079
O	18.16488	17.01317	18.98903
O	19.31024	17.57039	21.62202
O	19.16664	18.35105	16.86064
O	23.02030	17.43605	21.25766
O	22.28830	24.00754	17.01912
O	26.20174	23.82675	15.81997
O	27.38453	29.98934	16.79169
C	19.20413	17.94922	19.21949
C	20.08359	17.64592	20.43208
C	20.03056	18.05789	17.93959
C	21.13471	18.77578	20.53163
C	21.04740	19.17284	18.15610
C	22.20416	18.54456	21.59621
C	22.62316	20.39907	16.87808
C	22.04422	21.66570	17.02287
C	23.98708	20.26293	16.51194
C	22.86662	22.78395	16.83023
C	24.22772	22.68605	16.47499
C	24.77143	21.39080	16.31422
C	25.05243	23.89108	16.30709
C	24.40240	25.14573	16.73908
C	23.05472	25.11691	17.02250
C	25.15027	26.42066	16.81317
C	26.55635	26.40338	16.95998
C	24.51235	27.68588	16.78953
C	27.30344	27.57727	17.04434
C	25.26228	28.87137	16.81962
C	26.66943	28.82592	16.91293
H	20.83850	27.03079	18.55233
H	19.01967	27.84002	17.74610
H	20.05365	29.08151	16.92738
H	18.62426	27.87640	15.37283
H	19.60046	26.86897	13.07500
H	22.72004	26.26284	14.77139
H	24.85269	25.60805	13.53668
H	24.65915	25.05084	9.86311

H	25.67499	24.85679	11.31485	H	29.80053	24.26326	24.70945
H	19.51220	25.36333	16.72294	H	29.82849	27.73548	23.23584
H	20.77119	24.67194	14.87847	H	24.97587	28.31517	23.14746
H	19.21903	24.63282	13.96537	H	23.54679	27.22842	23.26519
H	17.91914	22.68387	16.06280	H	25.33988	23.09135	23.91450
H	16.23488	22.64068	16.69329	H	30.96086	26.08703	21.61813
H	17.76514	20.69017	17.43589	H	31.20045	23.33120	22.96676
H	19.49687	21.76086	20.44964	H	29.81379	23.92054	21.97916
H	18.42617	25.36382	19.38479	H	33.63959	23.76176	17.79983
H	25.11708	22.06750	19.69673	H	32.89688	23.21269	19.34282
H	23.75144	20.94596	19.79045	H	32.06583	21.97611	17.36814
H	15.80812	21.65442	19.08412	H	28.60362	22.53974	17.57527
H	17.32957	22.44141	21.19966	H	30.21970	25.36234	19.23606
H	17.13349	23.62399	19.82653	H	28.85613	27.07613	20.48134
H	16.80701	18.90339	23.54757	H	26.51934	27.76595	20.86469
H	16.58877	20.58756	24.16119	H	25.08501	26.75361	20.41007
H	18.77304	19.65136	22.21879	H	31.82566	24.39536	15.93958
H	20.70756	21.23287	22.29925	H	29.04375	24.29453	15.93254
H	18.60068	23.74712	23.02933	H	30.00439	25.24450	17.10447
H	18.00541	26.16018	22.83920	H	27.95362	21.12722	13.67146
H	19.30612	27.05250	21.97833	H	26.91282	22.48060	14.30898
H	19.11564	27.26810	23.73886	H	26.36484	22.10893	11.99616
H	18.66487	19.74385	25.25265	H	27.93029	25.41476	10.83823
H	20.30900	21.37497	25.32871	H	25.26353	29.11166	14.06351
H	21.41186	20.29890	24.40517	H	24.97839	27.98185	12.81817
H	19.62973	18.07088	23.23227	H	28.94369	21.38727	11.24852
H	31.95947	27.91354	21.53224	H	29.24723	23.75376	9.76707
H	33.72221	27.20763	22.66852	H	30.14571	23.42838	11.26978
H	32.93662	27.71124	24.20256	H	26.90570	22.63255	9.71533
H	33.05485	25.20742	23.66021	H	17.44549	17.28714	19.62926

H	18.33184	17.75509	21.41445
H	18.33670	17.89720	17.13720
H	23.21481	17.57077	20.30739
H	28.33595	29.74215	16.61531
H	18.75490	18.95388	19.38907
H	20.62754	16.68288	20.28931
H	20.59900	17.10841	17.76418
H	20.60415	19.73697	20.73276
H	20.51134	20.13699	18.31057
H	22.79594	19.48621	21.71545
H	21.71057	18.33620	22.56731
H	20.99549	21.81728	17.32059
H	24.39798	19.24651	16.43151
H	25.84243	21.31280	16.08072
H	22.43491	26.00118	17.26228
H	27.07322	25.44087	16.99827
H	23.41302	27.76224	16.76111
H	28.39416	27.52891	17.17254
H	24.76108	29.85116	16.77887
H	27.42995	29.62713	14.71873
H	24.79027	24.31514	20.08218
H	23.33639	24.81328	23.50855

B1-OHS

O	20.97459	27.43910	17.43188
C	19.79439	27.92102	16.82198
C	19.41555	27.17258	15.55116
O	20.47248	27.26015	14.58269
C	20.27660	26.18323	13.67835
N	21.50625	25.97199	12.92187
C	22.68083	25.84410	13.59666
C	23.85239	25.55118	12.94716
C	23.75090	25.38130	11.51632
N	24.85883	25.02917	10.81960
N	22.61570	25.55722	10.83854
C	21.44449	25.85661	11.47841
O	20.36335	26.02009	10.91023
C	19.07220	25.65953	15.71039
C	19.85848	24.99807	14.56585
O	17.68062	25.42508	15.54316
P	16.70583	25.31848	16.92032
O	15.31844	24.99535	16.42659
O	17.07928	26.41742	17.90820
O	17.40702	23.94660	17.63607
C	17.30415	22.67104	17.05437
C	17.77874	21.63242	18.07204
O	19.10706	21.95977	18.53780
C	19.03744	22.56595	19.83921
N	19.79409	23.80255	19.87537
C	19.40742	25.10754	19.58776
N	20.42695	25.94951	19.63680
C	21.53357	25.17833	19.94681
C	22.92518	25.51591	20.03360
O	23.43407	26.65356	19.97815
N	23.72696	24.36547	20.15639
C	23.26371	23.06876	20.22469
N	24.19236	22.08259	20.25032
N	21.96081	22.75807	20.25183
C	21.16682	23.82914	20.07011
C	16.95122	21.55447	19.36519
C	17.55277	22.68403	20.20097

O	17.24964	20.29694	19.99462
P	16.08724	19.44293	20.83123
O	16.81134	18.10745	21.15185
O	14.75360	19.50392	20.14348
O	15.96555	20.32522	22.25560
C	16.72857	20.01543	23.40505
C	18.22187	20.24549	23.28937
O	18.55274	21.62902	23.12298
C	19.95108	21.66294	23.34165
N	20.39540	23.05532	23.37266
C	19.53117	24.11240	23.18706
C	19.95911	25.40838	23.08544
C	19.05094	26.57199	22.83085
C	21.39455	25.66585	23.18479
O	21.88526	26.81022	23.14111
N	22.20011	24.54478	23.34507
C	21.79057	23.23487	23.43050
O	22.55815	22.28051	23.56429
C	19.03437	19.76463	24.52737
C	20.15940	20.83783	24.61990
O	19.46198	18.42941	24.43482
O	32.03366	27.60314	22.75424
C	32.76068	26.66764	23.50365
C	32.17461	25.24937	23.50301
O	31.01995	25.13231	24.35950
C	30.00177	24.39232	23.70559
N	28.89205	25.30724	23.39358
C	28.94410	26.68194	23.21045
N	27.74596	27.23973	23.10261
C	26.86907	26.18141	23.22002
C	25.45029	26.10799	23.22607
N	24.65566	27.18759	23.12239
N	24.90040	24.87320	23.36909
C	25.68371	23.78127	23.50880
N	27.01911	23.73763	23.54788
C	27.55497	24.96314	23.39565
C	31.70060	24.78165	22.12057
C	30.65041	23.73470	22.47821
O	32.78428	24.34668	21.33828
P	33.29084	25.38916	20.08547
O	34.75846	25.12639	19.87929
O	32.66099	26.76307	20.33789
O	32.38240	24.72698	18.82906
C	32.69034	23.37181	18.53074
C	31.53534	22.70792	17.79690
O	30.37116	22.70304	18.63516
C	29.27161	23.33132	17.98549
N	28.54981	24.20014	18.92091
C	29.13220	25.36821	19.34369
C	28.38721	26.36024	19.91609
C	26.96318	26.14419	19.97792
N	26.11552	27.14930	20.23108
N	26.45027	24.91558	19.77438
C	27.21025	23.89044	19.29829
O	26.77821	22.73243	19.16065
C	31.13886	23.35888	16.45404
C	29.86150	24.11596	16.80359
O	30.92378	22.30274	15.49582
P	30.89786	22.67376	13.88531
O	30.84873	21.39028	13.09903

O	31.99066	23.75958	13.65325	C	20.15281	17.74367	20.67327
O	29.49104	23.55168	13.70962	C	20.20385	18.11596	18.17654
C	28.26233	22.84331	13.56103	C	21.18044	18.89141	20.79701
C	27.66053	23.09901	12.18962	C	21.20727	19.24263	18.41089
O	27.33887	24.49530	12.00160	C	22.19524	18.70298	21.92242
C	28.37521	25.17418	11.26010	C	22.81226	20.45373	17.14406
N	29.00947	26.19023	12.07788	C	22.19085	21.70666	17.17689
C	30.34382	26.34479	12.60435	C	24.19462	20.33665	16.85151
N	30.41972	27.39212	13.41334	C	22.99172	22.83630	16.95210
C	29.15392	27.93678	13.44698	C	24.37135	22.75885	16.68106
C	28.61576	29.02588	14.19911	C	24.95795	21.47418	16.62169
O	29.15244	29.75947	15.06560	C	25.18352	23.97575	16.49637
N	27.23944	29.22580	13.90020	C	24.46924	25.24592	16.76445
C	26.43615	28.38186	13.17066	C	23.10666	25.18240	16.96440
N	25.07984	28.64484	13.18693	C	25.15272	26.56233	16.78254
N	26.92821	27.37846	12.46457	C	26.56579	26.65333	16.74770
C	28.26173	27.21163	12.62616	C	24.42874	27.78044	16.87652
C	28.52944	22.75730	10.96510	C	27.22889	27.88256	16.76582
C	29.29080	24.06836	10.70351	C	25.08788	29.01556	16.85933
O	27.71412	22.36305	9.85560	C	26.49730	29.08680	16.76952
O	21.96418	18.97770	19.59817	H	20.73986	27.06431	18.33493
O	22.13377	19.27818	17.35864	H	18.91012	27.82433	17.49206
O	18.30818	17.05753	19.16066	H	19.92502	28.99971	16.56013
O	19.33067	17.67193	21.83187	H	18.48781	27.63938	15.13663
O	19.38241	18.37956	17.05668	H	19.50161	26.41697	12.91551
O	23.05308	17.60811	21.64676	H	22.62648	26.01373	14.68157
O	22.37124	24.05041	17.01963	H	24.80173	25.43540	13.48727
O	26.37432	23.89234	16.13933	H	24.76825	25.02863	9.80485
O	27.08765	30.30414	16.61189	H	25.78047	24.95856	11.28722
C	19.32276	18.01571	19.42021	H	19.42176	25.28047	16.69398

H	20.75995	24.48458	14.95366
H	19.22877	24.26264	14.03180
H	17.94811	22.59421	16.14505
H	16.25603	22.45366	16.73915
H	17.83970	20.63260	17.59168
H	19.56627	21.88608	20.53889
H	18.39024	25.39341	19.26866
H	25.16038	22.28105	19.91686
H	23.81315	21.14651	20.09809
H	15.85898	21.63976	19.19067
H	17.37685	22.52122	21.27797
H	17.14041	23.65093	19.86444
H	16.60624	18.94525	23.69275
H	16.33012	20.64779	24.23048
H	18.60548	19.68415	22.41179
H	20.49363	21.18087	22.49377
H	18.47390	23.82758	23.10585
H	17.99146	26.25530	22.77267
H	19.32534	27.05447	21.86969
H	19.15942	27.34478	23.62115
H	18.39381	19.84219	25.43858
H	20.02387	21.49757	25.50074
H	21.16373	20.38361	24.66804
H	19.50783	18.16007	23.47477
H	32.24651	27.38853	21.78214
H	33.81176	26.56907	23.13262
H	32.81790	27.01768	24.56055
H	32.96393	24.54095	23.85845
H	29.56404	23.64026	24.39677
H	29.91498	27.20293	23.15302
H	25.10844	28.06760	22.88702
H	23.61752	27.08599	23.05296
H	25.12884	22.82979	23.59477
H	31.18996	25.63786	21.62952
H	31.14449	22.78904	22.78484
H	29.93683	23.51025	21.66383
H	33.61400	23.30357	17.91031
H	32.87291	22.80461	19.47047
H	31.83330	21.65701	17.57524
H	28.52486	22.57422	17.67034
H	30.22002	25.45494	19.18242
H	28.85063	27.30145	20.23363
H	26.49775	28.08195	20.36419
H	25.07658	26.99415	20.14572
H	31.92710	24.03595	16.06752
H	29.16650	24.20732	15.95157
H	30.14349	25.13667	17.12439
H	28.42161	21.74855	13.67626
H	27.55616	23.19049	14.34517
H	26.70123	22.53870	12.13021
H	27.83293	25.72669	10.45868
H	24.72582	28.93382	14.10779
H	24.54254	27.84534	12.82927
H	29.22397	21.92009	11.17770
H	29.46819	24.19039	9.61765
H	30.26535	24.09739	11.21859
H	26.97935	23.00596	9.82175
H	17.56023	17.32045	19.77135
H	18.35717	17.81581	21.57993
H	18.55017	17.91369	17.30261

H	23.30352	17.74028	20.70897
H	28.00365	30.16322	16.18661
H	18.85106	19.01537	19.55515
H	20.71809	16.78802	20.56818
H	20.78789	17.16917	18.04180
H	20.62343	19.84712	20.94845
H	20.66254	20.20828	18.52096
H	22.75536	19.66018	22.06182
H	21.65292	18.49312	22.86728
H	21.12368	21.84215	17.40626
H	24.63677	19.33018	16.84289
H	26.03783	21.42083	16.42424
H	22.44166	26.05368	17.09680
H	27.15023	25.72972	16.68593
H	23.33110	27.78656	16.96994
H	28.32756	27.91456	16.72411
H	24.51330	29.95414	16.90570
H	26.80046	29.94573	14.48667
H	24.77980	24.54211	20.06815
H	23.28258	24.68778	23.36211
O	31.30962	25.57234	12.21831
H	31.66482	24.71857	12.96993

B1-OHST

O	21.02587	27.40478	17.61759
C	19.88637	27.87371	16.92479
C	19.56305	27.09698	15.65512
O	20.66636	27.12769	14.73650
C	20.41379	26.07744	13.81077
N	21.61771	25.83521	13.02588
C	22.78605	25.56420	13.66436
C	23.95117	25.33943	12.97355
C	23.85881	25.42885	11.53385
N	24.96874	25.23999	10.78340
N	22.71737	25.70030	10.89695
C	21.54913	25.90334	11.57550
O	20.46529	26.13960	11.04043
C	19.15953	25.59766	15.81752
C	19.93666	24.90402	14.68246
O	17.76378	25.41744	15.62121
P	16.73066	25.39397	16.95940
O	15.34687	25.15781	16.40985
O	17.13366	26.47752	17.95229
O	17.30661	23.98577	17.71590
C	17.19359	22.72404	17.10621
C	17.66700	21.65949	18.09719
O	19.00033	21.96948	18.55992
C	18.94300	22.53610	19.87954
N	19.71773	23.75883	19.95351
C	19.35291	25.07921	19.70809
N	20.38341	25.90384	19.79897
C	21.47543	25.10520	20.09338
C	22.87118	25.41738	20.20789
O	23.39448	26.54966	20.22040
N	23.65749	24.25092	20.26971
C	23.17508	22.95943	20.25942
N	24.08595	21.95858	20.19871
N	21.86704	22.66746	20.28736
C	21.08923	23.75753	20.15716
C	16.84840	21.55627	19.39453
C	17.46214	22.66472	20.24938

O	17.14764	20.28244	19.98896
P	16.02588	19.46271	20.91112
O	16.74396	18.11801	21.20411
O	14.65280	19.54156	20.30765
O	16.01287	20.36380	22.32914
C	16.81504	20.03128	23.44534
C	18.30454	20.26316	23.28313
O	18.62495	21.64972	23.13189
C	20.02813	21.70277	23.32001
N	20.43291	23.10722	23.41384
C	19.54233	24.14309	23.23214
C	19.93010	25.45496	23.18654
C	18.99226	26.59451	22.93160
C	21.35117	25.75562	23.34330
O	21.80729	26.91536	23.35238
N	22.18581	24.65535	23.49773
C	21.81872	23.32912	23.51738
O	22.61676	22.39804	23.63407
C	19.14904	19.76879	24.49198
C	20.28942	20.82792	24.55629
O	19.55743	18.42853	24.38712
O	31.90222	27.86980	22.47797
C	32.72008	26.97064	23.17767
C	32.13809	25.55952	23.32400
O	31.07948	25.50774	24.30546
C	30.00540	24.72338	23.81873
N	28.85892	25.61124	23.55945
C	28.87253	26.97143	23.29367
N	27.65671	27.49642	23.20103
C	26.81030	26.42770	23.41293
C	25.39378	26.31788	23.44625
N	24.56675	27.36997	23.31455
N	24.87988	25.07425	23.63488
C	25.69496	24.01056	23.80613
N	27.03134	24.00443	23.83701
C	27.53167	25.23656	23.62588
C	31.51373	25.00986	22.03284
C	30.51779	23.98828	22.57169
O	32.49080	24.51573	21.15439
P	32.98894	25.54435	19.88377
O	34.47872	25.37381	19.74374
O	32.26977	26.88753	20.06798
O	32.16982	24.80361	18.61831
C	32.53616	23.45360	18.34644
C	31.40756	22.71572	17.63890
O	30.26127	22.64297	18.50349
C	29.12969	23.24999	17.89262
N	28.42139	24.09070	18.87106
C	29.03493	25.22061	19.34128
C	28.33299	26.18428	20.01074
C	26.90892	25.99249	20.10070
N	26.08941	26.99952	20.42897
N	26.36435	24.79021	19.82888
C	27.08859	23.78382	19.26283
O	26.62388	22.64676	19.06211
C	30.93258	23.32010	16.29980
C	29.66096	24.06604	16.70518
O	30.65563	22.25584	15.39184
P	30.72952	22.62006	13.74774
O	30.50369	21.30533	13.02169

O	31.86335	23.59417	13.49068
O	29.31566	23.57259	13.58148
C	28.13111	22.94083	13.14554
C	27.86642	23.19273	11.66542
O	27.64747	24.60730	11.37417
C	28.83950	25.22696	10.91669
N	29.29866	26.23297	11.88476
C	30.67259	26.55778	12.11748
N	30.64141	27.46504	13.17116
C	29.33553	27.80802	13.48503
C	28.79636	28.78252	14.36133
O	29.37477	29.50029	15.21393
N	27.39760	28.88499	14.18139
C	26.61910	28.01667	13.45506
N	25.26458	28.16913	13.57394
N	27.13976	27.10430	12.64242
C	28.48855	27.04367	12.66163
C	28.98254	22.82153	10.67829
C	29.85301	24.08834	10.66832
O	28.43988	22.52077	9.38371
O	21.84638	18.92998	19.48680
O	21.93806	19.24118	17.24375
O	18.16336	17.03910	19.17417
O	19.27703	17.64986	21.80803
O	19.17622	18.35177	17.03361
O	22.98835	17.53781	21.49512
O	22.31186	24.01524	17.15117
O	26.22548	23.80349	15.96205
O	27.30355	30.06761	16.88478
C	19.19460	17.98849	19.39765
C	20.06270	17.71188	20.62371
C	20.03361	18.08088	18.12453
C	21.10523	18.84967	20.71319
C	21.04956	19.20166	18.32778
C	22.15816	18.64572	21.80031
C	22.63917	20.40596	17.04239
C	22.06245	21.67328	17.18100
C	23.99559	20.26359	16.65537
C	22.88717	22.78801	16.97413
C	24.24293	22.68399	16.61055
C	24.78083	21.38893	16.43689
C	25.07903	23.88605	16.43710
C	24.43103	25.15277	16.85466
C	23.08146	25.12545	17.12770
C	25.17272	26.43374	16.92628
C	26.58819	26.44897	16.90001
C	24.51197	27.68123	17.07156
C	27.31565	27.63820	16.97592
C	25.23719	28.88046	17.10426
C	26.64798	28.87571	17.02210
H	20.73980	27.03680	18.50728
H	18.96469	27.80666	17.54635
H	20.04499	28.94217	16.63834
H	18.67171	27.58006	15.18176
H	19.63975	26.36683	13.06599
H	22.74727	25.56861	14.76299
H	24.88594	25.08915	13.49409
H	24.85312	25.40048	9.78346
H	25.92185	25.18956	11.18178
H	19.47631	25.20781	16.80836

H	20.80629	24.34691	15.08142
H	19.28144	24.20188	14.13455
H	17.83251	22.66457	16.19244
H	16.14337	22.51789	16.78921
H	17.71726	20.66985	17.59458
H	19.46039	21.82644	20.55728
H	18.34628	25.38841	19.37858
H	25.04771	22.16683	19.84966
H	23.68568	21.04481	19.97747
H	15.75472	21.64993	19.23280
H	17.28796	22.48814	21.32460
H	17.05935	23.64105	19.92861
H	16.69900	18.95689	23.71814
H	16.44859	20.65045	24.29524
H	18.66097	19.71423	22.38591
H	20.56194	21.28071	22.43428
H	18.49777	23.82912	23.10847
H	17.94387	26.24741	22.85079
H	19.26801	27.09482	21.97986
H	19.06506	27.35982	23.73297
H	18.53478	19.85528	25.42073
H	20.21380	21.45487	25.46728
H	21.28872	20.36124	24.53385
H	19.54136	18.14895	23.42864
H	32.00717	27.60675	21.49931
H	33.71538	26.84568	22.68178
H	32.91045	27.37441	24.19939
H	32.95926	24.86799	23.63756
H	29.65205	24.01909	24.60263
H	29.82850	27.50388	23.15277
H	24.98862	28.26321	23.07194
H	23.53064	27.23580	23.25946
H	25.16783	23.04626	23.92181
H	30.94667	25.83695	21.55456
H	31.04829	23.06235	22.87693
H	29.71398	23.71794	21.86213
H	33.45479	23.41940	17.71669
H	32.75429	22.91925	19.29747
H	31.76383	21.68265	17.42239
H	28.39150	22.48075	17.58851
H	30.11319	25.30824	19.12722
H	28.82668	27.10425	20.34569
H	26.50196	27.87716	20.73720
H	25.04677	26.86311	20.38940
H	31.69026	23.99705	15.85000
H	28.94478	24.13647	15.86763
H	29.93265	25.08719	17.03707
H	28.19057	21.83756	13.28726
H	27.27831	23.32888	13.74533
H	26.92276	22.67775	11.38078
H	28.57482	25.77557	9.97978
H	24.93329	28.46896	14.49960
H	24.72793	27.37194	13.20661
H	29.55664	21.94032	11.02962
H	30.37820	24.20093	9.70171
H	30.61677	24.05136	11.46944
H	27.82559	23.25355	9.18518
H	17.43771	17.31345	19.80615
H	18.29852	17.81021	21.58780
H	18.34805	17.89559	17.30976

H	23.21195	17.66770	20.55031
H	28.20911	29.89558	16.47831
H	18.73592	18.99216	19.54625
H	20.61522	16.75073	20.50300
H	20.60596	17.13023	17.96946
H	20.56630	19.81203	20.88671
H	20.51012	20.16701	18.46209
H	22.73964	19.59366	21.91572
H	21.64869	18.44602	22.76525
H	21.01458	21.82963	17.47605
H	24.40225	19.24651	16.56219
H	25.84192	21.31625	16.15810
H	22.45256	26.01073	17.32761
H	27.12438	25.50132	16.79154
H	23.41458	27.73611	17.15530
H	28.41521	27.61312	16.94333
H	24.71437	29.84669	17.17880
H	26.94748	29.54964	14.82196
H	24.71255	24.41968	20.16414
H	23.25727	24.83207	23.56429
O	31.64289	26.19145	11.47485
H	31.48289	27.81025	13.62671

References:

1. Amovilli C, Barone V, Cammi R, et al. Recent Advances in the Description of Solvent Effects with the Polarizable Continuum Model. *Adv Quantum Chem*. 1998;32(C):227-261. doi:10.1016/S0065-3276(08)60416-5

2. Werner HJ, Knowles PJ, Knizia G, Manby FR, Schütz M. Molpro: A general-purpose quantum chemistry program package. *Wiley Interdiscip Rev Comput Mol Sci*. 2012;2(2):242-253. doi:10.1002/WCMS.82;WEBSITE:WEBSITE:WIRES;WGROUP:STRING:PUBLICATION

3. Neese F. Software update: the ORCA program system, version 4.0. *Wiley Interdiscip Rev Comput Mol Sci*. 2018;8(1):e1327. doi:10.1002/WCMS.1327

4. Majumder R, Das CK, Banerjee I, et al. Screening of the Prime bioactive compounds from Aloe vera as potential anti-proliferative agents targeting DNA. *Comput Biol Med*. 2022;141:105052. doi:10.1016/J.COMPBIOMED.2021.105052

5. Perdew JP, Burke K, Ernzerhof M. Generalized Gradient Approximation Made Simple. *Phys Rev Lett*. 1996;77(18):3865. doi:10.1103/PhysRevLett.77.3865

6. Caldeweyher E, Mewes JM, Ehlert S, Grimme S. Extension and evaluation of the D4 London-dispersion model for periodic systems. *Phys Chem Chem Phys*. 2020;22(16):8499-8512. doi:10.1039/D0CP00502A

7. Weigend F, Ahlrichs R. Balanced basis sets of split valence, triple zeta valence and quadruple zeta valence quality for H to Rn: Design and assessment of accuracy. *Phys Chem Chem Phys*. 2005;7(18):3297-3305. doi:10.1039/B508541A

8. Weigend F. Accurate Coulomb-fitting basis sets for H to Rn. *Phys Chem Chem Phys*. 2006;8(9):1057-1065. doi:10.1039/B515623H

9. Kalinowski J, Wennmohs F, Neese F. Arbitrary Angular Momentum Electron Repulsion Integrals with Graphical Processing Units: Application to the Resolution of Identity Hartree–Fock Method. *J Chem Theory Comput*. 2017;13(7):3160-3170. doi:10.1021/ACS.JCTC.7B00030

10. Staroverov VN, Scuseria GE, Tao J, Perdew JP. Comparative assessment of a new nonempirical density functional: Molecules and hydrogen-bonded complexes. *J Chem Phys*. 2003;119(23):12129-12137. doi:10.1063/1.1626543

11. Tao J, Perdew JP, Staroverov VN, Scuseria GE. Climbing the density functional ladder: Nonempirical meta–generalized gradient approximation designed for molecules and solids. *Phys Rev Lett*. 2003;91(14):146401. doi:10.1103/PHYSREVLETT.91.146401/FIGURES/1/MEDIUM

12. Marenich A V., Cramer CJ, Truhlar DG. Universal solvation model based on solute electron density and on a continuum model of the solvent defined by the bulk dielectric constant and atomic surface tensions. *J Phys Chem B*. 2009;113(18):6378-6396. doi:10.1021/JP810292N/SUPPL_FILE/JP810292N_SI_003.PDF

13. Gulaboski R, Mirčeski V, Kappl R, Hoth M, Bozem M. Review—Quantification of Hydrogen Peroxide by Electrochemical Methods and Electron Spin Resonance Spectroscopy. *J Electrochem Soc*. 2019;166(8):G82-G101. doi:10.1149/2.1061908JES/PDF

14. Chatgilialoglu C, Krokidis MG, Masi A, et al. New Insights into the Reaction Paths of Hydroxyl Radicals with Purine Moieties in DNA and Double-Stranded Oligodeoxynucleotides. *Mol 2019, Vol 24, Page 3860*. 2019;24(21):3860. doi:10.3390/MOLECULES24213860

15. Wells PG, Mccallum GP, Chen CS, et al. Oxidative stress in developmental origins of disease: teratogenesis, neurodevelopmental deficits, and cancer. *Toxicol Sci*. 2009;108(1):4-18. doi:10.1093/TOXSCI/KFN263

16. Jain SS, Tullius TD. Footprinting protein-DNA complexes using the hydroxyl radical. *Nat Protoc*. 2008;3(6):1092-1100. doi:10.1038/NPROT.2008.72

17. Andrés CMC, Lastra JMP de la, Juan CA, Plou FJ, Pérez-Lebeña E. Chemical Insights into Oxidative and Nitrative Modifications of DNA. *Int J Mol Sci 2023, Vol 24, Page 15240*. 2023;24(20):15240. doi:10.3390/IJMS242015240

18. Pivetta TP, Ribeiro PA, Raposo M. The Effect of UV-Vis Radiation on DNA Systems Containing the Photosensitizers Methylene Blue and Acridine Orange. *Biophysica*. 2024;4(1):22-33. doi:10.3390/BIOPHYSICA4010002/S1

19. Lu T, Chen F. Multiwfn: A multifunctional wavefunction analyzer. *J Comput Chem*. 2012;33(5):580-592. doi:10.1002/JCC.22885

20. Jmol: an open-source Java viewer for chemical structures in 3D. Accessed October 13, 2025. https://jmol.sourceforge.net/

21. Yuan TT, Chen J, Pham LN, et al. Visible light-mediated syntheses of unsymmetrical methylene-bridged bis-heterocycles via an alkoxy radical relay reaction. *Org Chem Front*. 2023;10(18):4649-4657. doi:10.1039/D3QO00754E

22. Fang C, Fantin M, Pan X, et al. Mechanistically Guided Predictive Models for Ligand and Initiator Effects in Copper-Catalyzed Atom Transfer Radical Polymerization (Cu-ATRP). *J Am Chem Soc*. 2019;141(18):7486-7497. doi:10.1021/JACS.9B02158

23. Marcus RA. On the Theory of Oxidation-Reduction Reactions Involving Electron Transfer. III. Applications to Data on the Rates of Organic Redox Reactions. *J Chem Phys*. 1957;26(4):872-877. doi:10.1063/1.1743424

24. Marcus RA. On the Theory of Oxidation-Reduction Reactions Involving Electron Transfer. II. Applications to Data on the Rates of Isotopic Exchange Reactions. *J Chem Phys*. 1957;26(4):867-871. doi:10.1063/1.1743423

25. López-Estrada O, Laguna HG, Barrueta-Flores C, Amador-Bedolla C. Reassessment of the Four-Point Approach to the Electron-Transfer Marcus–Hush Theory. *ACS Omega*. 2018;3(2):2130-2140. doi:10.1021/ACSOMEGA.7B01425

26. Rosso KM, Dupuis M. Electron transfer in environmental systems: A frontier for theoretical chemistry. *Theor Chem Acc*. 2006;116(1-3):124-136. doi:10.1007/S00214-005-0016-X/METRICS

27. Gotham JP, Li R, Tipple TE, Lancaster JR, Liu T, Li Q. Quantitation of Spin Probe-Detectable Oxidants in Cells Using Electron Paramagnetic Resonance Spectroscopy: To Probe or to Trap? *Free Radic Biol Med*. 2020;154:84. doi:10.1016/J.FREERADBIOMED.2020.04.020
